# Against the use of the Strengths and Difficulties Questionnaire for Aboriginal and Torres Strait Islander children aged 2–15 years

**DOI:** 10.1177/00048674231161504

**Published:** 2023-03-28

**Authors:** Tracey Chau, Jeggan Tiego, Louise Brown, David Coghill, Laura Jobson, Alicia Montgomery, Cammi Murrup-Stewart, Emma Sciberras, Tim J Silk, Megan Spencer-Smith, Nicole Stefanac, Daniel P Sullivan, Mark A Bellgrove

**Affiliations:** 1 Turner Institute for Brain and Mental Health, School of Psychological Sciences, Monash University, Clayton, VIC, Australia; 2 School of Nursing, Midwifery and Paramedicine, Curtin University, Perth, WA, Australia; 3 Department of Paediatrics, The University of Melbourne, Parkville, VIC, Australia; 4 Department of Psychiatry, The University of Melbourne, Parkville, VIC, Australia; 5 Sydney Local Health District, NSW Health, Camperdown, NSW, Australia; 6 School of Psychiatry, University of New South Wales, Sydney, NSW, Australia; 7 Centre for Social and Early Emotional Development, School of Psychology, Deakin University, Burwood, VIC, Australia; 8 Murdoch Children’s Research Institute, Royal Children’s Hospital, Parkville, VIC, Australia; 9 Child and Youth Mental Health Service, Queensland Health, Brisbane, QLD, Australia; 10 Child Health Research Centre, Faculty of Medicine, The University of Queensland, Brisbane, QLD, Australia

**Keywords:** Strengths and Difficulties Questionnaire, Aboriginal, Torres Strait Islander, children, Indigenous

## Abstract

**Objective:**

The Strengths and Difficulties Questionnaire is a widely used screening tool for emotional and behavioural problems in children. Recent quantitative analyses have raised concerns regarding its structural validity in Aboriginal and Torres Strait Islander communities. This paper aims to extend upon existing findings by analysing the factor structure of both the parent- and teacher-reported Strengths and Difficulties Questionnaire in this population across a broader age range than in previous studies.

**Methods:**

Participants were the caregivers and teachers of 1624 Aboriginal and Torres Strait Islander children (820 male, 804 female) aged 2–15 years from Waves 2–11 of the Longitudinal Study of Indigenous Children. The majority of children were Aboriginal living in major cities and inner regional areas. Internal consistency was estimated with McDonald’s Omega. Exploratory structural equation modelling was conducted to investigate the factor structure of the parent-reported and teacher-reported versions of the Strengths and Difficulties Questionnaire.

**Results:**

Responses from teachers demonstrated higher internal consistency than responses from parents, which was unacceptably low across most age groups. The purported five-factor structure of the Strengths and Difficulties Questionnaire failed to be replicated across both parent- and teacher-reported questionnaires. The results of bifactor and hierarchical exploratory structural equation models also failed to approximate the higher-order summary scales. These results indicate that the Strengths and Difficulties Questionnaire subscales and summary scores do not provide a valid index of emotional and behavioural problems in Aboriginal and Torres Strait Islander children.

**Conclusion:**

The Strengths and Difficulties Questionnaire should not be used with Aboriginal and Torres Strait Islander children.

The health and wellbeing of Indigenous populations around the world is frequently assessed and measured against a non-Indigenous benchmark. This wrongly and damagingly shifts the burden of responsibility for health and wellbeing to Indigenous people and their own indigeneity, when it is well known that factors such as a lack of cultural safety, remoteness and isolation and racist systems preclude many Indigenous people from accessing safe and appropriate services (Gentile et al., 2022; Shepherd et al., 2017; Vos et al., 2008). Early evidence indicates that intersectoral approaches that prioritise cultural sensitivity, among other key factors (e.g. involvement of community and collaborative multidisciplinary health services; see Lopez-Carmen et al., 2019), can improve the mental health of Indigenous children around the world, their families and their communities. Accordingly, the development and use of culturally appropriate screening measures for mental health problems are one way to better support Indigenous children to grow up strong and resilient. In this paper, we use the term ‘Indigenous’ to refer to global Indigenous peoples (Chakrabarti, 2006) and the term ‘Aboriginal and Torres Strait Islander’ to refer to the two distinct groups of First Peoples of Australia. We respectfully acknowledge and recognise that each Aboriginal and Torres Strait Islander Nation possesses their own unique cultures and beliefs.

Within Australian Aboriginal and Torres Strait Islander cultures, the concepts of health and wellbeing extend beyond the individual and include the social, emotional, cultural and spiritual wellbeing of their whole community (Swan and Raphael, 1995). This holistic view of health is often described as one’s Social and Emotional Wellbeing (SEWB; Gee et al., 2014). Alongside mental and physical health, SEWB encompasses connections to family and kinship, community, culture, country and spirituality (Gee et al., 2014). The SEWB framework lends itself to a strengths-based approach to health and wellbeing, where the focus is shifted towards the factors that promote good health and resilience (Fogarty et al., 2018b). This contrasts traditional Western deficit–based approaches that aim to identify problems and that inevitably lead to dangerous discourse that wrongly positions an individual’s indigeneity as the primary indicator of poor health (Fogarty et al., 2018a). However, a recent systematic review of measures used to assess SEWB in Aboriginal and Torres Strait Islander peoples concluded that there remains an ongoing need to develop culturally appropriate and psychometrically sound measures of SEWB, as many existing measures – whether adapted or specifically developed – focus on psychological problems (rather than strengths) and lack adequate psychometric data (Newton et al., 2015).

In line with the SEWB framework, the mental health of Aboriginal and Torres Strait Islander peoples should be considered within the context of the other highly interrelated domains of wellbeing. For example, Dudgeon et al. (2020b) highlight the inextricable connections between the body, mind and spirit in Aboriginal and Torres Strait Islander cultures, and how a mentally strong and resilient individual is better able to use appropriate coping strategies to protect and promote their overall wellbeing. This in turn facilitates positive expressions and experiences with the wellbeing domains of community connection, family connection, connection to the land and connection to culture. It is known that Aboriginal and Torres Strait Islander children experience disproportionately poorer mental health outcomes that are uniquely driven by ongoing and intergenerational trauma related to colonisation, dispossession and racism (Priest et al., 2011; Sherwood, 2013; Zubrick et al., 2006). The expression of mental health difficulties and what constitutes problematic behaviour also vary considerably between and among Indigenous and non-Indigenous cultures (Parker and Milroy, 2017). Assessing Aboriginal and Torres Strait Islander child mental health therefore requires cross-culturally valid measures that either account for other interrelated domains of SEWB or that can be used as part of a broader SEWB assessment.

Among numerous Australian national initiatives investigating the health of Aboriginal and Torres Strait Islander children (e.g. Longitudinal Study of Indigenous Children [LSIC], Study of Environment on Aboriginal Resilience and Child Health [SEARCH Investigators, 2010], South Australian Aboriginal Birth Cohort [SAABC; Jamieson et al., 2021], Western Australian Aboriginal Child Health Survey [WAACHS; Zubrick et al., 2004]), a combination of adapted measures and measures specifically developed for use in Aboriginal and Torres Strait populations has been used to assess mental health and wellbeing. One measure common across these studies is the Strengths and Difficulties Questionnaire (SDQ; Goodman et al., 1998), a 25-item screening questionnaire designed to assess emotional and behavioural strengths and problems in children aged 2–16 years.

The SDQ items are used to generate one strength subscale and four difficulty subscales, respectively: Prosocial Behaviour, Emotional Symptoms, Conduct Problems, Hyperactivity and Peer Problems. The difficulty subscales are further summarised by the higher-order Internalising Scale, Externalising Scale and a Total Difficulties Score (Goodman et al., 2010). The SDQ has three informant versions that can be completed independently by parents, teachers or children themselves once they reach 11 years of age. Originally normed in a large sample in the United Kingdom, numerous location-specific norms have since been developed to increase its applicability in a diverse range of populations, including within community samples of urban, predominantly non-Indigenous Australian children (e.g. Kremer et al., 2015; Mellor, 2005). Yet, a growing body of literature continues to identify flaws in its use in culturally diverse populations (e.g. Garrido et al., 2020) and specifically for Aboriginal and Torres Strait Islander children (Santiago et al., 2021).

Although qualitative findings to date generally support the acceptability of the SDQ’s use in urban Aboriginal communities (Williamson et al., 2010; Zubrick et al., 2006), concerns have been raised about the validity and reliability of some of the subscales. For instance, the Peer Problems subscale performed poorly on quantitative tests of reliability (α = 0.47; Williamson et al., 2014). Williamson et al. (2014) held focus groups and small group interviews with Aboriginal parents and Aboriginal Health Workers in urban communities and found that the subscale was poorly aligned with Aboriginal views of interpersonal relationships, as the scale did not consider a child’s interaction with their extended family or other community members. Similarly, behaviours measured by the Hyperactivity subscale were not always felt to be clinically significant but rather indicative of other issues, such as boredom or parenting difficulties. Importantly, the acceptability and interpretability of the SDQ items may vary geographically between different Aboriginal and Torres Strait Islander nations (Thurber et al., 2019; Williamson et al., 2010; Zubrick et al., 2006). The SDQ is felt to incompletely capture Aboriginal concepts of mental health, and it is not considered an acceptable proxy for SEWB (Marmor and Harley, 2018).

Finally, there is quantitative evidence to suggest that the hypothesised five-factor structure of the SDQ (Goodman, 2001) may not hold for the parent-reported questionnaire in the communities included in LSIC. Using principal component analysis (PCA) to analyse SDQ data from Wave 10 of LSIC, Thurber et al. (2019) found that a four-component solution explained the most variance for the parent-rated version. Items on the Peer Problems subscale either loaded onto the same component as items on the Prosocial subscale or the Emotional Symptoms subscale. More recently, Santiago et al. (2021) investigated the dimensionality of the parent-reported SDQ for Aboriginal and Torres Strait Islander children aged 4–10 years using data from the LSIC and SAABC studies. Neither factorial analyses nor results from exploratory graph analysis, which is a novel network psychometric technique, supported the construct validity of either the three-factor (i.e. Prosocial, Internalising and Externalising scales) or five-factor SDQ structures.

Using data from LSIC, this paper aims to build on these findings by: (a) investigating the factor structure of the teacher-reported SDQ, (b) extending the age range to cover children aged 2–15 years and (c) validating Santiago et al.’s (2021) results through alternative exploratory structural equation modelling techniques, including bifactor and hierarchical exploratory structural equation modelling (Asparouhov and Muthén, 2009). Data for the child self-reported version will not be presented in this paper as there were insufficient data available at the time of writing. Data were also not available for children aged 16 years in the current release.

## Method

### Research team

The research team consisted of one Aboriginal health scholar (C.M.-S.) with experience in qualitative health, Indigenous wellbeing and social science research and 12 non-Indigenous researchers with backgrounds in neurodevelopment (T.C., J.T., L.B., D.C., A.M., E.S., T.J.S., M.S.-S., N.S., D.P.S., M.A.B.), general child and youth mental health outcomes (D.P.S.) and cross-cultural psychology (L.J.). L.B. has lived experience of attention-deficit/hyperactivity disorder (ADHD) and D.P.S. has experience in child psychotherapy and assessment. C.M.-S. and L.J. provided the first, second and last authors with substantial guidance regarding the interpretation and presentation of the results in a culturally safe and appropriate manner. All authors were mindful of their cultural standpoint and other personal or professional viewpoints that may impact on their contribution to this paper, and of decentring their worldviews in the process of analysing the data and preparing the manuscript. This included attending training on Indigenous Research paradigms and cultural training (focusing on respect, reflection, communication, safety and quality and advocacy), practicing active listening and participating in discussions regarding the analyses and findings guided by C.M.-S.

### Ethical considerations

LSIC was approved by the Australian Government Department of Health Departmental Ethics Committee. Participating families provided written consent at recruitment and reconfirmed their consent at each wave of data collection. This study was approved by the Monash Human Research Ethics Committee (23720).

This study was designed in accordance with the objectives of the LSIC Steering Committee which consists of predominantly Aboriginal and Torres Strait Islander leaders who oversee the use of LSIC data to improve outcomes for Aboriginal and Torres Strait Islander children (Dodson et al., 2012). In particular, the objective we sought to contribute towards addressing was: ‘What helps Aboriginal and Torres Strait Islander children to stay on track or get them to become healthier, more positive and strong?’ While we adhered to the LSIC’s guidance to data users, no members of the LSIC governance and research groups were involved in this process.

The authors were cognisant of adhering to Indigenous ethical guidelines for research (Australian Institute of Aboriginal and Torres Strait Islander Studies [AIATSIS], 2020; National Health and Medical Research Council [NHMRC], 2018) at all stages during the preparation of this study. The NHMRC (2018) core values for ethical research with Aboriginal and Torres Strait Islander people are used as a framework to describe this below. Our use of LSIC data was guided by one of the four key objectives outlined by the LSIC Steering Committee (see above; *Respect, Reciprocity*) and necessitates full accountability to the LSIC Steering Committee and participating families and communities through the sharing of our research findings in a centralised repository for studies utilising LSIC data (*Responsibility*). The values of *Survival* and *Equality* were incorporated throughout our data analysis, interpretation and presentation stages by following the guidance of our Aboriginal co-author, reflecting on Aboriginal-led models of health and wellbeing (Gee et al., 2014) and findings from existing qualitative studies on the SDQ within Aboriginal communities. For example, this resulted in highlighting the importance of tailored, local supports as one way through which clinicians and researchers (particularly non-Indigenous colleagues) could appreciate the diversity and distinctiveness of all Aboriginal and Torres Strait Islander cultures. These actions, in addition to the consistent acknowledgement of differences between SEWB vs Western models of mental health, contributed towards upholding the value of *Spirit and Integrity* throughout this study.

### Study population

This study analysed data from Waves 2–11 (year 2009–2018) of LSIC (also known as *Footprints in Time*), the companion study to the Longitudinal Study of Australian Children (LSAC). Briefly, LSIC is managed by the Australian Government’s Department of Social Services as part of their Closing The Gap initiative (Department of Families, Housing, Community Services and Indigenous Affairs, 2009), and it aims to provide detailed insight to the development and wellbeing of approximately 1700 Aboriginal and Torres Strait Islander children. To maximise the utility and efficiency of the sample, an accelerated, cross-sequential cohort design was adopted. Study children belong to either the younger cohort (B Cohort, aged 6 months to 2 years at Wave 1) or the older cohort (K Cohort, aged 3.5–5 years at Wave 1). Further details regarding the LSIC study can be found in the Supplementary Materials (S1).

### Demographic variables

#### Sociodemographic variables

Information about the study child’s age, sex (male or female) and Indigenous status (Aboriginal, Torres Strait Islander or both), and the primary caregiver’s relationship to the child and Indigenous status were collected at the time of questionnaire completion. A randomised cluster variable corresponding to the participant’s nearest study site was also recorded to account for any sample clustering effects in subsequent modelling analyses (Hewitt, 2012). This variable corresponds to an Australian Bureau of Statistics (ABS) Indigenous Area based on the 2006 census.

#### Geographic variables

Two variables corresponding to the child’s level of community remoteness and relative isolation were collected at each wave. The Australian Statistical Geographical Classification (ASGC) Remoteness Areas classifies the remoteness of Australian cities into one of the five standardised categories (Major Cities of Australia, Inner Regional Australia, Outer Regional Australia, Remote Australia, Very Remote Australia) based on relative access to services (ABS, 2016). This represents an older version of the current Australian Statistical Geography Standard (ASGS) classification system that was adopted in 2011. In this paper, to ensure consistency, the older classification was used to characterise LSIC Waves 2–10 participant data as the newer classification was not available at the time of participant recruitment.

The Level of Relative Isolation (LORI) is a five-category indicator of geographic remoteness from services (No Isolation, Low Isolation, Moderate Isolation, High Isolation, Extreme Isolation) that was originally developed for the WAACHS Study in order to better describe the unique circumstances of many Aboriginal and Torres Strait Islander communities living in the most remote areas of Australia (Zubrick et al., 2004). LORI was designed to better account for differences in lifestyle, culture and language and health outcomes, as well as the accessibility of smaller-sized service centres, that are not captured by the ASGC Remoteness Area classifications. The two most remote classifications, ‘High’ and ‘Extreme’, are combined in the LSIC data due to small sample sizes.

### Materials

Two versions of the parent- and teacher-reported SDQ were given to informants depending on whether the participating child fell within the younger (2–4 years) or older (4–15 years) age bracket. Although most items are identical across both versions, the questionnaire for younger children rewords one item on the Hyperactivity scale and replaces two items on the Conduct Problems scale with more age-appropriate response options. Informants rate whether the behaviours described in each of the 25 items are ‘Not True’, ‘Somewhat True’ or ‘Certainly True’ for the child.

### Statistical analysis

Data were analysed using IBM’s SPSS Statistics version 27 and Mplus Version 8.7. Multivariate analyses of variance (MANOVAs) were used to establish whether there were significant effects of child age and sex on the distribution of subscale (Prosocial Behaviour, Emotional Symptoms, Conduct Problems, Hyperactivity, Peer Problems) and summary scale scores (Total Difficulties Score, Internalising Scale, Externalising Scale). Exploratory structural equation modelling (ESEM) with geomin rotation was conducted to investigate the factor structure of each of the parent-reported and teacher-reported versions of the SDQ for each age band. As the SDQ data were clustered by study site, we used type complex in Mplus for estimation as it accounts for this clustering effect by computing adjusted chi-square fit statistics (χ^2^) and adjusted standard errors using a sandwich estimator. Bifactor ESEM and higher-order exploratory structural equation modelling (H-ESEM) analyses were also conducted in Mplus to examine whether the three-factor and/or hierarchical (i.e. all items loading onto the Total Difficulties Score) models, respectively, were replicated in this population (see Van Zyl and ten Klooster, 2022 for Mplus syntax). Bifactor ESEM consists of a general factor on which all items load directly and one or more uncorrelated group factors that capture the residual variance with loadings from the items (Van Zyl and ten Klooster, 2022). If the direct loadings on the general factor are strong enough (e.g. explaining more than 80–90% of the modelled variance), it provides evidence for an overall total score providing a reasonable representation of the covariances in the data. H-ESEM consists of a second-order factor on which two or more first-order factors load (Van Zyl and ten Klooster, 2022). If the loadings of the first-order factors on the second-order factor are sufficiently strong explaining the majority of their variance, it again suggests an overall total score provides a reasonable representation of the item covariances. Internal consistency was estimated with McDonald’s omega (see Hayes and Coutts, 2020 for SPSS syntax) and interpreted with reference to Furr and Bacharach’s (2013) recommendations where scales were considered ‘acceptable’ for research use if internal consistency >0.7. A measure of test–retest reliability was not included as the time interval between questionnaire administration was in the order of months, which is well beyond the frequently recommended time frame of 2 weeks (Streiner et al., 2014).

## Results

### Participant demographics

Seven thousand and eight unique responses based on 1624 participating children were analysed. Demographic information is summarised in Table 1. Further detailed information about the sample characteristics, broken down by waves and age, can be found in the Supplementary Materials (S2.1–S2.6). As shown in Table 1, there were approximately equal numbers of male and female children. The majority of children were Aboriginal (87.1%), while a smaller proportion were Torres Strait Islanders (6.8%) or both (6.1%). Over half of participating families were living in major cities and inner regional areas and close to 80% of all families were living in locations classified as having low-to-no levels of relative isolation. The parent-reported SDQ was mainly completed by the child’s biological mother or father (89.4%), followed by their grandmother (either maternal or paternal; 5.9%), and informants were mostly Aboriginal, Torres Strait Islander or both (84.9%).

**Table 1.** Demographic details of participating children.

*N*	Frequency	Percentage (%)
	1624	100
Child’s Indigenous status		
Aboriginal	1415	87.1
Torres Strait Islander	110	6.8
Aboriginal and Torres Strait Islander	99	6.1
Child’s cohort		
B Cohort	923	56.8
K Cohort	700	43.1
Child’s sex		
Male	820	50.5
Female	804	49.5
Level of relative isolation		
None	41–372^a^	27.7^b^
Low	59–690^a^	51.3^b^
Moderate	12–190^a^	12.2^b^
High/extreme	8–143^a^	8.7^b^
Missing	0–4^a^	0.1^b^
ASGC remoteness area		
Major cities of Australia	41–372^a^	27.9^b^
Inner regional Australia	18–354^a^	27.6^b^
Outer regional Australia	16–203^a^	15.8^b^
Remote Australia	17–180^a^	10.9^b^
Very remote Australia	17–294^a^	17.8^b^
Missing	0–1^a^	0.01^b^
Parent-reported informant Indigenous status		
Aboriginal	81–1028^a^	74.1^b^
Torres Strait Islander	15–93^a^	7.0^b^
Aboriginal and Torres Strait Islander	3–50^a^	3.8^b^
Neither	21–236^a^	15.0^b^
Missing	0–3^a^	<0.01^b^
Parent-reported informant relationship to child		
Biological mother	111–1085^a^	82.1^b^
Biological father	2–324^a^	7.3^b^
Grandmother	5–84^a^	5.9^b^
Grandfather	0–5^a^	0.2^b^
Other	2–82^a^	4.5^b^

ASGC: Australian Statistical Geographical Classification.

a Range of frequencies across all waves.

b Average percentage taken across all waves.

#### Effects of age and sex

Table 2 displays MANOVA results investigating the effect of age and sex on SDQ scores for children aged 4–15 years. Small but significant multivariate effects of age (Pillai’s trace_*parent*_ = 0.05, *F* = 10.40, degree of freedom [df] = (17,140), *p* < 0.001; Pillai’s trace_*teacher*_ = 0.05, *F* = 7.16, df = (11,152), *p* < 0.001) and sex (Pillai’s trace_*parent*_ = 0.03, *F* = 25.41, df = (4282), *p* < 0.001; Pillai’s trace_*teacher*_ = 0.12, *F* = 75.36, df = (2785), *p* < 0.001), but not the interaction between age × sex (Pillai’s trace_*parent*_ = 0.005, *F* = 0.99, df = (17,140), *p* = 0.99; Pillai’s trace_*teacher*_ = 0.01, *F* = 1.56, df = (11,152), *p* = 0.052) were detected. Small effects of sex and age were observed on the distribution of all parent-reported subscale scores except for Emotional Symptoms and the Internalising Scale. Results also indicated small-to-medium effects of age and sex across all teacher-reported scores except for Emotional Symptoms. Across all ages, females tended to be rated higher on the Prosocial scale while males tended to be rated higher on all other subscales and summary scales. Interestingly, the teacher-reported Conduct Problems subscale demonstrated a significant age-by-sex interaction effect; however, the overall effect size was very small. Subsequent descriptive analyses were stratified by sex and age.

**Table 2.** MANOVA results for older children aged 4–15 years.

Scale	Parent report			Teacher report		
	*F*-value	*p*-value	Effect size ( $\eta_{p}^{2}$ )	*F*-value	*p*-value	Effect size ( $\eta_{p}^{2}$ )
Emotional symptoms						
Age	*F*(4, 4286) = 2.34	0.053	<0.01	*F*(4, 2789) = 10.76	<0.001	0.02
Sex	*F*(1, 4286) = 0.00	0.990	<0.01	*F*(1, 2789) = 0.534	0.465	<0.01
Interaction	*F*(4, 4286) = 0.86	0.489	<0.01	*F*(4, 2789) = 21.91	0.255	<0.01
Conduct problems						
Age	*F*(4, 4286) = 11.19	<0.001	0.01	*F*(4, 2789) = 6.51	<0.001	<0.01
Sex	*F*(1, 4286) = 12.41	<0.001	<0.01	*F*(1, 2789) = 112.02	<0.001	0.04
Interaction	*F*(4, 4286) = 1.30	0.268	<0.01	*F*(4, 2789) = 2.54	0.038	<0.01
Hyperactivity						
Age	*F*(4, 4286) = 11.15	<0.001	0.01	*F*(4, 2789) = 4.23	0.002	<0.01
Sex	*F*(1, 4286) = 84.53	<0.001	0.02	*F*(1, 2789) = 317.15	<0.001	0.10
Interaction	*F*(4, 4286) = 1.25	0.286	<0.01	*F*(4, 2789) = 0.92	0.449	<0.01
Peer problems						
Age	*F*(4, 4286) = 3.47	0.008	<0.01	*F*(4, 2789) = 4.32	0.002	<0.01
Sex	*F*(1, 4286) = 9.37	0.002	<0.01	*F*(1, 2789) = 11.06	0.001	<0.01
Interaction	*F*(4, 4286) = 0.52	0.720	<0.01	*F*(4, 2789) = 0.58	0.681	<0.01
Prosocial						
Age	*F*(4, 4286) = 11.31	<0.001	0.01	*F*(4, 2789) = 16.41	<0.001	0.02
Sex	*F*(1, 4286) = 57.28	<0.001	0.01	*F*(1, 2789) = 188.50	<0.001	0.06
Interaction	*F*(4, 4286) = 0.36	0.838	<0.01	*F*(4, 2789) = 0.67	0.610	<0.01
Internalising scale						
Age	*F*(4, 4286) = 3.25	0.011	<0.01	*F*(4, 2789) = 9.64	<0.001	0.01
Sex	*F*(1, 4286) = 2.68	0.102	<0.01	*F*(1, 2789) = 5.13	0.024	<0.01
Interaction	*F*(4, 4286) = 0.65	0.626	<0.01	*F*(4, 2789) = 0.65	0.628	<0.01
Externalising scale						
Age	*F*(4, 4286) = 14.52	<0.001	0.01	*F*(4, 2789) = 5.79	<0.001	<0.01
Sex	*F*(1, 4286) = 61.62	<0.001	0.01	*F*(1, 2789) = 262.60	<0.001	0.09
Interaction	*F*(4, 4286) = 1.19	0.314	<0.01	*F*(4, 2789) = 0.17	0.166	<0.01
Total difficulties score						
Age	*F*(4, 4286) = 4.20	0.002	<0.01	*F*(4, 2789) = 9.48	<0.001	0.01
Sex	*F*(1, 4286) = 33.89	<0.001	<0.01	*F*(1, 2789) = 147.01	<0.001	0.05
Interaction	*F*(4, 4286) = 0.69	0.598	<0.01	*F*(4, 2789) = 0.28	0.284	<0.01

MANOVA: multivariate analyses of variance.

### Descriptive statistics

Descriptive statistics for both parent-reported and teacher-reported SDQs can be found in the Supplementary Materials (S3.1–S3.12).

Table 3 displays the internal consistency of each scale stratified by age. The teacher-reported version of the SDQ responses demonstrated acceptable levels of internal consistency more often than the parent-reported version. Only the Total Difficulties Score was reliable for both parent- and teacher-reported versions across all ages. The Peer Problems scale performed poorly on tests of reliability in all groups except for the teacher-reported version of children aged 2–4 years. Interestingly, whereas the teacher-reported Prosocial scale was reliable across all age groups, the parent-reported equivalent was only acceptable for use for children between the ages of 10 and 14 years. However, it is also important to acknowledge that the complex factor structure of the SDQ in our sample may contribute to the observed pattern of poor internal consistency results.

**Table 3.** Omega coefficients (internal consistency) of the parent- and teacher-reported Strengths and Difficulties Questionnaire – combined sexes.

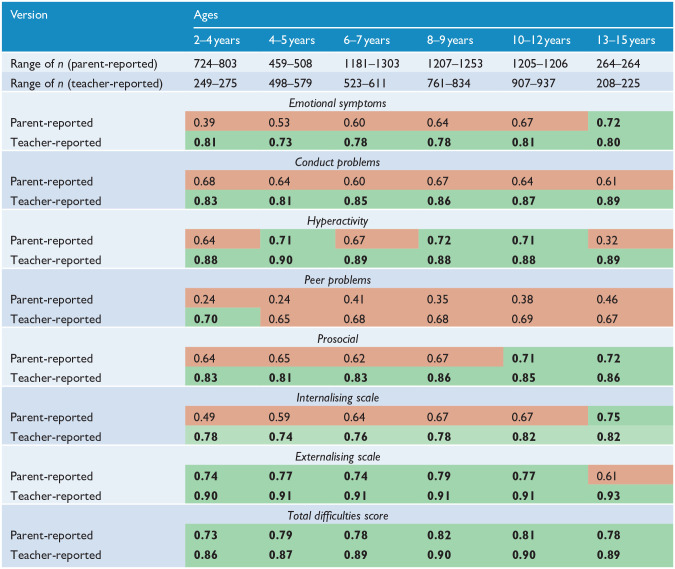

Green cells with bolded text indicate acceptable values (ω >0.70) and red cells indicate unacceptable values (ω < 0.70).

### Factor structure of the SDQ

#### Investigating the subscales

To extend upon work of previous authors (Santiago et al., 2021; Thurber et al., 2019), the fit of three-, four- and five-factor models were investigated. The results of these analyses are shown in Tables 4 and 5 for the parent- and teacher-reported SDQ, respectively. Across both informants and all age ranges, the goodness-of-fit indices suggested that a five-factor model demonstrated the best fit to the data (Bagozzi and Yi, 2012). The only exception to this was for the teacher-reported version for children aged 2–4 years where all models were misspecified and failed to converge on trustworthy estimates. However, a visual inspection of the factor loadings (see Supplementary Materials S4.1–S4.11 for the full-factor loadings for each model) indicated that the five-factor models did not consistently approximate the purported five-factor structure of the SDQ. In particular, the Peer Problems scale was not replicated across any of the age groups for the parent-reported questionnaire.

**Table 4.** ESEM goodness-of-fit indices for the parent-reported SDQ.

	Chi-square test of model fit	RMSEA [95% CI]	CFI	TLI	SRMR
Age 2–4 years					
Three factor	χ^2^(228, 753) = 439.56*	0.035 [0.030, 0.040]	0.913	0.886	0.052
Four factor	χ^2^(206, 753) = 322.47*	0.027 [0.021, 0.033]	0.952	0.930	0.041
**Five factor**	**χ**^2^ **(185, 753)** **=** 251.86*	**0.022 [0.015, 0.028]**	**0.973**	**0.955**	**0.035**
Age 4–5 years					
Three factor	χ^2^(228, 509) = 380.84*	0.036 [0.030, 0.043]	0.929	0.907	0.062
Four factor	χ^2^(206, 509) = 281.02*	0.027 [0.018, 0.034]	0.965	0.949	0.049
**Five factor**	**χ**^2^ **(185, 509)** **=** 231.64*	**0.022 [0.011, 0.031]**	**0.978**	**0.965**	**0.042**
Age 6–7 years					
Three factor	χ^2^(228, 1314) = 467.18*	0.028 [0.025, 0.032]	0.938	0.919	0.047
Four factor	χ^2^(205, 1314) = 358.53*	0.024 [0.020, 0.028]	0.961	0.943	0.039
**Five factor**	**χ**^2^ **(185, 1314)** **=** 279.95*	**0.020 [0.015, 0.024]**	**0.975**	**0.960**	**0.032**
Age 8–9 years					
Three factor	χ^2^(228, 1256) = 573.78*	0.035 [0.031, 0.038]	0.942	0.924	0.048
Four factor	χ^2^(206, 1256) = 395.50*	0.027 [0.023, 0.031]	0.968	0.954	0.036
**Five factor**	**χ**^2^ **(185, 1256)** **=** 331.18*	**0.025 [0.021, 0.029]**	**0.976**	**0.961**	**0.031**
Age 10–12 years					
Three factor	χ^2^(228, 1206) = 657.49*	0.040 [0.036, 0.043]	0.918	0.892	0.053
Four factor	χ^2^(206, 1206) = 469.29*	0.033 [0.029, 0.036]	0.950	0.927	0.040
**Five factor**	**χ**^2^ **(185, 1206)** **=** 361.30*	**0.028 [0.024, 0.032]**	**0.966**	**0.946**	**0.033**
Age 13–14 years					
Three factor	χ^2^(228, 263) = 322.02*	0.040 [0.029, 0.049]	0.941	0.922	0.062
Four factor	χ^2^(206, 263) = 469.29*	0.033 [0.029, 0.036]	0.950	0.927	0.040
**Five factor**	**χ**^2^ **(185, 263)** **=** 229.16*	**0.030 [0.014, 0.042]**	**0.972**	**0.955**	**0.047**

ESEM: exploratory structural equation modelling; SDQ: Strengths and Difficulties Questionnaire; RMSEA: root mean square error of approximation; CI: confidence interval; CFI: comparative fit index; TLI: Tucker–Lewis index; SRMR: standardised root mean square residual.

Bolded typeface = best fitting model.

* *p* < 0.001.

**Table 5.** ESEM goodness-of-fit indices for the teacher-reported SDQ.

	Chi-square test of model fit	RMSEA [95% CI]	CFI	TLI	SRMR
Age 4–5 years					
Three factor	χ^2^(228, 611) = 515.32*	0.045 [0.040, 0.051]	0.978	0.971	0.057
Four factor	χ^2^(206, 611) = 331.73*	0.032 [0.025, 0.038]	0.990	0.986	0.043
**Five factor**	**χ**^2^ **(185, 611)** **=** 255.07*	**0.025 [0.017, 0.032]**	**0.995**	**0.991**	**0.035**
Age 6–7 years					
Three factor	χ^2^(228, 621) = 551.88*	0.048 [0.043, 0.053]	0.974	0.966	0.059
Four factor	χ^2^(205, 621) = 334.80*	0.032 [0.025, 0.038]	0.990	0.985	0.038
**Five factor**	**χ**^2^ **(185, 621)** **=** 256.23*	**0.025 [0.017, 0.032]**	**0.994**	**0.991**	**0.030**
Age 8–9 years					
Three factor	χ^2^(228, 842) = 832.64*	0.056 [0.052, 0.060]	0.973	0.965	0.059
Four factor	χ^2^(206, 842) = 443.13*	0.037 [0.032, 0.042]	0.990	0.985	0.037
**Five factor**	**χ**^2^ **(185, 842)** **=** 320.59*	**0.030 [0.024, 0.035]**	**0.994**	**0.990**	**0.027**
Age 10–12 years					
Three factor	χ^2^(228, 949) = 904.92*	0.056 [0.052, 0.060]	0.971	0.962	0.049
Four factor	χ^2^(206, 949) = 494.81*	0.038 [0.034, 0.043]	0.988	0.982	0.033
**Five factor**	**χ**^2^ **(185, 949)** **=** 330.46*	**0.029 [0.024, 0.034]**	**0.994**	**0.990**	**0.024**
Age 13–15 years					
Three factor	χ^2^(228, 229) = 382.79*	0.054 [0.045, 0.064]	0.978	0.971	0.063
Four factor	χ^2^(206, 229) = 290.01*	0.042 [0.030, 0.053]	0.988	0.875	0.050
**Five factor**	**χ**^2^ **(185, 229)** **=** 211.51*	**0.025 [0.000, 0.040]**	**0.996**	**0.994**	**0.035**

ESEM: exploratory structural equation modelling; SDQ: Strengths and Difficulties Questionnaire; RMSEA: root mean square error of approximation; CI: confidence interval; CFI: comparative fit index; TLI: Tucker–Lewis index; SRMR: standardised root mean square residual.

Bolded typeface = best fitting model.

* *p* < 0.001.

#### Investigating the summary scales

An alternative structure of the SDQ includes the Prosocial subscale and the Internalising and Externalising summary scales (i.e. a three-factor structure; Goodman et al., 2010). However, as demonstrated in Tables 4 and 5, a three-factor model did not demonstrate the best model fit for either informant. Subsequent analyses to explore this structure were therefore not completed.

The final possible characterisation of SDQ responses involves summarising the four difficulties subscales into the Total Difficulties Score. Before commencing these analyses, ESEM analyses were completed on the difficulties items. The results are displayed in Tables 6 and 7. Goodness-of-fit statistics indicated that while a four-factor model often demonstrated the best fit, the theoretical structure of the SDQ was, once again, not consistently supported (see Supplementary Materials S5.1–S5.12). Two hierarchical ESEM configurations of the difficulties items were subsequently considered: (a) a bifactor ESEM model and (b) an H-ESEM model.

**Table 6.** ESEM goodness-of-fit indices for the difficulties items of the parent-reported SDQ.

	Chi-square test of model fit	RMSEA [95% CI]	CFI	TLI	SRMR
Age 2–4 years					
Two factor	χ^2^(151, 753) = 447.76*	0.051 [0.046, 0.057]	0.839	0.798	0.074
Three factor	χ^2^(133, 753) = 297.64*	0.041 [0.034, 0.047]	0.911	0.873	0.051
**Four factor**	**χ**^2^ **(116, 753)** **=** 191.83*	**0.029 [0.022, 0.037]**	**0.959**	**0.933**	**0.038**
Age 4–5 years					
Two factor	χ^2^(151, 509) = 393.67*	0.056 [0.049, 0.063]	0.863	0.827	0.079
Three factor	χ^2^(133, 509) = 287.52*	0.048 [0.040, 0.055]	0.913	0.875	0.064
**Four factor**	**χ**^2^ **(116, 509)** **=** 176.79*	**0.032 [0.022, 0.041]**	**0.966**	**0.944**	**0.044**
Age 6–7 years					
Two factor	χ^2^(151, 1314) = 520.92*	0.043 [0.039, 0.047]	0.885	0.855	0.063
Three factor	χ^2^(133, 1314) = 349.62*	0.035 [0.031, 0.040]	0.933	0.904	0.048
**Four factor**	**χ**^2^ **(116, 1314)** **=** 234.23*	**0.028 [0.023, 0.033]**	**0.963**	**0.940**	**0.036**
Age 8–9 years					
Two factor	χ^2^(151, 1256) = 687.71*	0.053 [0.049, 0.057]	0.896	0.869	0.066
Three factor	χ^2^(133, 1256) = 468.51*	0.045 [0.040, 0.049]	0.935	0.907	0.050
**Four factor**	**χ**^2^ **(116, 1256)** **=** 267.52*	**0.032 [0.027, 0.037]**	**0.971**	**0.952**	**0.034**
Age 10–12 years					
Two factor	χ^2^(151, 1206) = 735.28*	0.057 [0.053, 0.061]	0.859	0.823	0.069
Three factor	χ^2^(133, 1206) = 501.17*	0.048 [0.043, 0.052]	0.911	0.873	0.056
**Four factor**	**χ**^2^ **(116, 1206)** **=** 335.18*	**0.040 [0.035, 0.045]**	**0.947**	**0.913**	**0.039**
Age 13–14 years					
Two factor	χ^2^(151, 264) = 279.24*	0.057 [0.046, 0.067]	0.893	0.866	0.077
Three factor	χ^2^(133, 264) = 208.77*	0.047 [0.034, 0.058]	0.937	0.910	0.060
**Four factor**	**χ**^2^ **(116, 264)** **=** 166.95*	**0.041 [0.026, 0.054]**	**0.958**	**0.930**	**0.051**

ESEM: exploratory structural equation modelling; SDQ: Strengths and Difficulties Questionnaire; RMSEA: root mean square error of approximation; CI: confidence interval; CFI: comparative fit index; TLI: Tucker–Lewis index; SRMR: standardised root mean square residual.

Bolded typeface = best fitting model.

* *p* < 0.001.

**Table 7.** ESEM goodness-of-fit indices for the difficulties items of the teacher-reported SDQ.

	Chi-square test of Model fit	RMSEA [95% CI]	CFI	TLI	SRMR
Age 2–4 years					
**Two factor**	**χ**^2^ **(151, 272)** **=** 361.69*	**0.072 [0.062, 0.081]**	**0.945**	**0.931**	**0.114**
Three factor^a^					
Four factor^a^					
Age 4–5 years					
Two factor	χ^2^(151, 611) = 451.42*	0.057 [0.051, 0.063]	0.972	0.965	0.069
Three factor	χ^2^(133, 611) = 310.17*	0.047 [0.040, 0.053]	0.984	0.977	0.056
**Four factor**	**χ**^2^ **(116, 611)** **=** 208.73*	**0.036 [0.028, 0.044]**	**0.991**	**0.986**	**0.043**
Age 6–7 years					
Two factor	χ^2^(151, 621) = 534.52*	0.064 [0.058, 0.070]	0.961	0.951	0.068
Three factor	χ^2^(133, 621) = 284.01*	0.043 [0.036, 0.050]	0.985	0.978	0.046
**Four factor**	**χ**^2^ **(116, 621)** **=** 195.31*	**0.033 [0.025, 0.041]**	**0.992**	**0.987**	**0.034**
Age 8–9 years					
Two factor	χ^2^(151, 842) = 830.50*	0.073 [0.068, 0.078]	0.959	0.949	0.078
Three factor	χ^2^(133, 842) = 516.73*	0.059 [0.053, 0.064]	0.977	0.967	0.051
**Four factor**	**χ**^2^ **(116, 842)** **=** 264.69*	**0.039 [0.033, 0.045]**	**0.991**	**0.985**	**0.033**
Age 10–12 years					
Two factor	χ^2^(151, 1206) = 902.58*	0.072 [0.068, 0.077]	0.956	0.944	0.063
Three factor	χ^2^(133, 1206) = 533.72*	0.056 [0.051, 0.061]	0.976	0.966	0.042
**Four factor**	**χ**^2^ **(116, 1206)** **=** 333.42*	**0.044 [0.039, 0.050]**	**0.987**	**0.979**	**0.033**
Age 13–15 years					
Two factor	χ^2^(151, 229) = 364.73*	0.079 [0.068, 0.089]	0.965	0.956	0.086
Three factor	χ^2^(133, 229) = 256.38*	0.064 [0.052, 0.075]	0.980	0.971	0.065
**Four factor**	**χ**^2^ **(116, 229)** **=** 187.34*	**0.052 [0.038, 0.065]**	**0.988**	**0.981**	**0.047**

ESEM: exploratory structural equation modelling; SDQ: Strengths and Difficulties Questionnaire; RMSEA: root mean square error of approximation; CI: confidence interval; CFI: comparative fit index; TLI: Tucker–Lewis index; SRMR: standardised root mean square residual.

Bolded typeface = best fitting model.

* *p* < 0.001.

##### Bifactor ESEM

Results from Tables 8 and 9 suggested that good model fit was demonstrated in most age groups across both informants. However, visual inspection of the factor loadings indicated that all items did not consistently load onto the general factor (see Supplementary Materials S6.1–S6.11). This is congruent with results from the prior ESEM analysis where the structural validity of the difficulties subscales was not supported.

**Table 8.** Bifactor ESEM goodness-of-fit indices for the parent-reported SDQ difficulties items.

	Chi-square test of model fit	RMSEA [95% CI]	CFI	TLI	SRMR
Age 2–4 years	χ^2^(100, 753) = 144.31**	0.024 [0.015, 0.033]	0.976	0.954	0.032
Age 4–5 years	χ^2^(100, 509) = 129.15**	0.024 [0.009, 0.035]	0.984	0.969	0.035
Age 6–7 years	χ^2^(100, 1314) = 159.16**	0.021 [0.015, 0.027]	0.982	0.965	0.026
Age 8–9 years	χ^2^(100, 1256) = 213.95**	0.030 [0.025, 0.036]	0.978	0.958	0.029
Age 10–12 years	χ^2^(100, 1206) = 225.07**	0.032 [0.027, 0.038]	0.970	0.943	0.030
Age 13–14 years	χ^2^(100, 263) = 128.62*	0.033 [0.011, 0.049]	0.976	0.955	0.042

ESEM: exploratory structural equation modelling; SDQ: Strengths and Difficulties Questionnaire; RMSEA: root mean square error of approximation; CI: confidence interval; CFI: comparative fit index; TLI: Tucker–Lewis index; SRMR: standardised root mean square residual.

* *p* < 0.05; **p < 0.001.

**Table 9.** Bifactor ESEM goodness-of-fit indices for the teacher-reported SDQ difficulties items.

	Chi-square test of model fit	RMSEA [95% CI]	CFI	TLI	SRMR
Age 2–4 years	*Not positive definite*				
Age 4–5 years	χ^2^(100, 611) = 154.32**	0.030 [0.020, 0.039]	0.995	0.991	0.034
Age 6–7 years	χ^2^(100, 621) = 143.87**	0.027 [0.016, 0.036]	0.996	0.991	0.029
Age 8–9 years	χ^2^(100, 842) = 161.12**	0.027 [0.019, 0.034]	0.996	0.993	0.024
Age 10–12 years	χ^2^(100, 949) = 202.01**	0.033 [0.026, 0.039]	0.994	0.989	0.023
Age 13–15 years	χ^2^(100, 229) = 124.37*	0.033 [0.001, 0.050]	0.996	0.992	0.033

ESEM: exploratory structural equation modelling; SDQ: Strengths and Difficulties Questionnaire; RMSEA: root mean square error of approximation; CI: confidence interval; CFI: comparative fit index; TLI: Tucker–Lewis index; SRMR: standardised root mean square residual.

* *p* < 0.05; **p < 0.001.

##### H-ESEM

Model fit results are displayed in Tables 10 and 11. Most models were either not positive definite or misspecified, and therefore did not converge on trustworthy estimates. Results from Tables 12 and 13 indicate there was no evidence to support the existence of a general ‘difficulties’ factor in any age group for either informant as none of the remaining models demonstrated consistently strong standardised factor loadings onto the general factor.

**Table 10.** H-ESEM results for the parent-reported SDQ difficulties items.

Age (years)	Log-likelihood (H0)	AIC	BIC	Chi-square test of model fit	RMSEA [95% CI]	CFI	TLI	SRMR
2–4	−14,670.38	29,564.77	30,082.67	χ^2^(118, 753 = 240.39**	0.037 [0.030, 0.044]	0.937	0.899	0.029
4–5^a^								
6–7^a^								
8–9	−22,940.18	46,104.35	46,679.55	χ^2^(118, 1256 = 373.76**	0.042 [0.037, 0.046]	0.941	0.906	0.026
10–12	−21,220.65	42,665.30	43,235.95	χ^2^(118, 1206 = 491.62**	0.051 [0.047, 0.056]	0.919	0.869	0.030
13–14^a^								

H-ESEM: higher-order exploratory structural equation modelling; SDQ: Strengths and Difficulties Questionnaire; AIC: Akaike information criterion; BIC: Bayesian information criterion; RMSEA: root mean square error of approximation; CI: confidence interval; CFI: comparative fit index; TLI: Tucker–Lewis index; SRMR: standardised root mean square residual.

a Model failed to converge.

** *p* < 0.001.

**Table 11.** H-ESEM results for the teacher-reported SDQ difficulties items.

Age (years)	Log-likelihood (H0)	AIC	BIC	Chi-square test of model fit	RMSEA [95% CI]	CFI	TLI	SRMR
2–4^a^								
4–5	−7971.814	16,167.63	16,662.12	χ^2^(118, 611 = 477.64**	0.071 [0.064, 0.077]	0.921	0.873	0.037
6–7^a^								
8–9	−11,882.277	23,988.56	24,518.96	χ^2^(118, 842 = 565.14**	0.067 [0.062, 0.073]	0.939	0.902	0.035
10–12^a^								
13–15^b^								

H-ESEM: higher-order exploratory structural equation modelling; SDQ: Strengths and Difficulties Questionnaire; AIC: Akaike information criterion; BIC: Bayesian information criterion; RMSEA: root mean square error of approximation; CI: confidence interval; CFI: comparative fit index; TLI: Tucker–Lewis index; SRMR: standardised root mean square residual.

a Not positive definite matrix indicating model misspecification.

b Model failed to converge.

** *p* < 0.001.

**Table 12.** H-ESEM loadings onto higher-order factor and *R*^2^ – parent-reported SDQ.

Factor	Standardised factor loading	Standard error	*R*^2^-value
2–4 years			
F1	0.506	0.159	0.256
F2	0.545	0.146	0.297
F3	0.778	0.195	0.605
F4	−0.543	0.273	0.295
8–9 years			
F1	0.544	0.119	0.296
F2	0.527	0.134	0.278
F3	0.667	0.213	0.445
F4	0.692	0.274	0.479
10–12 years			
F1	0.667	0.293	0.445
F2	0.499	0.184	0.249
F3	0.255	0.323	0.065
F4	0.548	0.373	0.301

H-ESEM: higher-order exploratory structural equation modelling; SDQ: Strengths and Difficulties Questionnaire.

**Table 13.** H-ESEM loadings onto higher-order factor and *R*^2^ – teacher-reported SDQ.

Factor	Standardised factor loading	Standard error	*R*^2^-value
4–5 years			
F1	0.869	0.158	0.756
F2	0.580	0.096	0.336
F3	0.370	0.119	0.137
F4	0.318	0.123	0.101
8–9 years			
F1	0.818	0.150	0.668
F2	0.034	0.146	0.001
F3	0.383	0.082	0.146
F4	0.928	0.210	0.862

H-ESEM: higher-order exploratory structural equation modelling; SDQ: Strengths and Difficulties Questionnaire.

## Discussion

The SDQ is a widely used questionnaire that screens for emotional and behavioural strengths and difficulties in children. Past studies have shown that while the SDQ demonstrates adequate acceptability for use with Aboriginal and Torres Strait Islander communities (e.g. Williamson et al., 2010), quantitative analyses of its factor structure indicate poor validity that preclude the interpretation of its subscale and summary scale scores (Santiago et al., 2021). Here, we extend upon the findings of Santiago et al. (2021) and present detailed analyses of the psychometric properties of the parent- and teacher-reported versions of the SDQ for Aboriginal and Torres Strait Islander children aged 2–15 years participating in LSIC. Responses from teachers demonstrated more robust internal consistency across the age groups than responses from parents. Although a five-factor model demonstrated the best quantitative fit for the parent- and teacher-reported SDQ, the models failed to consistently replicate the questionnaire’s purported five-factor structure (Goodman, 2001). Bifactor and hierarchical ESEM models also failed to support the use of the Total Difficulties Score. This emphasises the complex factor structure of the SDQ, particularly within Aboriginal and Torres Strait Islander communities. Additionally, the findings highlight the invalidity of interpreting the individual subscales.

This study replicated Williamson et al.’s (2014) finding that, for the parent report, only the Total Difficulties Score displayed acceptable levels of reliability across all age groups, whereas the Peer Problems scale failed to demonstrate adequate internal consistency in any group. A similar pattern was also demonstrated here for the teacher-reported SDQ, which is the first, to our knowledge, investigation of the reliability of the teacher-reported SDQ in Aboriginal and Torres Strait Islander children. This study also extended Thurber et al.’s (2019) investigation of the internal consistency of parent- vs teacher-reported SDQ in Wave 10 of LSIC and generalised their observed discrepancy across all waves of data and in a combined cohort. Thurber and colleagues proposed that the differences may be attributable to cultural factors (e.g. Indigenous status of teachers and parents), variations in the presentation of emotional and behavioural difficulties across settings (i.e. at home vs the classroom) and challenges associated with translating the SDQ into local Aboriginal and Torres Strait Islander languages (see also Zubrick et al., 2006). These differences are also necessarily underscored by the lack of structural validity of the SDQ. Taken together, our findings demonstrate the importance of considering a psychological assessment tool’s reliability and validity in tandem, within the broader cultural conceptualisation of mental health.

The large, diverse sample that included contributions from numerous Aboriginal and Torres Strait Islander communities is a key strength of this study. Presenting the data separated by age and sex allowed us to account for varying cultural expectations of children’s emotional and behavioural development in the different groups. However, it is important to acknowledge that this may not necessarily apply to all families. For example, in one remote Aboriginal community, researchers found that families encouraged children to develop their social and emotional maturity regardless of their age (Byers et al., 2012). This suggests that age-related normative indicators of development may be less useful than a milestone-based system in certain communities, and may partly explain why we found no statistically significant effect of age or sex on the parent-reported Emotional Symptoms subscale. Indeed, there remains an ongoing need for responsive care that is appropriately tailored to local cultures.

There are several limitations to this study. First, the authorship team learnt of more appropriate ways of conducting research focusing on Aboriginal and Torres Strait Islander communities and engaging with the LSIC data after the study was conceived and significantly underway. Moving forward, it is important that studies developing and examining measures of SEWB for Aboriginal youth be (a) Aboriginal and Torres Strait Islander led and (b) collaborate and empower community as per the relevant ethical guidelines and data sovereignty and governance principles (AIATSIS, 2020). Additionally, this study would be improved by including a wider group of Aboriginal and Torres Strait Islander researchers and community members that have expertise and/or interest in different areas of this study and at all stages from conception to dissemination of findings.

Second, we were unable to undertake any external validation of our findings with participating LSIC families and teachers to see whether the results aligned with their experience of the questionnaire. Qualitative interviews could provide valuable insights into how to improve the use and interpretation of measurement tools in LSIC. Indeed, it is possible that an alternative configuration of the SDQ subscales and summary scales – or even the removal of particular items – could have better captured their perspective of the child’s emotional and behavioural strengths and demonstrated more robust statistical qualities.

Third, we were unable to explicitly account for geographic variation in the data (i.e. separating results by urban, regional and remote areas) due to unbalanced sample sizes in each geographic group across the age bands. The analysis and interpretation of the data therefore may not fully account for variations in child rearing practices that are unique to communities based in different regions. Moreover, mobility, whether temporary or more permanent, facilitates the maintenance of kinship relationships and connections to the land and country for many Aboriginal and Torres Strait Islander people (Dockery and Colquhoun, 2012) and is essential to wellbeing (Taylor-Bragge et al., 2021). As such, even if the data were stratified by geographic location, it may still incompletely capture the unique contributions of the connection to land and country on a child’s social and emotional development.

Fourth, we did not account for the Indigenous status of participating teachers. Although the overall percentage of Aboriginal and Torres Strait Islander teachers within LSIC is low (Thurber et al., 2019), it is important to consider the impact of the teacher’s cultural background on their classroom expectations, pedagogy and interpretation of child behaviour. For example, teachers that adopt bicultural teaching strategies, such as having a bilingual classroom or incorporating tasks adapted to Aboriginal learning styles (Yunkaporta, 2010; Yunkaporta and McGinty, 2009), may encourage Aboriginal and Torres Strait Islander students to showcase different personal strengths than those in classrooms without culturally responsive features. Variations in these factors may also explain, in part, the observed differences in internal consistency between the parent- and teacher-reported SDQ. Additionally, it is interesting to note that the reliability of the teacher-reported responses was often high despite the questionnaire demonstrating unacceptably low validity. This may also point to broader, foundational issues with teacher-reported data in LSIC as the responses may be capturing Aboriginal and Torres Strait Islander children’s function through a predominantly non-Indigenous lens.

Finally, it was beyond the scope of this paper to formally assess the underlying reasons why the SDQ’s structural validity was not upheld in this population. For example, it may be worth investigating which SDQ items differed systematically between different Aboriginal and Torres Strait Islander communities (i.e. measurement non-invariance or differential item functioning; Holland and Thayer, 1988). Indeed, qualitative studies of the acceptability of the SDQ in some discrete Aboriginal communities has shown that items on the Hyperactivity and Peer Problems scales were not always felt to accurately reflect the meanings behind a child’s behaviour (Williamson et al., 2010, 2014), but what constitutes an accurate reflection may still vary considerably between different First Nations cultures and generations. Future studies could evaluate the measurement non-invariance or differential item functioning of the SDQ among Aboriginal and Torres Strait Islander informants to provide insight on how we can better tailor the questionnaire for use within different Aboriginal and Torres Strait Islander communities. Should this be undertaken with a larger number of assessment tools, it may eventually allow for the comparison of children’s strengths and challenges to facilitate the national development of targeted SEWB supports for each region.

### Recommendations for clinicians and researchers

As the questionnaire demonstrated poor structural validity, clinicians and researchers are urged to reconsider their use of the SDQ scale scores when working with Aboriginal and Torres Strait Islander children. It may still be helpful to consider responses at the item level as part of a holistic understanding of the child’s SEWB or as a launching point for further assessment. For example, responses to the item ‘often volunteers to help others’ could be used to initiate a conversation around the child’s values and responsibilities within their community with reference to age- and sex-related cultural norms (if applicable). Such use should be complemented by the inclusion of other validated measures of mental strength and resilience that look beyond prosocial skills (e.g. Strong Souls; Thomas et al., 2010), as it has been shown that a connection to other wellbeing domains, such as culture (Murrup-Stewart et al., 2021), are deeply important to the wellbeing of Aboriginal and Torres Strait Islander youth. It is essential that clinicians and researchers consider the impact of their own cultures, perspectives and biases on their description and interpretation of the item responses. While working towards decolonising one’s own psychological research and practice is a highly personal endeavour, we recommend ‘Working Together: Aboriginal and Torres Strait Islander Mental Health and Wellbeing Principles and Practice’ (Gee et al., 2014) as one crucial resource for all clinicians and researchers within this space. Engagement with organisations such as the Australian Indigenous Psychologists Association (AIPA) is also highly valuable.

Importantly, and as outlined in previous studies (e.g. Marmor and Harley, 2018; Thurber et al., 2019), the health of an Aboriginal or Torres Strait Islander child’s mind and emotions forms only one component of their overall SEWB (Gee et al., 2014). It will be important for researchers and clinicians to also seek to understand the other cultural, spiritual, social and physical domains of SEWB when working with Aboriginal and Torres Strait Islander children presenting with health and wellbeing concerns. As such, collaborations with Aboriginal and Torres Strait Islander researchers, clinicians and community members remain critical to ensure a culturally safe foundation of care. There is a need for future studies led by Aboriginal and Torres Strait Islander experts to develop screening and assessment scales that encompass additional markers of personal strengths and differences, and that consider the sociocultural determinants of health relevant to Aboriginal and Torres Strait islander populations. Additionally, when developing these screening and assessment scales, researchers need to consider the use of language, such as the use of neutral language and strength-based discourse (vs deficit-focused discourse) to describe and classify markers of personal differences. That is, language that does not infer negative judgement or elicit negative narratives or stereotypes, such as the word ‘problem’, or classify children as ‘abnormal’. A participatory action approach (e.g. Dudgeon et al., 2020a) led by members from Indigenous communities and the neurodevelopment sphere that utilises the unique and intersectional knowledge, strengths and lessons from action within these communities is essential for cultural acceptability and effective change.

## Conclusion

This paper presents detailed analyses of the parent- and teacher-reported SDQ for a large sample of Aboriginal and Torres Strait islander children aged 2–15 years. The findings indicate that the screening tool has poor structural validity and inconsistent reliability for primary caregivers and teachers across all age groups. To better recognise and support the growth of strong and resilient Aboriginal and Torres Strait Islander children, researchers and clinicians are encouraged to use alternative, culturally appropriate measures of mental health and wellbeing as part of a broader assessment of a child’s SEWB.

## Supplemental Material

sj-docx-1-anp-10.1177_00048674231161504 – Supplemental material for Against the use of the Strengths and Difficulties Questionnaire for Aboriginal and Torres Strait Islander children aged 2–15 years Click here for additional data file. Supplemental material, sj-docx-1-anp-10.1177_00048674231161504 for Against the use of the Strengths and Difficulties Questionnaire for Aboriginal and Torres Strait Islander children aged 2–15 years by Tracey Chau, Jeggan Tiego, Louise Brown, David Coghill, Laura Jobson, Alicia Montgomery, Cammi Murrup-Stewart, Emma Sciberras, Tim J Silk, Megan Spencer-Smith, Nicole Stefanac, Daniel P Sullivan and Mark A Bellgrove in Australian & New Zealand Journal of Psychiatry

sj-docx-2-anp-10.1177_00048674231161504 – Supplemental material for Against the use of the Strengths and Difficulties Questionnaire for Aboriginal and Torres Strait Islander children aged 2–15 years Click here for additional data file. Supplemental material, sj-docx-2-anp-10.1177_00048674231161504 for Against the use of the Strengths and Difficulties Questionnaire for Aboriginal and Torres Strait Islander children aged 2–15 years by Tracey Chau, Jeggan Tiego, Louise Brown, David Coghill, Laura Jobson, Alicia Montgomery, Cammi Murrup-Stewart, Emma Sciberras, Tim J Silk, Megan Spencer-Smith, Nicole Stefanac, Daniel P Sullivan and Mark A Bellgrove in Australian & New Zealand Journal of Psychiatry

sj-docx-3-anp-10.1177_00048674231161504 – Supplemental material for Against the use of the Strengths and Difficulties Questionnaire for Aboriginal and Torres Strait Islander children aged 2–15 years Click here for additional data file. Supplemental material, sj-docx-3-anp-10.1177_00048674231161504 for Against the use of the Strengths and Difficulties Questionnaire for Aboriginal and Torres Strait Islander children aged 2–15 years by Tracey Chau, Jeggan Tiego, Louise Brown, David Coghill, Laura Jobson, Alicia Montgomery, Cammi Murrup-Stewart, Emma Sciberras, Tim J Silk, Megan Spencer-Smith, Nicole Stefanac, Daniel P Sullivan and Mark A Bellgrove in Australian & New Zealand Journal of Psychiatry

sj-docx-4-anp-10.1177_00048674231161504 – Supplemental material for Against the use of the Strengths and Difficulties Questionnaire for Aboriginal and Torres Strait Islander children aged 2–15 years Click here for additional data file. Supplemental material, sj-docx-4-anp-10.1177_00048674231161504 for Against the use of the Strengths and Difficulties Questionnaire for Aboriginal and Torres Strait Islander children aged 2–15 years by Tracey Chau, Jeggan Tiego, Louise Brown, David Coghill, Laura Jobson, Alicia Montgomery, Cammi Murrup-Stewart, Emma Sciberras, Tim J Silk, Megan Spencer-Smith, Nicole Stefanac, Daniel P Sullivan and Mark A Bellgrove in Australian & New Zealand Journal of Psychiatry

sj-docx-5-anp-10.1177_00048674231161504 – Supplemental material for Against the use of the Strengths and Difficulties Questionnaire for Aboriginal and Torres Strait Islander children aged 2–15 years Click here for additional data file. Supplemental material, sj-docx-5-anp-10.1177_00048674231161504 for Against the use of the Strengths and Difficulties Questionnaire for Aboriginal and Torres Strait Islander children aged 2–15 years by Tracey Chau, Jeggan Tiego, Louise Brown, David Coghill, Laura Jobson, Alicia Montgomery, Cammi Murrup-Stewart, Emma Sciberras, Tim J Silk, Megan Spencer-Smith, Nicole Stefanac, Daniel P Sullivan and Mark A Bellgrove in Australian & New Zealand Journal of Psychiatry

sj-docx-6-anp-10.1177_00048674231161504 – Supplemental material for Against the use of the Strengths and Difficulties Questionnaire for Aboriginal and Torres Strait Islander children aged 2–15 years Click here for additional data file. Supplemental material, sj-docx-6-anp-10.1177_00048674231161504 for Against the use of the Strengths and Difficulties Questionnaire for Aboriginal and Torres Strait Islander children aged 2–15 years by Tracey Chau, Jeggan Tiego, Louise Brown, David Coghill, Laura Jobson, Alicia Montgomery, Cammi Murrup-Stewart, Emma Sciberras, Tim J Silk, Megan Spencer-Smith, Nicole Stefanac, Daniel P Sullivan and Mark A Bellgrove in Australian & New Zealand Journal of Psychiatry
