# Supplementary material for: Against the use of the Strengths and Difficulties Questionnaire for Aboriginal and Torres Strait Islander children aged 2–15 years
Source: Aust N Z J Psychiatry. 2023 Mar 28;57(10):1343–58. doi: 10.1177/00048674231161504 (PMC10517593; doi:10.1177/00048674231161504)
Supplement: sj-docx-1-anp-10.1177_00048674231161504 – Supplemental material for Against the use of the Strengths and Difficulties Questionnaire for Aboriginal and Torres Strait Islander children aged 2–15 years [file sj-docx-1-anp-10.1177_00048674231161504.docx]

S1.

*Supplementary information regarding the LSIC study population*

LSIC is an ongoing study that aims to identify the unique needs of Aboriginal and Torres Strait Islander children to inform policy and program development so that they can be supported to grow up healthy and strong. Potential respondents were initially recruited using a non-random purposive sampling design so that there were approximately equivalent numbers of children from urban, regional, and remote areas to allow for geographical comparisons (Dodson et al., 2012). This means that the sample cannot be considered as representative of all Aboriginal and Torres Strait Islander children, as the population spread between remote and urban regions is estimated to range from 6.7% (Remote) to 37.4% (Major Cities; Australian Bureau of Statistics [ABS], 2016). In light of the study design as described in the main article, most participants contributed more than one set of parent- and teacher-reported data across each age band. All participants provided written informed consent.

Data collection is conducted annually through face-to-face interviews facilitated by Indigenous Research Administration Officers (RAOs) across 11 different study sites. The use of an interview format rather than the traditional paper-based administration of the questionnaire is important as the use of questionnaires is felt to be culturally inappropriate (Williamson et al., 2010). Informants for the parent-reported SDQs were typically the study child’s primary caregiver and the questionnaire was completed as part of the annual family interview with an RAO in Waves 3, 4, 6, 8, and 10. The teacher-reported SDQs were commonly completed by the child’s classroom teacher or childcare worker, with the option of completing the questionnaire with or without the assistance of an RAO, in Waves 2-6 and 9-11.
