# Supplementary material for: Against the use of the Strengths and Difficulties Questionnaire for Aboriginal and Torres Strait Islander children aged 2–15 years
Source: Aust N Z J Psychiatry. 2023 Mar 28;57(10):1343–58. doi: 10.1177/00048674231161504 (PMC10517593; doi:10.1177/00048674231161504)
Supplement: sj-docx-2-anp-10.1177_00048674231161504 – Supplemental material for Against the use of the Strengths and Difficulties Questionnaire for Aboriginal and Torres Strait Islander children aged 2–15 years [file sj-docx-2-anp-10.1177_00048674231161504.docx]

S2.1

*Demographic Details for Participating Children Aged 2-4 Years*

|  | Wave 3 | Wave 4 | Total | Percentage |
| --- | --- | --- | --- | --- |
| Sample size | 811 | 199 | 1010 | 100% |
| Sex |  |  |  |  |
| Male | 411 | 109 | 520 | 51% |
| Female | 400 | 90 | 490 | 49% |
| Indigenous status |  |  |  |  |
| Aboriginal | 704 | 181 | 885 | 87.62% |
| Torres Strait Islander | 57 | 6 | 63 | 6.24% |
| Both | 50 | 12 | 62 | 6.14% |
| Level of Relative Isolation (2016 for Wave 11) |  |  |  |  |
| None | 224 | 70 | 294 | 29.11% |
| Low | 393 | 106 | 499 | 49.41% |
| Moderate | 121 | 17 | 138 | 13.66% |
| High/Extreme | 73 | 6 | 79 | 7.82% |
| Missing | 0 | 0 | 0 | 0 |
| ASGC Remoteness 2006 (2016 for Wave 11) |  |  |  |  |
| Major Cities of Australia | 224 | 71 | 295 | 29.21% |
| Inner Regional Australia | 195 | 56 | 251 | 24.85% |
| Outer Regional Australia | 115 | 37 | 152 | 15.05% |
| Remote Australia | 107 | 22 | 129 | 12.77% |
| Very Remote Australia | 170 | 13 | 183 | 18.12% |
| Missing | 0 | 0 | 0 | 0 |
| Parent Indigenous Status |  |  |  |  |
| Aboriginal | 587 | 135 | 722 | 71.49% |
| Torres Strait Islander | 52 | 3 | 55 | 5.45% |
| Both | 36 | 6 | 42 | 4.16% |
| Neither | 136 | 55 | 191 | 18.91% |
| Missing | 0 | 0 | 0 | 0 |
| Parent Relationship to child |  |  |  |  |
| Biological Mother | 753 | 187 | 940 | 93.07% |
| Biological Father | 13 | 1 | 14 | 1.39% |
| Grandmother | 30 | 6 | 36 | 3.56% |
| Grandfather | 0 | 1 | 1 | 0.10% |
| Other | 15 | 4 | 19 | 1.88% |

S2.2

*Demographic Details for Participating Children Aged 4-5 Years*

|  | Wave 2 | Wave 3 | Wave 4 | Wave 6 | Total | Percentage |
| --- | --- | --- | --- | --- | --- | --- |
| Sample size | 118 | 237 | 11 | 276 | 642 | 100.00% |
| Sex |  |  |  |  |  |  |
| Male | 60 | 120 | 5 | 142 | 327 | 50.93% |
| Female | 58 | 117 | 6 | 134 | 315 | 49.07% |
| Indigenous status |  |  |  |  |  |  |
| Aboriginal | 98 | 216 | 11 | 238 | 563 | 87.69% |
| Torres Strait Islander | 13 | 11 | 0 | 24 | 48 | 7.48% |
| Both | 7 | 10 | 0 | 14 | 31 | 4.83% |
| Level of Relative Isolation (2016 for Wave 11) |  |  |  |  |  |  |
| None | 41 | 73 | 2 | 91 | 207 | 32.24% |
| Low | 59 | 124 | 3 | 131 | 317 | 49.38% |
| Moderate | 10 | 22 | 6 | 30 | 68 | 10.59% |
| High/Extreme | 8 | 18 | 0 | 24 | 50 | 7.79% |
| Missing | 0 | 0 | 0 | 0 | 0 | 0.00% |
| ASGC Remoteness 2006 (2016 for Wave 11) |  |  |  |  |  |  |
| Major Cities of Australia | 41 | 73 | 2 | 92 | 208 | 32.40% |
| Inner Regional Australia | 18 | 67 | 1 | 81 | 167 | 26.01% |
| Outer Regional Australia | 16 | 30 | 1 | 34 | 81 | 12.62% |
| Remote Australia | 27 | 34 | 2 | 19 | 82 | 12.77% |
| Very Remote Australia | 16 | 33 | 5 | 50 | 104 | 16.20% |
| Missing | 0 | 0 | 0 | 0 | 0 | 0.00% |
| Parent Indigenous Status |  |  |  |  |  |  |
| Aboriginal | 80 | 179 | 11 | 186 | 456 | 71.03% |
| Torres Strait Islander | 14 | 14 | 0 | 21 | 49 | 7.63% |
| Both | 3 | 3 | 0 | 8 | 14 | 2.18% |
| Neither | 21 | 41 | 0 | 61 | 123 | 19.16% |
| Missing | 0 | 0 | 0 | 0 | 0 | 0.00% |
| Parent Relationship to child |  |  |  |  |  |  |
| Biological Mother | 109 | 210 | 9 | 246 | 574 | 89.41% |
| Biological Father | 2 | 6 | 0 | 8 | 16 | 2.49% |
| Grandmother | 5 | 15 | 2 | 14 | 36 | 5.61% |
| Grandfather | 0 | 0 | 0 | 0 | 0 | 0.00% |
| Other | 2 | 6 | 0 | 8 | 16 | 2.49% |

S2.3

*Demographic Details for Participating Children Aged 6-7 Years*

|  | Wave 2 | Wave 3 | Wave 4 | Wave 5 | Wave 6 | Total | Percentage |
| --- | --- | --- | --- | --- | --- | --- | --- |
| Sample size | 2 | 347 | 505 | 113 | 468 | 1435 | 100.00% |
| Sex |  |  |  |  |  |  |  |
| Male | 1 | 176 | 257 | 58 | 235 | 727 | 50.66% |
| Female | 1 | 171 | 248 | 55 | 233 | 708 | 49.34% |
| Indigenous status |  |  |  |  |  |  |  |
| Aboriginal | 1 | 304 | 448 | 101 | 405 | 1259 | 87.74% |
| Torres Strait Islander | 1 | 24 | 33 | 6 | 27 | 91 | 6.34% |
| Both | 0 | 19 | 24 | 6 | 36 | 85 | 5.92% |
| Level of Relative Isolation (2016 for Wave 11) |  |  |  |  |  |  |  |
| None | 0 | 75 | 137 | 31 | 119 | 362 | 25.23% |
| Low | 0 | 173 | 247 | 60 | 236 | 716 | 49.90% |
| Moderate | 2 | 47 | 56 | 14 | 75 | 194 | 13.52% |
| High/Extreme | 0 | 52 | 65 | 8 | 38 | 163 | 11.36% |
| Missing | 0 | 0 | 0 | 0 | 0 | 0 | 0.00% |
| ASGC Remoteness 2006 (2016 for Wave 11) |  |  |  |  |  |  |  |
| Major Cities of Australia | 0 | 75 | 137 | 31 | 120 | 363 | 25.30% |
| Inner Regional Australia | 0 | 92 | 139 | 40 | 111 | 382 | 26.62% |
| Outer Regional Australia | 0 | 50 | 73 | 14 | 90 | 227 | 15.82% |
| Remote Australia | 1 | 39 | 50 | 9 | 56 | 155 | 10.80% |
| Very Remote Australia | 1 | 91 | 106 | 19 | 91 | 308 | 21.46% |
| Missing | 0 | 0 | 0 | 0 | 0 | 0 | 0.00% |
| Parent Indigenous Status |  |  |  |  |  |  |  |
| Aboriginal | 1 | 262 | 373 | 84 | 341 | 1061 | 73.94% |
| Torres Strait Islander | 1 | 27 | 38 | 8 | 29 | 103 | 7.18% |
| Both | 0 | 9 | 12 | 3 | 25 | 49 | 3.41% |
| Neither | 0 | 49 | 82 | 18 | 73 | 222 | 15.47% |
| Missing | 0 | 0 | 0 | 0 | 0 | 0 | 0.00% |
| Parent Relationship to child |  |  |  |  |  |  |  |
| Biological Mother | 2 | 9 | 445 | 100 | 415 | 971 | 67.67% |
| Biological Father | 0 | 305 | 12 | 4 | 10 | 331 | 23.07% |
| Grandmother | 0 | 15 | 29 | 5 | 24 | 73 | 5.09% |
| Grandfather | 0 | 0 | 0 | 0 | 1 | 1 | 0.07% |
| Other | 0 | 18 | 19 | 4 | 18 | 59 | 4.11% |

S2.4

*Demographic Details for Participating Children Aged 8-9 Years*

|  | Wave 4 | Wave 5 | Wave 6 | Wave 8 | Wave 9 | Wave 10 | Wave 11 | Total | Percentage |
| --- | --- | --- | --- | --- | --- | --- | --- | --- | --- |
| Sample size | 19 | 100 | 480 | 456 | 347 | 304 | 3 | 1709 | 100% |
| Sex |  |  |  |  |  |  |  |  |  |
| Male | 10 | 50 | 236 | 219 | 167 | 160 | 1 | 843 | 49.33% |
| Female | 9 | 50 | 244 | 237 | 180 | 144 | 2 | 866 | 50.67% |
| Indigenous status |  |  |  |  |  |  |  |  |  |
| Aboriginal | 18 | 90 | 419 | 396 | 323 | 261 | 2 | 1509 | 88.30% |
| Torres Strait Islander | 1 | 7 | 36 | 29 | 7 | 24 | 0 | 104 | 6.09% |
| Both | 0 | 3 | 25 | 31 | 17 | 19 | 1 | 96 | 5.62% |
| Level of Relative Isolation (2016 for Wave 11) |  |  |  |  |  |  |  |  |  |
| None | 3 | 33 | 131 | 121 | 87 | 104 | 1 | 480 | 28.09% |
| Low | 6 | 43 | 255 | 229 | 212 | 148 | 2 | 895 | 52.37% |
| Moderate | 9 | 13 | 42 | 65 | 27 | 37 | 0 | 193 | 11.29% |
| High/Extreme | 1 | 11 | 52 | 41 | 20 | 15 | 0 | 140 | 8.19% |
| Missing | 0 | 0 | 0 | 0 | 1 | 0 | 0 | 1 | 0.06% |
| ASGC Remoteness 2006 (2016 for Wave 11) |  |  |  |  |  |  |  |  |  |
| Major Cities of Australia | 3 | 33 | 131 | 124 | 88 | 105 | 1 | 485 | 28.38% |
| Inner Regional Australia | 5 | 27 | 142 | 109 | 108 | 74 | 0 | 465 | 27.21% |
| Outer Regional Australia | 0 | 13 | 77 | 90 | 73 | 54 | 2 | 309 | 18.08% |
| Remote Australia | 2 | 8 | 47 | 46 | 36 | 28 | 0 | 167 | 9.77% |
| Very Remote Australia | 9 | 19 | 83 | 87 | 41 | 43 | 0 | 282 | 16.50% |
| Missing | 0 | 0 | 0 | 0 | 0 | 0 | 0 | 0 | 0.00% |
| Parent Indigenous Status |  |  |  |  |  |  |  |  |  |
| Aboriginal | 17 | 68 | 341 | 334 | 256 | 200 | 2 | 1218 | 71.27% |
| Torres Strait Islander | 1 | 7 | 41 | 31 | 4 | 22 | 0 | 106 | 6.20% |
| Both | 0 | 1 | 13 | 23 | 12 | 14 | 1 | 64 | 3.74% |
| Neither | 1 | 24 | 84 | 67 | 74 | 67 | 0 | 317 | 18.55% |
| Missing | 0 | 0 | 1 | 1 | 1 | 1 | 0 | 4 | 0.23% |
| Parent Relationship to child |  |  |  |  |  |  |  |  |  |
| Biological Mother | 14 | 89 | 413 | 378 | 294 | 259 | 3 | 1450 | 84.84% |
| Biological Father | 1 | 4 | 13 | 19 | 10 | 12 | 0 | 59 | 3.45% |
| Grandmother | 2 | 4 | 33 | 32 | 21 | 13 | 0 | 105 | 6.14% |
| Grandfather | 0 | 0 | 1 | 3 | 2 | 1 | 0 | 7 | 0.41% |
| Other | 2 | 3 | 20 | 24 | 20 | 19 | 0 | 88 | 5.15% |

S2.5

*Demographic Details for Participating Children Aged 10-12 Years*

|  | Wave 6 | Wave 8 | Wave 9 | Wave 10 | Wave 11 | Total | Percentage |
| --- | --- | --- | --- | --- | --- | --- | --- |
| Sample size | 14 | 490 | 227 | 702 | 381 | 1814 | 100.00% |
| Sex |  |  |  |  |  |  |  |
| Male | 5 | 249 | 112 | 324 | 187 | 877 | 48.35% |
| Female | 9 | 241 | 115 | 378 | 194 | 937 | 51.65% |
| Indigenous status |  |  |  |  |  |  |  |
| Aboriginal | 13 | 439 | 205 | 613 | 346 | 1616 | 89.08% |
| Torres Strait Islander | 1 | 28 | 10 | 40 | 15 | 94 | 5.18% |
| Both | 0 | 23 | 12 | 49 | 20 | 104 | 5.73% |
| Level of Relative Isolation (2016 for Wave 11) |  |  |  |  |  |  |  |
| None | 3 | 128 | 62 | 177 | 113 | 483 | 26.63% |
| Low | 5 | 256 | 135 | 355 | 222 | 973 | 53.64% |
| Moderate | 6 | 57 | 17 | 106 | 30 | 216 | 11.91% |
| High/Extreme | 0 | 49 | 12 | 64 | 13 | 138 | 7.61% |
| Missing | 0 | 0 | 1 | 0 | 3 | 4 | 0.22% |
| ASGC Remoteness 2006 (2016 for Wave 11) |  |  |  |  |  |  |  |
| Major Cities of Australia | 3 | 128 | 62 | 179 | 115 | 487 | 26.85% |
| Inner Regional Australia | 4 | 149 | 79 | 193 | 128 | 553 | 30.49% |
| Outer Regional Australia | 1 | 72 | 42 | 112 | 63 | 290 | 15.99% |
| Remote Australia | 0 | 48 | 22 | 77 | 41 | 188 | 10.36% |
| Very Remote Australia | 6 | 93 | 21 | 141 | 34 | 295 | 16.26% |
| Missing | 0 | 0 | 1 | 0 | 0 | 1 | 0.06% |
| Parent Indigenous Status |  |  |  |  |  |  |  |
| Aboriginal | 10 | 353 | 160 | 506 | 275 | 1304 | 71.89% |
| Torres Strait Islander | 1 | 31 | 10 | 44 | 11 | 97 | 5.35% |
| Both | 0 | 12 | 8 | 32 | 11 | 63 | 3.47% |
| Neither | 3 | 93 | 48 | 118 | 84 | 346 | 19.07% |
| Missing | 0 | 1 | 1 | 2 | 0 | 4 | 0.22% |
| Parent Relationship to child |  |  |  |  |  |  |  |
| Biological Mother | 11 | 415 | 188 | 563 | 314 | 1491 | 82.19% |
| Biological Father | 1 | 16 | 10 | 38 | 14 | 79 | 4.36% |
| Grandmother | 2 | 32 | 16 | 54 | 27 | 131 | 7.22% |
| Grandfather | 0 | 1 | 1 | 4 | 1 | 7 | 0.39% |
| Other | 0 | 26 | 12 | 43 | 25 | 106 | 5.84% |

S2.6

*Demographic Details for Participating Children Aged 13-15 Years*

|  | Wave 9 | Wave 10 | Wave 11 | Total | Percentage |
| --- | --- | --- | --- | --- | --- |
| Sample size | 4 | 264 | 130 | 398 | 100.00% |
| Sex |  |  |  |  |  |
| Male | 2 | 138 | 63 | 203 | 51.01% |
| Female | 2 | 126 | 67 | 195 | 48.99% |
| Indigenous status |  |  |  |  |  |
| Aboriginal | 3 | 233 | 117 | 353 | 88.69% |
| Torres Strait Islander | 1 | 24 | 6 | 31 | 7.79% |
| Both | 0 | 7 | 7 | 14 | 3.52% |
| Level of Relative Isolation (2016 for Wave 11) |  |  |  |  |  |
| None | 1 | 64 | 50 | 115 | 28.89% |
| Low | 2 | 135 | 60 | 197 | 49.50% |
| Moderate | 0 | 34 | 12 | 46 | 11.56% |
| High/Extreme | 1 | 31 | 7 | 39 | 9.80% |
| Missing | 0 | 0 | 1 | 1 | 0.25% |
| ASGC Remoteness 2006 (2016 for Wave 11) |  |  |  |  |  |
| Major Cities of Australia | 1 | 66 | 50 | 117 | 29.40% |
| Inner Regional Australia | 2 | 69 | 45 | 116 | 29.15% |
| Outer Regional Australia | 0 | 37 | 12 | 49 | 12.31% |
| Remote Australia | 0 | 35 | 6 | 41 | 10.30% |
| Very Remote Australia | 1 | 57 | 17 | 75 | 18.84% |
| Missing | 0 | 0 | 0 | 0 | 0.00% |
| Parent Indigenous Status |  |  |  |  |  |
| Aboriginal | 2 | 186 | 86 | 274 | 68.84% |
| Torres Strait Islander | 1 | 23 | 7 | 31 | 7.79% |
| Both | 0 | 4 | 3 | 7 | 1.76% |
| Neither | 1 | 51 | 34 | 86 | 21.61% |
| Missing | 0 | 0 | 0 | 0 | 0.00% |
| Parent Relationship to child |  |  |  |  |  |
| Biological Mother | 3 | 216 | 108 | 327 | 82.16% |
| Biological Father | 0 | 11 | 1 | 12 | 3.02% |
| Grandmother | 1 | 17 | 13 | 31 | 7.79% |
| Grandfather | 0 | 0 | 1 | 1 | 0.25% |
| Other | 0 | 20 | 7 | 27 | 6.78% |
