# Supplementary material for: Against the use of the Strengths and Difficulties Questionnaire for Aboriginal and Torres Strait Islander children aged 2–15 years
Source: Aust N Z J Psychiatry. 2023 Mar 28;57(10):1343–58. doi: 10.1177/00048674231161504 (PMC10517593; doi:10.1177/00048674231161504)
Supplement: sj-docx-3-anp-10.1177_00048674231161504 – Supplemental material for Against the use of the Strengths and Difficulties Questionnaire for Aboriginal and Torres Strait Islander children aged 2–15 years [file sj-docx-3-anp-10.1177_00048674231161504.docx]

S3.1

*Descriptive Statistics for the Parent-Reported Strengths and Difficulties Questionnaire in Indigenous Children Aged 2 – 4 Years*

|  |  | 95% *CI* | |  |  |  |  |  |
| --- | --- | --- | --- | --- | --- | --- | --- | --- |
| Scale | *M* | *LL* | *UL* | *MD* | *SD* | *Range* | *ω* | *n* |
| Emotional Symptoms | 2.19 | 2.07 | 2.30 | 2.00 | 1.681 | 0 – 10 | .39 | 803 |
| Female | 2.23 | 2.07 | 2.38 | 2.00 | 1.571 | 0 – 8 | .36 | 395 |
| Male | 2.15 | 1.98 | 2.32 | 2.00 | 1.782 | 0 – 10 | .46 | 408 |
| Conduct Problems | 3.73 | 3.56 | 3.89 | 4.00 | 2.360 | 0 – 10 | .68 | 791 |
| Female | 3.65 | 3.42 | 3.00 | 3.00 | 2.258 | 0 – 10 | .66 | 392 |
| Male | 3.81 | 3.57 | 4.05 | 4.00 | 2.457 | 0 – 10 | .69 | 399 |
| Hyperactivity | 4.70 | 4.52 | 4.88 | 5.00 | 2.494 | 0 – 10 | .64 | 760 |
| Female | 4.44 | 4.19 | 4.70 | 5.00 | 2.517 | 0 – 10 | .67 | 371 |
| Male | 4.95 | 4.70 | 5.19 | 5.00 | 2.449 | 0 – 10 | .61 | 389 |
| Peer Problems | 2.21 | 2.10 | 2.33 | 2.00 | 1.639 | 0 - 9 | .24 | 776 |
| Female | 2.12 | 1.95 | 2.28 | 2.00 | 1.619 | 0 – 7 | .23 | 384 |
| Male | 2.31 | 2.14 | 2.47 | 2.00 | 1.656 | 0 – 9 | .27 | 392 |
| Prosocial Scale | 7.94 | 7.81 | 8.07 | 8.00 | 1.865 | 1 – 10 | .64 | 787 |
| Female | 8.13 | 7.95 | 8.30 | 9.00 | 1.779 | 1 – 10 | .64 | 392 |
| Male | 7.76 | 7.57 | 7.95 | 8.00 | 1.932 | 1 – 10 | .64 | 395 |
| Internalising Scale | 4.38 | 4.19 | 4.57 | 4.00 | 2.647 | 0 – 16 | .49 | 769 |
| Male | 4.44 | 4.16 | 4.72 | 4.00 | 2.798 | 0 – 16 | .54 | 390 |
| Female | 4.32 | 4.07 | 4.57 | 4.00 | 2.484 | 0 – 12 | .44 | 379 |
| Externalising Scale | 8.45 | 8.16 | 8.75 | 8.00 | 4.113 | 0 – 20 | .74 | 747 |
| Male | 8.75 | 8.33 | 9.18 | 8.00 | 4.207 | 0 – 20 | .75 | 381 |
| Female | 8.14 | 7.73 | 8.55 | 8.00 | 3.995 | 0 – 20 | .74 | 366 |
| Total Difficulties Scale | 12.75 | 12.35 | 13.15 | 12.00 | 5.491 | 0 – 32 | .73 | 724 |
| Male | 13.11 | 12.52 | 13.69 | 13.00 | 5.722 | 1 – 32 | .74 | 369 |
| Female | 12.38 | 11.84 | 12.93 | 12.00 | 5.222 | 0 – 29 | .70 | 355 |

*Note: M =* mean; *CI* = confidence interval; *LL* – lower limit; *UL* = upper limit; *MD* = median; *SD* = standard deviation; *ω* = McDonald’s omega internal consistency reliability; *n* = size of subsample for which data were available / not missing.

S3.2

*Descriptive Statistics for the Parent-Reported Strengths and Difficulties Questionnaire in Indigenous Children Aged 4 – 5 Years*

|  |  | 95% *CI* | |  |  |  |  |  |
| --- | --- | --- | --- | --- | --- | --- | --- | --- |
| Scale | *M* | *LL* | *UL* | *MD* | *SD* | *Range* | *ω* | *n* |
| Emotional Symptoms | 2.31 | 2.14 | 2.48 | 2.00 | 1.933 | 0 – 10 | .53 | 508 |
| Female | 2.35 | 2.13 | 2.58 | 2.00 | 1.811 | 0 – 8 | .43 | 250 |
| Male | 2.27 | 2.02 | 2.52 | 2.00 | 2.047 | 0 – 10 | .62 | 258 |
| Conduct Problems | 2.43 | 2.26 | 2.61 | 2.00 | 1.998 | 0 – 9 | .64 | 503 |
| Female | 2.36 | 2.10 | 2.62 | 2.00 | 2.041 | 0 – 9 | .68 | 247 |
| Male | 2.50 | 2.26 | 2.74 | 2.00 | 1.956 | 0 – 8 | .60 | 256 |
| Hyperactivity | 4.60 | 4.36 | 4.84 | 5.00 | 2.684 | 0 – 10 | .71 | 485 |
| Female | 4.26 | 3.92 | 4.61 | 4.00 | 2.702 | 0 – 10 | .73 | 240 |
| Male | 4.94 | 4.61 | 5.27 | 5.00 | 2.629 | 0 – 10 | .68 | 245 |
| Peer Problems | 1.99 | 1.85 | 2.14 | 2.00 | 1.618 | 0 – 10 | .24 | 487 |
| Female | 1.89 | 1.69 | 2.09 | 2.00 | 1.593 | 0 – 8 | .24 | 242 |
| Male | 2.09 | 1.89 | 2.30 | 2.00 | 1.641 | 0 – 10 | .24 | 245 |
| Prosocial Scale | 8.52 | 8.36 | 8.67 | 9.00 | 1.738 | 1 – 10 | .65 | 505 |
| Female | 8.69 | 8.49 | 8.90 | 9.00 | 1.644 | 2 – 10 | .66 | 250 |
| Male | 8.35 | 8.12 | 8.57 | 9.00 | 1.812 | 1 – 10 | .65 | 255 |
| Internalising Scale | 4.27 | 4.01 | 4.54 | 4.00 | 2.918 | 0 – 15 | .59 | 482 |
| Male | 4.29 | 3.91 | 4.67 | 4.00 | 3.023 | 0 – 15 | .63 | 241 |
| Female | 4.26 | 3.90 | 4.61 | 4.00 | 2.815 | 0 – 14 | .55 | 241 |
| Externalising Scale | 7.05 | 6.69 | 7.41 | 7.00 | 4.002 | 0 – 18 | .77 | 480 |
| Male | 7.43 | 6.94 | 7.93 | 7.00 | 3.938 | 0 – 18 | .75 | 244 |
| Female | 6.66 | 6.14 | 7.17 | 7.00 | 4.038 | 0 – 18 | .78 | 236 |
| Total Difficulties Scale | 11.24 | 10.70 | 11.77 | 11.00 | 5.883 | 0 – 28 | .79 | 459 |
| Male | 11.60 | 10.84 | 12.36 | 11.00 | 5.828 | 0 – 28 | .78 | 230 |
| Female | 10.87 | 10.10 | 11.64 | 11.00 | 5.928 | 0 – 24 | .79 | 229 |

*Note: M =* mean; *CI* = confidence interval; *LL* – lower limit; *UL* = upper limit; *MD* = median; *SD* = standard deviation; *ω* = McDonald’s omega internal consistency reliability; *n* = size of subsample for which data were available / not missing.

S3.3

*Descriptive Statistics for the Parent-Reported Strengths and Difficulties Questionnaire in Indigenous Children Aged 6 – 7 Years*

|  |  | 95% *CI* | |  |  |  |  |  |
| --- | --- | --- | --- | --- | --- | --- | --- | --- |
| Scale | *M* | *LL* | *UL* | *MD* | *SD* | *Range* | *ω* | *n* |
| Emotional Symptoms | 2.58 | 2.46 | 2.69 | 2.00 | 2.152 | 0 – 10 | .60 | 1303 |
| Female | 2.54 | 2.37 | 2.70 | 2.00 | 2.119 | 0 – 10 | .59 | 644 |
| Male | 2.61 | 2.45 | 2.78 | 2.00 | 2.186 | 0 – 10 | .60 | 659 |
| Conduct Problems | 2.45 | 2.34 | 2.55 | 2.00 | 1.969 | 0 – 10 | .60 | 1294 |
| Female | 2.30 | 2.15 | 2.45 | 2.00 | 1.905 | 0 – 9 | .59 | 635 |
| Male | 2.59 | 2.43 | 2.74 | 2.00 | 2.020 | 0 – 10 | .61 | 659 |
| Hyperactivity | 4.61 | 4.47 | 4.76 | 5.00 | 2.626 | 0 – 10 | .67 | 1264 |
| Female | 4.10 | 3.90 | 4.29 | 4.00 | 2.523 | 0 – 10 | .64 | 629 |
| Male | 5.13 | 4.92 | 5.33 | 5.00 | 2.627 | 0 – 10 | .68 | 635 |
| Peer Problems | 2.12 | 2.02 | 2.21 | 2.00 | 1.720 | 0 – 9 | .41 | 1246 |
| Female | 2.04 | 1.91 | 2.17 | 2.00 | 1.675 | 0 – 9 | .33 | 616 |
| Male | 2.19 | 2.06 | 2.033 | 2.00 | 1.761 | 0 – 8 | .45 | 630 |
| Prosocial Scale | 8.58 | 8.49 | 8.67 | 9.00 | 1.680 | 0 – 10 | .62 | 1300 |
| Female | 8.84 | 8.73 | 8.96 | 9.00 | 1.507 | 1 – 10 | .60 | 642 |
| Male | 8.33 | 8.19 | 8.47 | 9.00 | 1.799 | 0 – 10 | .62 | 658 |
| Internalising Scale | 4.70 | 4.52 | 4.88 | 4.00 | 3.184 | 0 – 18 | .64 | 1235 |
| Male | 4.83 | 4.58 | 5.08 | 4.00 | 3.202 | 0 – 17 | .63 | 623 |
| Female | 4.57 | 4.31 | 4.82 | 4.00 | 3.162 | 0 – 18 | .64 | 612 |
| Externalising Scale | 7.05 | 6.83 | 7.27 | 7.00 | 3.915 | 0 – 20 | .74 | 1246 |
| Male | 7.72 | 7.41 | 8.03 | 8.00 | 3.940 | 0 – 20 | .74 | 629 |
| Female | 6.36 | 6.06 | 6.66 | 6.00 | 3.770 | 0 – 18 | .72 | 617 |
| Total Difficulties Scale | 11.64 | 11.30 | 11.99 | 11.00 | 6.006 | 0 – 32 | .78 | 1181 |
| Male | 12.47 | 11.98 | 12.96 | 12.00 | 6.072 | 1 – 31 | .78 | 591 |
| Female | 10.82 | 10.34 | 11.29 | 10.00 | 5.828 | 0 – 32 | .77 | 590 |

*Note: M =* mean; *CI* = confidence interval; *LL* – lower limit; *UL* = upper limit; *MD* = median; *SD* = standard deviation; *ω* = McDonald’s omega internal consistency reliability; *n* = size of subsample for which data were available / not missing.

S3.4

*Descriptive Statistics for the Parent-Reported Strengths and Difficulties Questionnaire in Indigenous Children Aged 8 – 9 Years*

|  |  | 95% *CI* | |  |  |  |  |  |
| --- | --- | --- | --- | --- | --- | --- | --- | --- |
| Scale | *M* | *LL* | *UL* | *MD* | *SD* | *Range* | *ω* | *n* |
| Emotional Symptoms | 2.57 | 2.45 | 2.69 | 2.00 | 2.198 | 0 – 10 | .64 | 1252 |
| Female | 2.54 | 2.37 | 2.71 | 2.00 | 2.217 | 0 – 10 | .67 | 630 |
| Male | 2.59 | 2.42 | 2.76 | 2.00 | 2.180 | 0 – 10 | .63 | 622 |
| Conduct Problems | 2.22 | 2.11 | 2.33 | 2.00 | 1.975 | 0 – 10 | .67 | 1253 |
| Female | 1.98 | 1.83 | 2.12 | 2.00 | 1.861 | 0 – 10 | .65 | 631 |
| Male | 2.46 | 2.30 | 2.62 | 2.00 | 2.057 | 0 – 10 | .68 | 615 |
| Hyperactivity | 4.35 | 4.21 | 4.50 | 4.00 | 2.638 | 0 – 10 | .72 | 1238 |
| Female | 3.91 | 3.70 | 4.11 | 4.00 | 2.575 | 0 – 10 | .71 | 623 |
| Male | 4.81 | 4.60 | 5.01 | 5.00 | 2.627 | 0 – 10 | .71 | 615 |
| Peer Problems | 2.18 | 2.08 | 2.28 | 2.00 | 1.714 | 0 – 8 | .35 | 1233 |
| Female | 2.07 | 1.94 | 2.20 | 2.00 | 1.634 | 0 – 8 | .32 | 625 |
| Male | 2.30 | 2.15 | 2.44 | 2.00 | 1.787 | 0 – 8 | .38 | 608 |
| Prosocial Scale | 8.49 | 8.59 | 8.66 | 9.00 | 1.727 | 0 – 10 | .67 | 1251 |
| Female | 8.75 | 8.63 | 8.87 | 9.00 | 1.573 | 1 – 10 | .64 | 632 |
| Male | 8.23 | 8.08 | 8.37 | 9.00 | 1.835 | 0 – 10 | .68 | 619 |
| Internalising Scale | 4.75 | 4.57 | 4.94 | 4.00 | 3.274 | 0 – 17 | .67 | 1228 |
| Male | 4.91 | 4.64 | 5.17 | 4.00 | 3.295 | 0 – 17 | .66 | 605 |
| Female | 4.60 | 4.35 | 4.86 | 4.00 | 3.250 | 0 – 17 | .68 | 623 |
| Externalising Scale | 6.56 | 6.34 | 6.79 | 6.00 | 4.019 | 0 – 20 | .79 | 1234 |
| Male | 7.25 | 6.93 | 7.57 | 7.00 | 4.037 | 0 – 20 | .78 | 613 |
| Female | 5.88 | 5.58 | 6.19 | 5.00 | 3.884 | 0 – 19 | .78 | 621 |
| Total Difficulties Scale | 11.27 | 10.92 | 11.63 | 11.00 | 6.321 | 0 – 32 | .82 | 1207 |
| Male | 12.14 | 11.63 | 12.65 | 11.00 | 6.356 | 0 – 32 | .81 | 595 |
| Female | 10.43 | 9.94 | 10.92 | 10.00 | 6.177 | 0 – 30 | .82 | 612 |

*Note: M =* mean; *CI* = confidence interval; *LL* – lower limit; *UL* = upper limit; *MD* = median; *SD* = standard deviation; *ω* = McDonald’s omega internal consistency reliability; *n* = size of subsample for which data were available / not missing.

S3.5

*Descriptive Statistics for the Parent-Reported Strengths and Difficulties Questionnaire in Indigenous Children Aged 10 – 12 Years*

|  |  | 95% *CI* | |  |  |  |  |  |
| --- | --- | --- | --- | --- | --- | --- | --- | --- |
| Scale | *M* | *LL* | *UL* | *MD* | *SD* | *Range* | *ω* | *n* |
| Emotional Symptoms | 2.52 | 2.39 | 2.64 | 2.00 | 2.180 | 0 – 10 | .67 | 1206 |
| Female | 2.54 | 2.37 | 2.72 | 2.00 | 2.232 | 0 – 10 | .69 | 628 |
| Male | 2.48 | 2.31 | 2.66 | 2.00 | 2.123 | 0 – 10 | .65 | 578 |
| Conduct Problems | 1.96 | 1.85 | 2.06 | 2.00 | 1.818 | 0 – 10 | .64 | 1205 |
| Female | 1.82 | 1.68 | 1.96 | 2.00 | 1.751 | 0 – 10 | .63 | 628 |
| Male | 2.10 | 1.95 | 2.25 | 2.00 | 1.879 | 0 – 10 | .65 | 577 |
| Hyperactivity | 3.90 | 3.76 | 4.04 | 4.00 | 2.509 | 0 – 10 | .71 | 1206 |
| Female | 3.37 | 3.18 | 3.55 | 3.00 | 2.330 | 0 – 10 | .66 | 628 |
| Male | 4.48 | 4.27 | 4.69 | 4.00 | 2.568 | 0 – 10 | .73 | 578 |
| Peer Problems | 2.23 | 2.12 | 2.33 | 2.00 | 1.784 | 0 – 10 | .38 | 1206 |
| Female | 2.19 | 2.05 | 2.33 | 2.00 | 1.772 | 0 – 8 | .42 | 628 |
| Male | 2.27 | 2.12 | 2.41 | 2.00 | 1.798 | 0 – 10 | .36 | 578 |
| Prosocial Scale | 8.33 | 8.22 | 8.43 | 9.00 | 1.871 | 0 – 10 | .71 | 1206 |
| Female | 8.57 | 8.43 | 8.71 | 9.00 | 1.734 | 0 – 10 | .69 | 628 |
| Male | 8.06 | 7.90 | 8.22 | 8.50 | 1.977 | 0 – 10 | .71 | 578 |
| Internalising Scale | 4.74 | 4.56 | 4.93 | 4.00 | 3.256 | 0 – 16 | .67 | 1206 |
| Male | 4.75 | 4.50 | 5.01 | 4.00 | 3.130 | 0 – 16 | .63 | 578 |
| Female | 4.73 | 4.47 | 5.00 | 4.00 | 3.370 | 0 – 16 | .71 | 628 |
| Externalising Scale | 5.86 | 5.64 | 6.07 | 5.00 | 3.763 | 0 – 20 | .77 | 1205 |
| Male | 6.58 | 6.27 | 6.90 | 6.00 | 3.871 | 0 – 20 | .78 | 577 |
| Female | 5.19 | 4.91 | 5.46 | 5.00 | 3.533 | 0 – 20 | .75 | 628 |
| Total Difficulties Scale | 10.60 | 10.26 | 10.94 | 10.00 | 6.066 | 0 – 34 | .81 | 1205 |
| Male | 11.34 | 10.84 | 11.83 | 11.00 | 6.017 | 0 – 30 | .80 | 577 |
| Female | 9.92 | 9.45 | 10.39 | 9.00 | 6.037 | 0 – 34 | .82 | 628 |

*Note: M =* mean; *CI* = confidence interval; *LL* – lower limit; *UL* = upper limit; *MD* = median; *SD* = standard deviation; *ω* = McDonald’s omega internal consistency reliability; *n* = size of subsample for which data were available / not missing.

S3.6

*Descriptive Statistics for the Parent-Reported Strengths and Difficulties Questionnaire in Indigenous Children Aged 13 – 14 Years*

|  |  | 95% *CI* | |  |  |  |  |  |
| --- | --- | --- | --- | --- | --- | --- | --- | --- |
| Scale | *M* | *LL* | *UL* | *MD* | *SD* | *Range* | *ω* | *n* |
| Emotional Symptoms | 2.57 | 2.30 | 2.83 | 2.00 | 2.186 | 0 – 10 | .72 | 264 |
| Female | 2.60 | 2.23 | 2.98 | 2.00 | 2.220 | 0 – 10 | .72 | 126 |
| Male | 2.53 | 2.15 | 2.91 | 2.00 | 2.157 | 0 – 10 | .72 | 138 |
| Conduct Problems | 1.98 | 1.76 | 2.19 | 2.00 | 1.772 | 0 – 9 | .61 | 264 |
| Female | 2.01 | 1.72 | 2.31 | 2.00 | 1.767 | 0 – 8 | .63 | 126 |
| Male | 1.94 | 1.62 | 2.25 | 2.00 | 1.783 | 0 – 9 | .60 | 138 |
| Hyperactivity | 4.40 | 4.19 | 4.62 | 4.00 | 1.774 | 0 – 10 | .32 | 264 |
| Female | 4.14 | 3.86 | 4.43 | 4.00 | 1.604 | 0 – 9 | .12 | 126 |
| Male | 4.64 | 4.32 | 4.96 | 4.00 | 1.891 | 0 – 10 | .41 | 138 |
| Peer Problems | 2.39 | 2.17 | 2.60 | 2.00 | 1.781 | 0 – 10 | .46 | 264 |
| Female | 2.19 | 1.90 | 2.18 | 2.00 | 1.633 | 0 – 8 | .40 | 126 |
| Male | 2.57 | 2.25 | 2.88 | 2.00 | 1.895 | 0 – 10 | .49 | 138 |
| Prosocial Scale | 7.91 | 7.67 | 8.16 | 8.00 | 2.012 | 0 – 10 | .72 | 264 |
| Female | 8.25 | 7.92 | 8.57 | 9.00 | 1.858 | 3 – 10 | .72 | 126 |
| Male | 7.61 | 7.25 | 7.96 | 8.00 | 2.105 | 0 – 10 | .73 | 138 |
| Internalising Scale | 4.95 | 4.54 | 5.37 | 4.00 | 3.445 | 0 – 18 | .75 | 264 |
| Male | 5.17 | 4.56 | 5.77 | 4.00 | 3.600 | 0 – 18 | .75 | 138 |
| Female | 4.72 | 4.15 | 5.30 | 4.00 | 3.266 | 0 – 17 | .74 | 126 |
| Externalising Scale | 6.38 | 6.03 | 6.73 | 6.00 | 2.862 | 1 – 16 | .61 | 264 |
| Male | 6.65 | 6.16 | 7.16 | 6.00 | 3.013 | 1 – 16 | .64 | 138 |
| Female | 6.08 | 5.61 | 6.55 | 6.00 | 2.667 | 1 – 14 | .57 | 126 |
| Total Difficulties Scale | 11.33 | 10.68 | 11.99 | 10.00 | 5.431 | 1 – 31 | .78 | 264 |
| Male | 11.82 | 10.86 | 12.78 | 11.00 | 5.709 | 2 – 30 | .79 | 138 |
| Female | 10.80 | 9.91 | 11.70 | 10.00 | 5.079 | 1 – 31 | .76 | 126 |

*Note: M =* mean; *CI* = confidence interval; *LL* – lower limit; *UL* = upper limit; *MD* = median; *SD* = standard deviation; *ω* = McDonald’s omega internal consistency reliability; *n* = size of subsample for which data were available / not missing.

S3.7

*Descriptive Statistics for the Teacher-Reported Strengths and Difficulties Questionnaire in Indigenous Children Aged 2 – 4 Years*

|  |  | 95% *CI* | |  |  |  |  |  |
| --- | --- | --- | --- | --- | --- | --- | --- | --- |
| Scale | *M* | *LL* | *UL* | *MD* | *SD* | *Range* | *ω* | *n* |
| Emotional Symptoms | .94 | .74 | 1.14 | 0.00 | 1.666 | 0 – 10 | .81 | 268 |
| Male | 1.13 | .82 | 1.44 | 0.00 | 1.926 | 0 – 10 | .84 | 151 |
| Female | .70 | .48 | .92 | 0.00 | 1.220 | 0 – 8 | .68 | 117 |
| Conduct Problems | 1.49 | 1.24 | 1.75 | 1.00 | 2.170 | 0 – 10 | .83 | 275 |
| Male | 1.72 | 1.35 | 2.09 | 1.00 | 2.343 | 0 – 10 | .85 | 155 |
| Female | 1.20 | .86 | 1.54 | 0.00 | 1.895 | 0 – 9 | .80 | 120 |
| Hyperactivity | 3.41 | 3.07 | 3.74 | 3.00 | 2.796 | 0 – 10 | .88 | 270 |
| Male | 4.19 | 3.74 | 4.64 | 4.00 | 2.825 | 0 – 10 | .87 | 152 |
| Female | 2.40 | 1.96 | 2.84 | 2.00 | 2.418 | 0 – 10 | .85 | 118 |
| Peer Problems | 1.61 | 1.40 | 1.83 | 1.00 | 1.781 | 0 – 9 | .70 | 265 |
| Male | 1.77 | 1.47 | 2.06 | 1.00 | 1.822 | 0 – 7 | .70 | 150 |
| Female | 1.41 | 1.09 | 1.72 | 1.00 | 1.711 | 0 – 9 | .70 | 115 |
| Prosocial Scale | 6.96 | 6.66 | 7.25 | 7.00 | 2.374 | 0 – 10 | .83 | 252 |
| Male | 6.47 | 6.08 | 6.86 | 6.00 | 2.320 | 0 – 10 | .82 | 139 |
| Female | 7.56 | 7.13 | 7.99 | 8.00 | 2.310 | 0 – 10 | .84 | 113 |
| Internalising Scale | 2.40 | 2.06 | 2.74 | 2.00 | 2.799 | 0 – 15 | .78 | 257 |
| Male | 2.71 | 2.21 | 3.20 | 2.00 | 3.008 | 0 – 15 | .79 | 146 |
| Female | 2.00 | 1.54 | 2.46 | 1.00 | 2.453 | 0 – 14 | .75 | 111 |
| Externalising Scale | 4.91 | 4.38 | 5.45 | 3.00 | 4.444 | 0 – 20 | .90 | 265 |
| Male | 5.88 | 5.12 | 6.64 | 4.00 | 4.682 | 0 – 20 | .90 | 150 |
| Female | 3.65 | 2.95 | 4.35 | 2.00 | 3.774 | 0 – 17 | .86 | 115 |
| Total Difficulties Score | 7.08 | 6.36 | 7.79 | 5.00 | 5.735 | 0 – 26 | .86 | 249 |
| Male | 8.13 | 7.15 | 9.11 | 6.00 | 5.882 | 0 – 26 | .85 | 141 |
| Female | 5.70 | 4.70 | 6.71 | 4.50 | 5.253 | 0 – 22 | .85 | 108 |

*Note: M =* mean; *CI* = confidence interval; *LL* – lower limit; *UL* = upper limit; *MD* = median; *SD* = standard deviation; *ω* = McDonald’s omega internal consistency reliability; *n* = size of subsample for which data were available / not missing.

S3.8

*Descriptive Statistics for the Teacher-Reported Strengths and Difficulties Questionnaire in Indigenous Children Aged 4 – 5 Years*

|  |  | 95% *CI* | |  |  |  |  |  |
| --- | --- | --- | --- | --- | --- | --- | --- | --- |
| Scale | *M* | *LL* | *UL* | *MD* | *SD* | *Range* | *ω* | *n* |
| Emotional Symptoms | 1.35 | 1.20 | 1.50 | 1.00 | 1.801 | 0 – 8 | .74 | 579 |
| Male | 1.32 | 1.11 | 1.53 | 1.99 | 1.789 | 0 – 8 | .73 | 284 |
| Female | 1.37 | 1.16 | 1.58 | 1.00 | 1.815 | 0 – 8 | .75 | 295 |
| Conduct Problems | 1.15 | .99 | 1.30 | .00 | 1.851 | 0 – 8 | .81 | 571 |
| Male | 1.47 | 1.22 | 1.71 | .50 | 2.071 | 0 – 8 | .83 | 270 |
| Female | .86 | .68 | 1.04 | .00 | 1.579 | 0 – 8 | .79 | 301 |
| Hyperactivity | 3.50 | 3.25 | 3.75 | 3.00 | 3.101 | 0 – 10 | .90 | 590 |
| Male | 4.56 | 4.19 | 4.93 | 4.00 | 3.208 | 0 – 10 | .90 | 289 |
| Female | 2.48 | 2.18 | 2.78 | 2.00 | 2.622 | 0 – 10 | .85 | 301 |
| Peer Problems | 1.45 | 1.31 | 1.59 | 1.00 | 1.703 | 0 – 9 | .65 | 554 |
| Male | 1.43 | 1.23 | 1.63 | 1.00 | 1.683 | 0 – 8 | .67 | 270 |
| Female | 1.46 | 1.26 | 1.67 | 1.00 | 1.724 | 0 – 9 | .64 | 284 |
| Prosocial Scale | 7.44 | 7.24 | 7.63 | 8.00 | 2.307 | 0 – 10 | .81 | 526 |
| Male | 6.88 | 6.58 | 7.18 | 7.00 | 2.422 | 0 – 10 | .82 | 250 |
| Female | 7.94 | 7.70 | 8.19 | 8.00 | 2.076 | 0 – 10 | .78 | 276 |
| Internalising Scale | 2.70 | 2.46 | 2.94 | 2.00 | 2.841 | 0 – 13 | .74 | 540 |
| Male | 2.70 | 2.36 | 3.05 | 2.00 | 2.863 | 0 – 13 | .74 | 265 |
| Female | 2.71 | 2.37 | 3.04 | 2.00 | 2.825 | 0 – 13 | .73 | 275 |
| Externalising Scale | 4.46 | 4.08 | 4.84 | 3.00 | 4.526 | 0 – 18 | .91 | 551 |
| Male | 5.88 | 5.28 | 6.48 | 5.00 | 4.915 | 0 – 18 | .92 | 262 |
| Female | 3.17 | 2.74 | 3.60 | 2.00 | 3.705 | 0 – 18 | .88 | 289 |
| Total Difficulties Score | 6.91 | 6.39 | 7.43 | 5.00 | 5.932 | 0 – 28 | .87 | 498 |
| Male | 8.30 | 7.49 | 9.11 | 7.00 | 6.357 | 0 – 28 | .88 | 238 |
| Female | 5.64 | 5.00 | 6.27 | 4.00 | 5.210 | 0 – 25 | .84 | 260 |

*Note: M =* mean; *CI* = confidence interval; *LL* – lower limit; *UL* = upper limit; *MD* = median; *SD* = standard deviation; *ω* = McDonald’s omega internal consistency reliability; *n* = size of subsample for which data were available / not missing.

S3.9

*Descriptive Statistics for the Teacher-Reported Strengths and Difficulties Questionnaire in Indigenous Children Aged 6 – 7 Years*

|  |  | 95% *CI* | |  |  |  |  |  |
| --- | --- | --- | --- | --- | --- | --- | --- | --- |
| Scale | *M* | *LL* | *UL* | *MD* | *SD* | *Range* | *ω* | *n* |
| Emotional Symptoms | 1.51 | 1.35 | 1.67 | 1.00 | 1.986 | 0 – 10 | .78 | 589 |
| Male | 1.59 | 1.34 | 1.84 | 1.00 | 2.176 | 0 – 10 | .82 | 297 |
| Female | 1.42 | 1.22 | 1.63 | 1.00 | 1.771 | 0 – 10 | .73 | 292 |
| Conduct Problems | 1.28 | 1.11 | 1.46 | 0.00 | 2.108 | 0 – 10 | .85 | 570 |
| Male | 1.66 | 1.37 | 1.94 | 0.00 | 2.410 | 0 – 10 | .86 | 282 |
| Female | .92 | .72 | 1.12 | 0.00 | 1.689 | 0 – 8 | .81 | 288 |
| Hyperactivity | 3.89 | 3.64 | 4.15 | 3.00 | 3.167 | 0 – 10 | .89 | 611 |
| Male | 4.89 | 4.53 | 5.25 | 5.00 | 3.230 | 0 – 10 | .90 | 308 |
| Female | 2.88 | 2.57 | 3.19 | 2.00 | 2.761 | 0 – 10 | .87 | 303 |
| Peer Problems | 1.42 | 1.27 | 1.56 | 1.00 | 1.754 | 0 – 9 | .68 | 572 |
| Male | 1.53 | 1.31 | 1.74 | 1.00 | 1.841 | 0 – 9 | .71 | 289 |
| Female | 1.30 | 1.11 | 1.50 | 1.00 | 1.657 | 0 – 7 | .64 | 283 |
| Prosocial Scale | 7.57 | 7.38 | 7.77 | 8.00 | 2.378 | 0 – 10 | .83 | 568 |
| Male | 6.81 | 6.51 | 7.10 | 7.00 | 2.517 | 0 – 10 | .83 | 280 |
| Female | 8.32 | 8.09 | 8.55 | 9.00 | 1.971 | 0 – 10 | .80 | 288 |
| Internalising Scale | 2.87 | 2.62 | 3.13 | 2.00 | 3.054 | 0 – 17 | .76 | 553 |
| Male | 3.07 | 2.68 | 3.46 | 2.00 | 3.259 | 0 – 17 | .78 | 276 |
| Female | 2.68 | 2.34 | 3.01 | 2.00 | 2.826 | 0 – 12 | .74 | 277 |
| Externalising Scale | 4.97 | 4.58 | 5.36 | 4.00 | 4.724 | 0 – 20 | .91 | 563 |
| Male | 6.36 | 5.75 | 6.95 | 5.00 | 5.049 | 0 – 20 | .91 | 278 |
| Female | 3.63 | 3.17 | 4.09 | 2.00 | 3.952 | 0 – 17 | .89 | 285 |
| Total Difficulties Score | 7.74 | 7.17 | 8.31 | 6.00 | 6.626 | 0 – 32 | .89 | 523 |
| Male | 9.37 | 8.50 | 10.24 | 8.00 | 7.101 | 0 – 32 | .89 | 257 |
| Female | 6.17 | 5.47 | 6.86 | 4.00 | 5.720 | 0 – 25 | .87 | 266 |

*Note: M =* mean; *CI* = confidence interval; *LL* – lower limit; *UL* = upper limit; *MD* = median; *SD* = standard deviation; *ω* = McDonald’s omega internal consistency reliability; *n* = size of subsample for which data were available / not missing.

S3.10

*Descriptive Statistics for the Teacher-Reported Strengths and Difficulties Questionnaire in Indigenous Children Aged 8 – 9 Years*

|  |  | 95% *CI* | |  |  |  |  |  |
| --- | --- | --- | --- | --- | --- | --- | --- | --- |
| Scale | *M* | *LL* | *UL* | *MD* | *SD* | *Range* | *ω* | *n* |
| Emotional Symptoms | 1.64 | 1.50 | 1.78 | 1.00 | 2.036 | 0 – 9 | .78 | 818 |
| Male | 1.81 | 1.61 | 2.02 | 1.00 | 2.098 | 0 – 9 | .77 | 407 |
| Female | 1.46 | 1.27 | 1.65 | 1.00 | 1.959 | 0 – 9 | .79 | 411 |
| Conduct Problems | 1.51 | 1.35 | 1.66 | 0.00 | 2.269 | 0 – 10 | .86 | 800 |
| Male | 2.06 | 1.81 | 2.30 | 1.00 | 2.490 | 0 – 10 | .86 | 392 |
| Female | 0.98 | 0.79 | 1.16 | 0.00 | 1.891 | 0 – 10 | .86 | 408 |
| Hyperactivity | 3.73 | 3.52 | 3.94 | 3.00 | 3.100 | 0 – 10 | .88 | 834 |
| Male | 4.99 | 4.69 | 5.28 | 5.00 | 3.035 | 0 – 10 | .86 | 414 |
| Female | 2.50 | 2.24 | 2.75 | 2.00 | 2.633 | 0 – 10 | .86 | 420 |
| Peer Problems | 1.61 | 1.48 | 1.74 | 1.00 | 1.851 | 0 – 9 | .68 | 799 |
| Male | 1.73 | 1.54 | 1.91 | 1.00 | 1.874 | 0 – 8 | .70 | 397 |
| Female | 1.50 | 1.32 | 1.68 | 1.00 | 1.823 | 0 – 9 | .68 | 410 |
| Prosocial Scale | 7.48 | 7.31 | 7.65 | 8.00 | 2.469 | 0 – 10 | .86 | 810 |
| Male | 6.68 | 6.43 | 6.94 | 7.00 | 2.629 | 0 – 10 | .86 | 400 |
| Female | 8.26 | 8.06 | 8.46 | 9.00 | 2.020 | 2 – 10 | .81 | 410 |
| Internalising Scale | 3.21 | 2.98 | 3.44 | 2.00 | 3.263 | 0 – 16 | .78 | 793 |
| Male | 3.50 | 3.17 | 3.83 | 3.00 | 3.332 | 0 – 16 | .77 | 395 |
| Female | 2.92 | 2.60 | 3.23 | 2.00 | 3.170 | 0 – 15 | .79 | 398 |
| Externalising Scale | 5.15 | 4.81 | 5.49 | 4.00 | 4.874 | 0 – 20 | .91 | 794 |
| Male | 6.95 | 6.45 | 7.45 | 6.00 | 5.031 | 0 – 20 | .90 | 388 |
| Female | 3.42 | 3.03 | 3.81 | 2.00 | 4.028 | 0 – 20 | .89 | 406 |
| Total Difficulties Score | 8.32 | 7.82 | 8.82 | 6.00 | 7.027 | 0 – 32 | .90 | 761 |
| Male | 10.41 | 9.67 | 11.14 | 9.00 | 7.226 | 0 – 32 | .89 | 375 |
| Female | 6.29 | 5.67 | 6.91 | 4.00 | 6.194 | 0 – 28 | .88 | 386 |

*Note: M =* mean; *CI* = confidence interval; *LL* – lower limit; *UL* = upper limit; *MD* = median; *SD* = standard deviation; *ω* = McDonald’s omega internal consistency reliability; *n* = size of subsample for which data were available / not missing.

S3.11

*Descriptive Statistics for the Teacher-Reported Strengths and Difficulties Questionnaire in Indigenous Children Aged 10 – 12 Years*

|  |  | 95% *CI* | |  |  |  |  |  |
| --- | --- | --- | --- | --- | --- | --- | --- | --- |
| Scale | *M* | *LL* | *UL* | *MD* | *SD* | *Range* | *ω* | *n* |
| Emotional Symptoms | 1.91 | 1.77 | 2.05 | 1.00 | 2.224 | 0 – 10 | .81 | 937 |
| Male | 1.93 | 1.72 | 2.15 | 1.00 | 2.255 | 0 – 10 | .81 | 443 |
| Female | 1.88 | 1.69 | 2.08 | 1.00 | 2.198 | 0 – 10 | .81 | 494 |
| Conduct Problems | 1.59 | 1.44 | 1.74 | 0.00 | 2.317 | 0 – 10 | .87 | 925 |
| Male | 2.29 | 2.05 | 2.54 | 1.00 | 2.634 | 0 – 10 | .87 | 439 |
| Female | 0.96 | 0.80 | 1.12 | 0.00 | 1.766 | 0 – 10 | .84 | 486 |
| Hyperactivity | 3.68 | 3.48 | 3.87 | 3.00 | 3.034 | 0 – 10 | .88 | 935 |
| Male | 4.99 | 4.70 | 5.28 | 5.00 | 3.137 | 0 – 10 | .88 | 441 |
| Female | 2.51 | 2.29 | 2.72 | 2.00 | 2.396 | 0 – 10 | .83 | 494 |
| Peer Problems | 1.65 | 1.53 | 1.77 | 1.00 | 1.842 | 0 – 9 | .69 | 935 |
| Male | 1.86 | 1.67 | 2.04 | 1.00 | 1.998 | 0 – 9 | .73 | 446 |
| Female | 1.47 | 1.32 | 1.61 | 1.00 | 1.668 | 0 – 9 | .62 | 489 |
| Prosocial Scale | 7.34 | 7.18 | 7.50 | 8.00 | 2.464 | 0 – 10 | .85 | 935 |
| Male | 6.62 | 6.37 | 6.86 | 7.00 | 2.598 | 0 – 10 | .85 | 443 |
| Female | 8.00 | 7.81 | 8.19 | 9.00 | 2.138 | 0 – 10 | .82 | 492 |
| Internalising Scale | 3.56 | 3.33 | 3.79 | 2.00 | 3.541 | 0 – 18 | .82 | 929 |
| Male | 3.79 | 3.44 | 4.13 | 3.00 | 3.699 | 0 – 17 | .83 | 441 |
| Female | 3.35 | 3.05 | 3.65 | 2.00 | 3.383 | 0 – 18 | .81 | 488 |
| Externalising Scale | 5.24 | 4.92 | 5.56 | 4.00 | 4.910 | 0 – 20 | .91 | 917 |
| Male | 7.25 | 6.75 | 7.74 | 6.00 | 5.278 | 0 – 20 | .91 | 434 |
| Female | 3.43 | 3.10 | 3.77 | 2.00 | 3.729 | 0 – 20 | .88 | 483 |
| Total Difficulties Score | 8.77 | 8.30 | 9.25 | 7.00 | 7.296 | 0 – 34 | .90 | 907 |
| Male | 11.00 | 10.25 | 11.74 | 10.00 | 7.874 | 0 – 34 | .91 | 428 |
| Female | 6.78 | 6.24 | 7.33 | 5.00 | 6.093 | 0 – 29 | .88 | 479 |

*Note: M =* mean; *CI* = confidence interval; *LL* – lower limit; *UL* = upper limit; *MD* = median; *SD* = standard deviation; *ω* = McDonald’s omega internal consistency reliability; *n* = size of subsample for which data were available / not missing.

S3.12

*Descriptive Statistics for the Teacher-Reported Strengths and Difficulties Questionnaire in Indigenous Children Aged 13 – 15 Years*

|  |  | 95% *CI* | |  |  |  |  |  |
| --- | --- | --- | --- | --- | --- | --- | --- | --- |
| Scale | *M* | *LL* | *UL* | *MD* | *SD* | *Range* | *ω* | *n* |
| Emotional Symptoms | 2.01 | 1.73 | 2.30 | 1.00 | 2.172 | 0 – 9 | .80 | 222 |
| Male | 1.87 | 1.46 | 2.27 | 1.00 | 2.154 | 0 – 9 | .82 | 112 |
| Female | 2.16 | 1.75 | 2.58 | 1.00 | 2.191 | 0 – 8 | .78 | 110 |
| Conduct Problems | 1.72 | 1.39 | 2.06 | 1.00 | 2.488 | 0 – 10 | .89 | 218 |
| Male | 2.22 | 1.70 | 2.74 | 1.00 | 2.747 | 0 – 10 | .89 | 110 |
| Female | 1.22 | 0.82 | 1.62 | 0.00 | 2.088 | 0 – 10 | .87 | 108 |
| Hyperactivity | 4.32 | 3.90 | 4.74 | 4.00 | 3.167 | 0 – 10 | .89 | 225 |
| Male | 5.16 | 4.57 | 5.74 | 5.00 | 3.153 | 0 – 10 | .89 | 115 |
| Female | 3.45 | 2.89 | 4.00 | 3.00 | 2.951 | 0 – 10 | .86 | 110 |
| Peer Problems | 1.65 | 1.43 | 1.87 | 1.00 | 1.684 | 0 – 7 | .67 | 222 |
| Male | 1.71 | 1.38 | 2.05 | 1.00 | 1.808 | 0 – 7 | .75 | 112 |
| Female | 1.58 | 1.29 | 1.88 | 1.00 | 1.553 | 0 – 6 | .62 | 110 |
| Prosocial Scale | 6.25 | 5.90 | 6.60 | 6.00 | 2.620 | 0 – 10 | .86 | 218 |
| Male | 5.64 | 5.12 | 6.16 | 5.00 | 2.712 | 0 – 10 | .88 | 108 |
| Female | 6.85 | 6.40 | 7.31 | 7.00 | 2.388 | 0 – 10 | .82 | 110 |
| Internalising Scale | 3.60 | 3.15 | 4.04 | 2.00 | 3.325 | 0 – 14 | .82 | 217 |
| Male | 3.52 | 2.86 | 4.19 | 2.00 | 3.492 | 0 – 14 | .85 | 109 |
| Female | 3.68 | 3.07 | 4.28 | 3.00 | 3.162 | 0 – 12 | .78 | 108 |
| Externalising Scale | 6.02 | 5.31 | 6.72 | 5.00 | 5.226 | 0 – 20 | .93 | 214 |
| Male | 7.45 | 6.41 | 8.50 | 7.00 | 5.461 | 0 – 20 | .92 | 108 |
| Female | 4.56 | 3.68 | 5.43 | 3.00 | 4.555 | 0 – 20 | .91 | 106 |
| Total Difficulties Score | 9.68 | 8.72 | 10.64 | 8.00 | 7.034 | 0 – 29 | .89 | 208 |
| Male | 11.08 | 9.65 | 12.50 | 9.00 | 7.339 | 0 – 29 | .90 | 104 |
| Female | 8.28 | 7.02 | 9.53 | 7.00 | 6.451 | 0 – 29 | .88 | 104 |

*Note: M =* mean; *CI* = confidence interval; *LL* – lower limit; *UL* = upper limit; *MD* = median; *SD* = standard deviation; *ω* = McDonald’s omega internal consistency reliability; *n* = size of subsample for which data were available / not missing.
