# Supplementary material for: Against the use of the Strengths and Difficulties Questionnaire for Aboriginal and Torres Strait Islander children aged 2–15 years
Source: Aust N Z J Psychiatry. 2023 Mar 28;57(10):1343–58. doi: 10.1177/00048674231161504 (PMC10517593; doi:10.1177/00048674231161504)
Supplement: sj-docx-4-anp-10.1177_00048674231161504 – Supplemental material for Against the use of the Strengths and Difficulties Questionnaire for Aboriginal and Torres Strait Islander children aged 2–15 years [file sj-docx-4-anp-10.1177_00048674231161504.docx]

S4.1

| SDQ Items | Factor 1 | | Factor 2 | | Factor 3 | | Factor 4 | | Factor 5 | |
| --- | --- | --- | --- | --- | --- | --- | --- | --- | --- | --- |
|  | Standardised Factor Loading | Standard Error | Standardised Factor Loading | Standard Error | Standardised Factor Loading | Standard Error | Standardised Factor Loading | Standard Error | Standardised Factor Loading | Standard Error |
| 10. Constantly fidgeting or squirming | **.715** | .062 | **.446** | .185 | .017 | .036 | .017 | .027 | -.151 | .091 |
| 2. Restless, overactive, cannot stay still for long | **.604** | .043 | .285 | .160 | .022 | .034 | .188 | .065 | .007 | .034 |
| 15. Easily distracted, concentration wanders | **.497** | .065 | .187 | .124 | -.012 | .042 | .237 | .062 | .084 | .064 |
| 8. Many worries or often seems worried | .037 | .068 | **.679** | .072 | .043 | .059 | -.024 | .070 | .043 | .092 |
| 13. Often unhappy, depressed or tearful | .109 | .070 | **.570** | .079 | -.071 | .055 | .036 | .077 | .001 | .076 |
| 19. Picked on or bullied by other children | -.006 | .058 | **.495** | .074 | -.010 | .054 | -.111 | .076 | .124 | .108 |
| 6. Rather solitary, prefers to play alone | -.041 | .064 | **.486** | .066 | -.113 | .075 | -.023 | .055 | -.206 | .097 |
| 23. Gets along better with adults than with other children | -.021 | .071 | **.471** | .062 | -.025 | .052 | .081 | .067 | -.146 | .089 |
| 24. Many fears, easily scared | -.071 | .058 | **.317** | .081 | .065 | .070 | .108 | .064 | .219 | .088 |
| 9. Helpful if someone is hurt, upset or feeling ill | -.038 | .044 | -.030 | .067 | **.734** | .046 | .049 | .064 | -.005 | .089 |
| 20. Often offers to help others (parents, teachers, other children) | -.182 | .058 | -.007 | .052 | **.622** | .055 | .169 | .096 | .024 | .063 |
| 17. Kind to younger children | .018 | .044 | -.007 | .045 | **.619** | .113 | -.079 | .063 | -.286 | .123 |
| 1. Considerate of other people's feelings | -.006 | .040 | .027 | .058 | **.548** | .047 | -.287 | .074 | .011 | .064 |
| 4. Shares readily with other children, for example toys, treats, pencils | -.010 | .049 | .041 | .047 | **.476** | .058 | **-.371** | .079 | .004 | .059 |
| 22. Can be spiteful to others | -.207 | .063 | .060 | .058 | -.063 | .065 | **.802** | .060 | -.013 | .037 |
| 5. Often loses temper | .079 | .055 | -.019 | .042 | .100 | .061 | **.577** | .054 | .198 | .060 |
| 18. Often argumentative with adults | .029 | .047 | .073 | .072 | .158 | .082 | **.660** | .062 | -.046 | .053 |
| 12. Often fights with other children or bullies them | .044 | .040 | **.306** | .058 | -.095 | .067 | **.423** | .065 | .224 | .086 |
| 7. Generally well behaved, usually does what adults request # | .261 | .054 | -.141 | .113 | **-.432** | .059 | **.329** | .083 | -.022 | .045 |
| 25. Good attention span, sees work through to the end # | **.329** | .076 | -.037 | .043 | -.162 | .180 | -.026 | .043 | **.491** | .073 |
| 3. Often complains of headaches, stomach-aches or sickness | -.034 | .061 | **.328** | .118 | .175 | .143 | .006 | .041 | **.409** | .108 |
| 21. Can stop and think things out before acting # | **.364** | .066 | -.051 | .042 | -.248 | .165 | -.032 | .040 | **.372** | .096 |
| 11. Has at least one good friend # | -.061 | .082 | .060 | .077 | **-.512** | .143 | -.183 | .083 | **.307** | .130 |
| 14. Generally liked by other children # | -.132 | .087 | .066 | .079 | **-.581** | .128 | .059 | .062 | **.302** | .129 |
| 16. Nervous or clingy in new situations, easily loses confidence | -.035 | .054 | .186 | .064 | .088 | .070 | .281 | .061 | -.078 | .075 |

*Factor Structure of Parent-Reported SDQ for Children Aged 2-4 Years*

*Note*. # = Reversed scored item. Bold typeface = primary loading. All primary loadings were *p* < .02.

S4.2

| SDQ Items | Factor 1 | | Factor 2 | | Factor 3 | | Factor 4 | | Factor 5 | |
| --- | --- | --- | --- | --- | --- | --- | --- | --- | --- | --- |
|  | Standardised Factor Loading | Standard Error | Standardised Factor Loading | Standard Error | Standardised Factor Loading | Standard Error | Standardised Factor Loading | Standard Error | Standardised Factor Loading | Standard Error |
| 9. Helpful if someone is hurt, upset or feeling ill | -.285 | .104 | **.819** | .070 | -.001 | .038 | .118 | .087 | .000 | .045 |
| 17. Kind to younger children | -.028 | .096 | **.675** | .055 | -.003 | .053 | -.006 | .071 | -.088 | .085 |
| 4. Shares readily with other children, for example toys, treats, pencils | .071 | .065 | **.526** | .077 | -.254 | .064 | -.031 | .054 | -.168 | .086 |
| 20. Often offers to help others (parents, teachers, other children) | .147 | .102 | **.504** | .076 | -.172 | .075 | .021 | .063 | -.087 | .078 |
| 1. Considerate of other people's feelings | -.292 | .108 | **.496** | .057 | -.202 | .081 | .024 | .042 | -.139 | .081 |
| 15. Easily distracted, concentration wanders | .104 | .118 | .111 | .088 | **.655** | .063 | -.057 | .061 | .115 | .091 |
| 2. Restless, overactive, cannot stay still for long | **.592** | .088 | -.029 | .026 | **.606** | .098 | .008 | .035 | -.056 | .040 |
| 21. Thinks things out before acting # | -.208 | .117 | -.237 | .141 | **.560** | .065 | .056 | .055 | -.034 | .038 |
| 10. Constantly fidgeting or squirming | **.490** | .109 | .073 | .041 | **.546** | .093 | .019 | .054 | .052 | .055 |
| 25. Good attention span, sees chores or homework through to the end # | -.053 | .082 | -.222 | .129 | **.424** | .064 | .287 | .072 | -.051 | .068 |
| 7. Generally well behaved, usually does what adults request # | .017 | .052 | **-.403** | .091 | **.409** | .068 | -.032 | .055 | .192 | .086 |
| 8. Many worries or often seems worried | **.354** | .109 | -.004 | .046 | .054 | .071 | **.515** | .082 | -.032 | .061 |
| 24. Many fears, easily scared | -.051 | .061 | .124 | .080 | .018 | .062 | **.501** | .074 | .168 | .124 |
| 16. Nervous or clingy in new situations, easily loses confidence | .037 | .052 | .117 | .068 | .180 | .065 | **.474** | .073 | .108 | .121 |
| 13. Often unhappy, depressed or tearful | **.449** | .104 | -.118 | .084 | -.002 | .031 | **.461** | .112 | .191 | .131 |
| 6. Rather solitary, prefers to play alone | .024 | .072 | .004 | .059 | .014 | .059 | **.420** | .061 | .120 | .106 |
| 11. Has at least one good friend # | -.033 | .047 | **-.785** | .086 | -.055 | .053 | **.363** | .103 | -.224 | .129 |
| 18. Often lies or cheats | -.009 | .037 | -.018 | .031 | -.025 | .041 | -.241 | .100 | **.828** | .073 |
| 22. Steals from home, school or elsewhere | -.225 | .104 | -.107 | .096 | .036 | .065 | .015 | .047 | **.676** | .090 |
| 12. Often fights with other children or bullies them | .056 | .110 | -.189 | .087 | .055 | .066 | .026 | .065 | **.564** | .077 |
| 19. Picked on or bullied by other children | .191 | .076 | .095 | .072 | .005 | .058 | .062 | .085 | **.439** | .081 |
| 5. Often loses temper | .047 | .070 | .009 | .059 | .243 | .062 | .106 | .069 | **.403** | .081 |
| 23. Gets along better with adults than with other children | .175 | .109 | .103 | .076 | -.100 | .078 | .082 | .079 | **.326** | .095 |
| 3. Often complains of headaches, stomach-aches or sickness | .297 | .092 | -.007 | .064 | -.152 | .082 | .192 | .083 | .233 | .101 |
| 14. Generally liked by other children # | .101 | .094 | **-.767** | .063 | -.025 | .054 | .286 | .091 | -.015 | .063 |

*Factor Structure of Parent-Reported SDQ for Children Aged 4-5 Years*

*Note*. # = Reversed scored item. Bold typeface = primary loading(s). All primary loadings were *p* < .02.

S4.3

| SDQ Items | Factor 1 | | Factor 2 | | Factor 3 | | Factor 4 | | Factor 5 | |
| --- | --- | --- | --- | --- | --- | --- | --- | --- | --- | --- |
|  | Standardised Factor Loading | Standard Error | Standardised Factor Loading | Standard Error | Standardised Factor Loading | Standard Error | Standardised Factor Loading | Standard Error | Standardised Factor Loading | Standard Error |
| 2. Restless, overactive, cannot stay still for long | **.802** | .043 | -.041 | .037 | -.031 | .029 | .003 | .034 | .002 | .043 |
| 10. Constantly fidgeting or squirming | **.797** | .057 | .021 | .028 | .059 | .028 | .100 | .067 | .012 | .040 |
| 15. Easily distracted, concentration wanders | **.445** | .057 | .057 | .044 | -.080 | .046 | .189 | .054 | .111 | .051 |
| 23. Gets along better with adults than with other children | **.315** | .101 | .098 | .077 | -.005 | .021 | -.001 | .034 | **-.446** | .057 |
| 8. Many worries or often seems worried | -.185 | .100 | **.765** | .067 | -.007 | .014 | -.011 | .029 | .165 | .071 |
| 13. Often unhappy, depressed or tearful | .059 | .055 | **.595** | .045 | -.078 | .042 | .122 | .065 | -.062 | .055 |
| 24. Many fears, easily scared | .011 | .033 | **.588** | .044 | .079 | .035 | .106 | .065 | -.010 | .034 |
| 16. Nervous or clingy in new situations, easily loses confidence | .082 | .053 | **.496** | .044 | .105 | .040 | .052 | .054 | -.067 | .052 |
| 3. Often complains of headaches, stomach-aches or sickness | .090 | .055 | **.412** | .056 | .073 | .039 | .027 | .048 | -.012 | .040 |
| 6. Rather solitary, prefers to play alone | .012 | .042 | **.306** | .050 | -.199 | .048 | -.119 | .061 | -.212 | .064 |
| 17. Kind to younger children | .177 | .065 | .058 | .043 | **.790** | .048 | -.135 | .096 | .052 | .053 |
| 9. Helpful if someone is hurt, upset or feeling ill | .004 | .043 | .054 | .046 | **.720** | .044 | .001 | .045 | .206 | .069 |
| 20. Often offers to help others (parents, teachers, other children) | -.113 | .077 | .005 | .038 | **.577** | .045 | .114 | .071 | -.097 | .054 |
| 4. Shares readily with other children, for example toys, treats, pencils | .028 | .058 | -.119 | .047 | **.567** | .040 | -.093 | .064 | -.009 | .042 |
| 1. Considerate of other people's feelings | -.100 | .054 | .064 | .049 | **.527** | .042 | -.184 | .063 | .069 | .047 |
| 18. Often lies or cheats | .034 | .062 | .016 | .036 | .042 | .035 | **.710** | .071 | .044 | .045 |
| 22. Steals from home, school or elsewhere | -.144 | .094 | -.012 | .048 | .006 | .037 | **.680** | .079 | .001 | .047 |
| 12. Often fights with other children or bullies them | .171 | .079 | .094 | .060 | -.086 | .046 | **.453** | .061 | -.195 | .059 |
| 5. Often loses temper | .075 | .063 | .165 | .049 | -.082 | .041 | **.387** | .051 | -.010 | .054 |
| 7. Generally well behaved, usually does what adults request # | .022 | .037 | .017 | .038 | -.472 | .038 | **.325** | .055 | .097 | .049 |
| 21. Thinks things out before acting # | .091 | .106 | -.009 | .032 | -.440 | .051 | .065 | .049 | **.425** | .051 |
| 25. Good attention span, sees chores or homework through to the end # | .141 | .106 | .214 | .063 | -.423 | .047 | -.043 | .032 | **.337** | .052 |
| 19. Picked on or bullied by other children | .138 | .072 | .201 | .067 | -.043 | .042 | .229 | .069 | -.207 | .066 |
| 11. Has at least one good friend # | -.007 | .050 | .140 | .087 | -.609 | .064 | -.202 | .091 | -.030 | .054 |
| 14. Generally liked by other children # | -.124 | .070 | .190 | .080 | -.637 | .042 | .004 | .047 | .058 | .062 |

*Factor Structure of Parent-Reported SDQ for Children Aged 6-7 Years*

*Note*. # = Reversed scored item. Bold typeface = primary loading. All primary loadings were *p* < .02.

S4.4

| SDQ Items | Factor 1 | | Factor 2 | | Factor 3 | | Factor 4 | | Factor 5 | |
| --- | --- | --- | --- | --- | --- | --- | --- | --- | --- | --- |
|  | Standardised Factor Loading | Standard Error | Standardised Factor Loading | Standard Error | Standardised Factor Loading | Standard Error | Standardised Factor Loading | Standard Error | Standardised Factor Loading | Standard Error |
| 10. Constantly fidgeting or squirming | **.783** | .053 | .031 | .026 | .085 | .063 | .073 | .089 | **.331** | .072 |
| 2. Restless, overactive, cannot stay still for long | **.691** | .061 | -.009 | .025 | -.020 | .040 | .210 | .101 | -.080 | .081 |
| 15. Easily distracted, concentration wanders | **.524** | .049 | -.064 | .051 | .075 | .057 | .163 | .073 | .030 | .042 |
| 25. Good attention span, sees chores or homework through to the end # | **.375** | .067 | -.374 | .067 | .023 | .032 | -.033 | .038 | -.098 | .068 |
| 16. Nervous or clingy in new situations, easily loses confidence | **.335** | .073 | .000 | .027 | **.395** | .075 | -.037 | .038 | -.020 | .030 |
| 9. Helpful if someone is hurt, upset or feeling ill | .008 | .043 | **.728** | .051 | .061 | .047 | -.050 | .066 | .242 | .090 |
| 17. Kind to younger children | -.020 | .059 | **.687** | .049 | -.013 | .041 | -.106 | .089 | .056 | .049 |
| 20. Often offers to help others (parents, teachers, other children) | -.205 | .068 | **.606** | .039 | .212 | .049 | .043 | .045 | -.040 | .047 |
| 4. Shares readily with other children, for example toys, treats, pencils | .031 | .045 | **.604** | .040 | -.084 | .043 | -.198 | .067 | .067 | .056 |
| 1. Considerate of other people's feelings | .047 | .050 | **.554** | .041 | .008 | .042 | -.269 | .059 | **.346** | .068 |
| 8. Many worries or often seems worried | .040 | .050 | -.003 | .026 | **.629** | .044 | .116 | .076 | .160 | .079 |
| 13. Often unhappy, depressed or tearful | -.046 | .041 | -.082 | .043 | **.570** | .049 | **.342** | .082 | .105 | .090 |
| 3. Often complains of headaches, stomach-aches or sickness | .140 | .061 | .217 | .045 | **.448** | .056 | .156 | .080 | .008 | .043 |
| 24. Many fears, easily scared | .298 | .070 | .092 | .041 | **.437** | .063 | -.015 | .031 | **-.371** | .057 |
| 11. Has at least one good friend # | .016 | .046 | -.638 | .057 | **.348** | .062 | -.168 | .099 | .056 | .066 |
| 14. Generally liked by other children # | -.102 | .062 | -.728 | .037 | **.326** | .060 | .006 | .025 | .022 | .050 |
| 12. Often fights with other children or bullies them | .024 | .038 | -.110 | .044 | .084 | .052 | **.701** | .046 | -.016 | .039 |
| 18. Often lies or cheats | .113 | .062 | .032 | .033 | -.011 | .037 | **.686** | .057 | -.147 | .068 |
| 5. Often loses temper | -.001 | .036 | -.003 | .031 | .211 | .064 | **.577** | .069 | .158 | .057 |
| 22. Steals from home, school or elsewhere | .039 | .063 | -.008 | .031 | -.085 | .064 | **.574** | .079 | -.067 | .069 |
| 7. Generally well behaved, usually does what adults request # | .056 | .055 | -.415 | .051 | .003 | .038 | **.385** | .056 | .164 | .070 |
| 19. Picked on or bullied by other children | .070 | .053 | .054 | .039 | .286 | .047 | **.334** | .056 | .135 | .077 |
| 6. Rather solitary, prefers to play alone | .091 | .064 | -.204 | .048 | .288 | .062 | -.092 | .059 | -.181 | .063 |
| 21. Thinks things out before acting # | .272 | .083 | -.351 | .064 | -.149 | .058 | .086 | .059 | -.012 | .035 |
| 23. Gets along better with adults than with other children | .051 | .050 | -.096 | .072 | .267 | .076 | .116 | .083 | -.269 | .100 |

*Factor Structure of Parent-Reported SDQ for Children Aged 8-9 Years*

*Note*. # = Reversed scored item. Bold typeface = primary loading. All primary loadings were *p* < .02.

S4.5

*Factor Structure of the Parent-Reported SDQ for Children Aged 10-12 Years*

| SDQ Items | Factor 1 | | Factor 2 | | Factor 3 | | Factor 4 | | Factor 5 | |
| --- | --- | --- | --- | --- | --- | --- | --- | --- | --- | --- |
|  | Standardised Factor Loading | Standard Error | Standardised Factor Loading | Standard Error | Standardised Factor Loading | Standard Error | Standardised Factor Loading | Standard Error | Standardised Factor Loading | Standard Error |
| 2. Restless, overactive, cannot stay still for long | **.845** | .033 | .004 | .019 | -.021 | .023 | -.014 | .024 | -.028 | .031 |
| 10. Constantly fidgeting or squirming | **.779** | .044 | .040 | .025 | .082 | .050 | .050 | .041 | **-.335** | .064 |
| 15. Easily distracted, concentration wanders | **.453** | .060 | .006 | .027 | .148 | .051 | .230 | .045 | .227 | .058 |
| 25. Good attention span, sees chores or homework through to the end # | **.401** | .052 | -.428 | .059 | .030 | .030 | -.020 | .023 | -.073 | .049 |
| 9. Helpful if someone is hurt, upset or feeling ill | .029 | .046 | **.786** | .029 | -.049 | .046 | .021 | .043 | .058 | .049 |
| 17. Kind to younger children | -.006 | .042 | **.748** | .031 | .031 | .053 | -.158 | .051 | -.073 | .039 |
| 20. Often offers to help others (parents, teachers, other children) | .017 | .026 | **.665** | .047 | -.059 | .045 | .048 | .039 | **.309** | .054 |
| 4. Shares readily with other children, for example toys, treats, pencils | .017 | .049 | **.607** | .027 | -.100 | .046 | -.099 | .058 | .023 | .044 |
| 1. Considerate of other people's feelings | .057 | .041 | **.521** | .035 | .030 | .040 | -.419 | .043 | **-.377** | .057 |
| 24. Many fears, easily scared | -.154 | .062 | .089 | .031 | **.828** | .051 | .042 | .032 | **.379** | .051 |
| 16. Nervous or clingy in new situations, easily loses confidence | -.003 | .026 | .026 | .030 | **.722** | .040 | -.017 | .034 | **.311** | .070 |
| 8. Many worries or often seems worried | .065 | .045 | -.026 | .030 | **.686** | .051 | -.176 | .055 | .055 | .048 |
| 13. Often unhappy, depressed or tearful | .076 | .052 | -.140 | .047 | **.515** | .075 | .124 | .067 | .217 | .073 |
| 3. Often complains of headaches, stomach-aches or sickness | .107 | .051 | .042 | .044 | **.333** | .057 | -.012 | .051 | .181 | .056 |
| 5. Often loses temper | .143 | .045 | -.075 | .026 | **.329** | .058 | **.314** | .049 | .096 | .048 |
| 22. Steals from home, school or elsewhere | -.040 | .062 | .055 | .043 | -.023 | .038 | **.770** | .072 | **.320** | .065 |
| 18. Often lies or cheats | .020 | .043 | .028 | .033 | .069 | .058 | **.760** | .057 | -.119 | .060 |
| 12. Often fights with other children or bullies them | .066 | .061 | -.115 | .047 | .131 | .080 | **.560** | .053 | .009 | .025 |
| 19. Picked on or bullied by other children | .010 | .032 | -.007 | .031 | .225 | .059 | **.363** | .054 | -.050 | .048 |
| 21. Thinks things out before acting # | .210 | .057 | -.420 | .060 | -.038 | .032 | .184 | .045 | .078 | .035 |
| 7. Generally well behaved, usually does what adults request # | .134 | .053 | -.516 | .044 | -.026 | .043 | .279 | .043 | -.114 | .064 |
| 23. Gets along better with adults than with other children | .050 | .055 | .102 | .059 | .134 | .053 | .146 | .059 | -.030 | .043 |
| 14. Generally liked by other children # | .007 | .038 | -.770 | .040 | -.045 | .047 | -.014 | .040 | .124 | .062 |
| 6. Rather solitary, prefers to play alone | -.004 | .046 | -.159 | .049 | .295 | .057 | -.026 | .051 | .234 | .057 |
| 11. Has at least one good friend # | -.025 | .056 | -.718 | .047 | .020 | .045 | -.037 | .055 | -.040 | .039 |

*Note*. # = Reversed scored item. Bold typeface = primary loading. All primary loadings were *p* < .02.

S4.6

*Factor Structure of the Parent-Reported SDQ for Children Aged 13-14 Years*

| SDQ Items | Factor 1 | | Factor 2 | | Factor 3 | | Factor 4 | | Factor 5 | |
| --- | --- | --- | --- | --- | --- | --- | --- | --- | --- | --- |
|  | Standardised Factor Loading | Standard Error | Standardised Factor Loading | Standard Error | Standardised Factor Loading | Standard Error | Standardised Factor Loading | Standard Error | Standardised Factor Loading | Standard Error |
| 2. Restless, overactive, cannot stay still for long | **.788** | .075 | -.053 | .064 | .012 | .061 | .033 | .076 | .161 | .123 |
| 10. Constantly fidgeting or squirming | **.764** | .075 | -.028 | .068 | .065 | .100 | -.070 | .102 | -.034 | .067 |
| 15. Easily distracted, concentration wanders | **.600** | .081 | .171 | .082 | .248 | .134 | .025 | .042 | -.283 | .093 |
| 18. Often lies or cheats | **.475** | .221 | .037 | .060 | -.003 | .046 | **.735** | .112 | .063 | .070 |
| 5. Often loses temper | **.433** | .091 | .055 | .077 | .233 | .094 | .000 | .076 | -.056 | .092 |
| 12. Often fights with other children or bullies them | **.423** | .172 | -.137 | .100 | **.413** | .121 | **.311** | .132 | -.105 | .106 |
| 7. Generally well behaved, usually does what adults request # | **.372** | .089 | -.616 | .074 | -.096 | .092 | -.052 | .094 | -.029 | .063 |
| 22. Steals from home, school or elsewhere | **.309** | .235 | -.050 | .097 | .051 | .090 | **.550** | .135 | -.218 | .149 |
| 9. Helpful if someone is hurt, upset or feeling ill | -.015 | .071 | **.653** | .082 | .192 | .152 | -.104 | .128 | .103 | .094 |
| 4. Shares readily with other children, for example toys, treats, pencils | -.044 | .081 | **.678** | .089 | -.109 | .124 | .176 | .160 | .014 | .092 |
| 1. Considerate of other people's feelings | -.046 | .068 | **.679** | .080 | **-.333** | .129 | -.173 | .149 | .017 | .064 |
| 17. Kind to younger children | .053 | .069 | **.478** | .147 | **.606** | .089 | -.099 | .126 | **.382** | .125 |
| 20. Often offers to help others (parents, teachers, other children) | .074 | .069 | **.391** | .101 | -.053 | .087 | -.092 | .095 | **.391** | .095 |
| 8. Many worries or often seems worried | -.057 | .081 | .022 | .071 | **.807** | .068 | .153 | .114 | .087 | .091 |
| 14. Generally liked by other children # | .027 | .077 | -.717 | .099 | **.733** | .081 | -.207 | .177 | -.001 | .071 |
| 24. Many fears, easily scared | .068 | .110 | .009 | .076 | **.706** | .065 | -.240 | .114 | -.012 | .063 |
| 6. Rather solitary, prefers to play alone | -.157 | .091 | -.224 | .086 | **.503** | .091 | .037 | .087 | -.077 | .105 |
| 19. Picked on or bullied by other children | .141 | .117 | -.058 | .088 | **.479** | .086 | .164 | .102 | .086 | .110 |
| 3. Often complains of headaches, stomach-aches or sickness | .082 | .091 | .001 | .089 | **.457** | .082 | -.003 | .092 | -.040 | .090 |
| 21. Thinks things out before acting # | -.073 | .094 | .087 | .156 | .084 | .098 | .004 | .075 | **.769** | .114 |
| 25. Good attention span, sees chores or homework through to the end # | -.079 | .106 | .120 | .124 | .026 | .056 | .026 | .066 | **.645** | .089 |
| 23. Gets along better with adults than with other children | .257 | .125 | .014 | .069 | .243 | .110 | .019 | .099 | **.357** | .089 |
| 13. Often unhappy, depressed or tearful | .079 | .096 | -.030 | .085 | .204 | .096 | .118 | .114 | -.020 | .077 |
| 16. Nervous or clingy in new situations, easily loses confidence | .052 | .051 | .287 | .098 | .030 | .048 | -.061 | .059 | -.408 | .092 |
| 11. Has at least one good friend # | .016 | .067 | -.511 | .110 | .014 | .062 | -.220 | .148 | .071 | .098 |

*Note*. # = Reversed scored item. Bold typeface = primary loading. All primary loadings were *p* < .02.

S4.7

*Factor Structure of the Teacher-Reported SDQ for Children Aged 4-5 Years*

| SDQ Items | Factor 1 | | Factor 2 | | Factor 3 | | Factor 4 | | Factor 5 | |
| --- | --- | --- | --- | --- | --- | --- | --- | --- | --- | --- |
|  | Standardised Factor Loading | Standard Error | Standardised Factor Loading | Standard Error | Standardised Factor Loading | Standard Error | Standardised Factor Loading | Standard Error | Standardised Factor Loading | Standard Error |
| 18. Often lies or cheats | **.870** | .179 | .047 | .063 | .057 | .056 | **-.459** | .077 | .138 | .078 |
| 22. Steals from home, school or elsewhere | **.866** | .140 | -.048 | .061 | .110 | .102 | **-.480** | .103 | -.035 | .037 |
| 12. Often fights with other children or bullies them | **.712** | .121 | .202 | .086 | -.037 | .049 | -.062 | .058 | **.311** | .065 |
| 5. Often loses temper | **.554** | .116 | .242 | .088 | .081 | .060 | -.008 | .052 | .295 | .068 |
| 7. Generally well behaved, usually does what adults request # | **.525** | .080 | **.457** | .062 | .000 | .036 | .092 | .064 | .041 | .059 |
| 14. Generally liked by other children # | **.509** | .113 | .090 | .084 | **.305** | .082 | .250 | .088 | .042 | .050 |
| 21. Thinks things out before acting # | **.446** | .117 | **.423** | .067 | -.059 | .047 | .054 | .054 | -.171 | .061 |
| 10. Constantly fidgeting or squirming | -.037 | .055 | **.953** | .039 | .002 | .039 | -.025 | .040 | .029 | .036 |
| 2. Restless, overactive, cannot stay still for long | .101 | .090 | **.921** | .063 | -.064 | .034 | .022 | .041 | .051 | .049 |
| 15. Easily distracted, concentration wanders | .019 | .059 | **.860** | .030 | .106 | .065 | -.016 | .035 | -.114 | .052 |
| 25. Good attention span, sees chores or homework through to the end # | .169 | .119 | **.647** | .051 | .192 | .080 | -.026 | .030 | **-.303** | .060 |
| 24. Many fears, easily scared | .034 | .060 | -.087 | .068 | **.841** | .040 | -.032 | .076 | -.082 | .097 |
| 13. Often unhappy, depressed or tearful | .173 | .083 | .016 | .066 | **.742** | .038 | -.017 | .073 | .010 | .049 |
| 16. Nervous or clingy in new situations, easily loses confidence | .013 | .058 | .062 | .075 | **.734** | .045 | -.006 | .057 | -.207 | .087 |
| 8. Many worries or often seems worried | -.082 | .106 | .130 | .078 | **.731** | .045 | .005 | .064 | .085 | .079 |
| 11. Has at least one good friend # | .149 | .233 | .166 | .115 | **.301** | .122 | **.571** | .112 | -.014 | .034 |
| 3. Often complains of headaches, stomach-aches or sickness | -.086 | .174 | .101 | .108 | **.559** | .064 | -.069 | .127 | .211 | .106 |
| 6. Rather solitary, prefers to play alone | -.092 | .141 | -.012 | .055 | **.549** | .098 | **.507** | .088 | -.068 | .049 |
| 23. Gets along better with adults than with other children | .052 | .042 | -.070 | .073 | **.428** | .160 | **.466** | .135 | **.501** | .083 |
| 20. Often offers to help others (parents, teachers, other children) | **-.476** | .221 | -.006 | .038 | -.161 | .076 | -.034 | .050 | **.619** | .064 |
| 4. Shares readily with other children, for example toys, treats, pencils | **-.764** | .069 | -.060 | .069 | .061 | .049 | -.069 | .066 | .011 | .064 |
| 1. Considerate of other people's feelings | **-.774** | .105 | -.164 | .087 | .183 | .049 | -.077 | .074 | .040 | .070 |
| 17. Kind to younger children | **-.821** | .075 | .002 | .053 | .088 | .049 | .049 | .071 | .187 | .087 |
| 9. Helpful if someone is hurt, upset or feeling ill | **-.830** | .145 | .203 | .083 | .012 | .029 | -.023 | .045 | **.421** | .084 |
| 19. Picked on or bullied by other children | .279 | .138 | -.068 | .115 | .298 | .112 | .102 | .112 | .195 | .091 |

*Note*. # = Reversed scored item. Bold typeface = primary loading. All primary loadings were *p* < .02.

S4.8

*Factor Structure of the Teacher-Reported SDQ for Children Aged 6-7 Years*

| SDQ Items | Factor 1 | | Factor 2 | | Factor 3 | | Factor 4 | | Factor 5 | |
| --- | --- | --- | --- | --- | --- | --- | --- | --- | --- | --- |
|  | Standardised Factor Loading | Standard Error | Standardised Factor Loading | Standard Error | Standardised Factor Loading | Standard Error | Standardised Factor Loading | Standard Error | Standardised Factor Loading | Standard Error |
| 12. Often fights with other children or bullies them | **.944** | .054 | -.070 | .046 | .105 | .034 | **.314** | .053 | **.665** | .099 |
| 18. Often lies or cheats | **.851** | .052 | **.891** | .047 | .019 | .021 | .101 | .040 | **.601** | .083 |
| 5. Often loses temper | **.788** | .063 | -.002 | .031 | -.054 | .054 | -.005 | .025 | -.045 | .042 |
| 22. Steals from home, school or elsewhere | **.781** | .074 | .009 | .042 | -.034 | .032 | .055 | .041 | -.011 | .034 |
| 14. Generally liked by other children # | **.710** | .057 | -.041 | .054 | **.680** | .040 | .036 | .033 | -.214 | .067 |
| 7. Generally well behaved, usually does what adults request # | **.647** | .053 | .045 | .049 | .040 | .042 | -.030 | .048 | .065 | .054 |
| 11. Has at least one good friend # | **.468** | .111 | .155 | .079 | **.778** | .041 | -.173 | .076 | .035 | .032 |
| 23. Gets along better with adults than with other children | **.388** | .097 | .117 | .059 | .006 | .029 | **.531** | .060 | -.104 | .082 |
| 13. Often unhappy, depressed or tearful | **.363** | .078 | .006 | .043 | .086 | .055 | -.061 | .039 | .003 | .035 |
| 21. Thinks things out before acting # | **.363** | .049 | .088 | .067 | .298 | .063 | .199 | .070 | -.179 | .067 |
| 19. Picked on or bullied by other children | **.538** | .083 | **.902** | .046 | .038 | .032 | -.066 | .042 | .238 | .057 |
| 10. Constantly fidgeting or squirming | .112 | .060 | **.905** | .046 | -.059 | .027 | .033 | .030 | .040 | .058 |
| 6. Rather solitary, prefers to play alone | -.027 | .018 | **.676** | .043 | .114 | .038 | -.218 | .048 | -.045 | .041 |
| 8. Many worries or often seems worried | .067 | .070 | **.431** | .049 | -.097 | .043 | -.221 | .041 | -.032 | .041 |
| 24. Many fears, easily scared | -.018 | .038 | -.027 | .044 | **.965** | .028 | -.113 | .082 | .287 | .104 |
| 4. Shares readily with other children, for example toys, treats, pencils | **-.684** | .044 | -.058 | .053 | **.811** | .039 | .050 | .044 | .136 | .088 |
| 20. Often offers to help others (parents, teachers, other children) | -.053 | .049 | .194 | .093 | **.446** | .061 | .192 | .088 | .072 | .044 |
| 2. Restless, overactive, cannot stay still for long | .083 | .062 | -.150 | .092 | -.124 | .064 | **.776** | .061 | -.083 | .044 |
| 1. Considerate of other people's feelings | **-.746** | .059 | .001 | .028 | -.031 | .035 | **.649** | .044 | -.011 | .032 |
| 3. Often complains of headaches, stomach-aches or sickness | .055 | .095 | .028 | .078 | .166 | .083 | -.025 | .053 | .031 | .039 |
| 25. Good attention span, sees chores or homework through to the end # | .033 | .034 | -.093 | .093 | .162 | .086 | .230 | .080 | .000 | .052 |
| 15. Easily distracted, concentration wanders | -.060 | .050 | -.024 | .040 | .026 | .033 | .269 | .046 | .113 | .102 |
| 16. Nervous or clingy in new situations, easily loses confidence | -.087 | .067 | .126 | .077 | .100 | .051 | .053 | .057 | -.057 | .040 |
| 9. Helpful if someone is hurt, upset or feeling ill | **-.419** | .058 | .081 | .074 | .257 | .102 | -.073 | .074 | -.062 | .064 |
| 17. Kind to younger children | **-.633** | .064 | .280 | .053 | -.050 | .028 | -.136 | .049 | -.043 | .040 |

*Note*. # = Reversed scored item. Bold typeface = primary loading. All primary loadings were *p* < .02.

S4.9

| SDQ Items | Factor 1 | | Factor 2 | | Factor 3 | | Factor 4 | | Factor 5 | |
| --- | --- | --- | --- | --- | --- | --- | --- | --- | --- | --- |
|  | Standardised Factor Loading | Standard Error | Standardised Factor Loading | Standard Error | Standardised Factor Loading | Standard Error | Standardised Factor Loading | Standard Error | Standardised Factor Loading | Standard Error |
| 12. Often fights with other children or bullies them | **.863** | .041 | .002 | .032 | -.113 | .068 | -.010 | .033 | -.035 | .039 |
| 22. Steals from home, school or elsewhere | **.849** | .046 | -.126 | .069 | -.026 | .036 | .145 | .081 | -.023 | .054 |
| 18. Often lies or cheats | **.826** | .040 | .022 | .035 | -.106 | .056 | .055 | .040 | -.033 | .051 |
| 5. Often loses temper | **.739** | .050 | .156 | .060 | -.081 | .081 | .049 | .031 | .004 | .055 |
| 19. Picked on or bullied by other children | **.482** | .066 | -.042 | .049 | .091 | .059 | .165 | .062 | **.342** | .061 |
| 7. Generally well behaved, usually does what adults request # | **.459** | .056 | **.335** | .040 | **-.351** | .054 | -.031 | .024 | .005 | .030 |
| 13. Often unhappy, depressed or tearful | **.453** | .080 | .020 | .031 | .061 | .044 | **.512** | .046 | .219 | .077 |
| 21. Thinks things out before acting # | **.358** | .047 | .277 | .035 | **-.362** | .058 | .156 | .034 | -.118 | .044 |
| 14. Generally liked by other children # | **.336** | .054 | .059 | .037 | **-.509** | .059 | -.050 | .035 | **.379** | .052 |
| 10. Constantly fidgeting or squirming | .195 | .082 | **.855** | .050 | .031 | .023 | -.009 | .019 | .082 | .041 |
| 2. Restless, overactive, cannot stay still for long | .283 | .079 | **.816** | .047 | .027 | .020 | -.059 | .021 | .076 | .039 |
| 15. Easily distracted, concentration wanders | -.049 | .029 | **.769** | .036 | -.178 | .059 | .261 | .042 | -.047 | .027 |
| 25. Good attention span, sees chores or homework through to the end # | .001 | .019 | **.567** | .036 | **-.378** | .050 | **.326** | .035 | -.050 | .023 |
| 9. Helpful if someone is hurt, upset or feeling ill | -.132 | .059 | .010 | .033 | **.818** | .043 | .009 | .031 | -.028 | .038 |
| 17. Kind to younger children | -.284 | .073 | .068 | .035 | **.788** | .054 | .108 | .051 | -.005 | .043 |
| 20. Often offers to help others (parents, teachers, other children) | -.008 | .029 | -.012 | .034 | **.723** | .056 | -.283 | .052 | .173 | .043 |
| 4. Shares readily with other children, for example toys, treats, pencils | -.297 | .055 | -.003 | .031 | **.613** | .052 | -.027 | .032 | -.078 | .044 |
| 1. Considerate of other people's feelings | **-.533** | .047 | -.089 | .038 | **.520** | .050 | .076 | .028 | -.014 | .030 |
| 24. Many fears, easily scared | -.044 | .051 | -.046 | .052 | -.045 | .038 | **.907** | .032 | .032 | .037 |
| 16. Nervous or clingy in new situations, easily loses confidence | .057 | .047 | .054 | .048 | -.039 | .045 | **.742** | .031 | .043 | .049 |
| 8. Many worries or often seems worried | .140 | .074 | -.007 | .038 | .076 | .042 | **.733** | .050 | .153 | .075 |
| 3. Often complains of headaches, stomach-aches or sickness | .141 | .078 | .126 | .059 | .012 | .049 | **.357** | .061 | .218 | .086 |
| 11. Has at least one good friend # | -.019 | .018 | .016 | .032 | **-.635** | .080 | .035 | .023 | **.648** | .038 |
| 23. Gets along better with adults than with other children | .069 | .062 | -.059 | .054 | -.030 | .049 | .042 | .060 | **.570** | .055 |
| 6. Rather solitary, prefers to play alone | -.222 | .086 | -.019 | .034 | **-.430** | .066 | .294 | .059 | **.483** | .056 |

*Factor Structure of the Teacher-Reported SDQ for Children Aged 8-9 Years*

*Note*. # = Reversed scored item. Bold typeface = primary loading. All primary loadings were *p* < .02.

S4.10

*Factor Structure of the Teacher-Reported SDQ for Children Aged 10-12 Years*

| SDQ Items | Factor 1 | | Factor 2 | | Factor 3 | | Factor 4 | | Factor 5 | |
| --- | --- | --- | --- | --- | --- | --- | --- | --- | --- | --- |
|  | Standardised Factor Loading | Standard Error | Standardised Factor Loading | Standard Error | Standardised Factor Loading | Standard Error | Standardised Factor Loading | Standard Error | Standardised Factor Loading | Standard Error |
| 10. Constantly fidgeting or squirming | **.917** | .035 | .034 | .030 | .017 | .034 | -.039 | .020 | .295 | .076 |
| 2. Restless, overactive, cannot stay still for long | **.843** | .044 | .004 | .016 | .149 | .057 | -.085 | .029 | .172 | .080 |
| 15. Easily distracted, concentration wanders | **.744** | .038 | -.238 | .058 | -.006 | .034 | .204 | .046 | -.049 | .031 |
| 25. Good attention span, sees chores or homework through to the end # | **.553** | .038 | **-.444** | .052 | -.005 | .026 | .247 | .044 | -.077 | .038 |
| 9. Helpful if someone is hurt, upset or feeling ill | .034 | .030 | **.903** | .034 | -.018 | .033 | .108 | .045 | -.102 | .082 |
| 20. Often offers to help others (parents, teachers, other children) | -.049 | .039 | **.879** | .039 | .147 | .064 | -.043 | .040 | .052 | .073 |
| 17. Kind to younger children | -.054 | .030 | **.751** | .052 | -.081 | .055 | .170 | .053 | -.208 | .083 |
| 4. Shares readily with other children, for example toys, treats, pencils | .058 | .050 | **.601** | .051 | -.221 | .064 | .034 | .035 | -.189 | .073 |
| 1. Considerate of other people's feelings | -.053 | .040 | **.551** | .057 | **-.490** | .048 | .096 | .031 | .023 | .040 |
| 12. Often fights with other children or bullies them | .132 | .059 | -.079 | .075 | **.802** | .049 | -.037 | .036 | -.003 | .034 |
| 18. Often lies or cheats | .107 | .056 | -.093 | .066 | **.784** | .048 | .022 | .034 | -.040 | .041 |
| 22. Steals from home, school or elsewhere | -.014 | .059 | -.109 | .072 | **.719** | .082 | .017 | .051 | .018 | .070 |
| 5. Often loses temper | **.321** | .056 | -.097 | .055 | **.539** | .054 | .075 | .034 | .124 | .059 |
| 19. Picked on or bullied by other children | .020 | .039 | .069 | .052 | **.460** | .066 | .230 | .051 | .259 | .075 |
| 7. Generally well behaved, usually does what adults request # | .292 | .037 | -.360 | .051 | **.441** | .049 | -.005 | .025 | -.001 | .033 |
| 8. Many worries or often seems worried | -.089 | .041 | .048 | .033 | **.372** | .062 | **.834** | .033 | -.034 | .022 |
| 24. Many fears, easily scared | .056 | .050 | -.111 | .085 | .039 | .039 | **.763** | .040 | .109 | .065 |
| 16. Nervous or clingy in new situations, easily loses confidence | .106 | .058 | -.206 | .073 | -.091 | .047 | **.709** | .036 | .074 | .063 |
| 13. Often unhappy, depressed or tearful | -.049 | .034 | -.002 | .025 | **.472** | .061 | **.639** | .040 | .058 | .048 |
| 6. Rather solitary, prefers to play alone | -.001 | .028 | -.287 | .128 | -.216 | .062 | .133 | .070 | **.661** | .079 |
| 23. Gets along better with adults than with other children | .041 | .053 | .034 | .069 | -.007 | .035 | .000 | .034 | **.651** | .045 |
| 11. Has at least one good friend # | -.102 | .044 | **-.449** | .094 | .068 | .053 | .093 | .052 | **.489** | .072 |
| 14. Generally liked by other children # | .007 | .040 | **-.459** | .070 | **.305** | .062 | .037 | .045 | **.316** | .066 |
| 21. Thinks things out before acting # | .290 | .043 | **-.433** | .052 | .272 | .047 | .044 | .030 | -.084 | .039 |
| 3. Often complains of headaches, stomach-aches or sickness | .149 | .058 | .096 | .067 | .258 | .073 | **.390** | .054 | .140 | .077 |

*Note*. # = Reversed scored item. Bold typeface = primary loading. All primary loadings were *p* < .02.

S4.11

*Factor Structure of the Teacher-Reported SDQ for Children Aged 13-15 Years*

| SDQ Items | Factor 1 | | Factor 2 | | Factor 3 | | Factor 4 | | Factor 5 | |
| --- | --- | --- | --- | --- | --- | --- | --- | --- | --- | --- |
|  | Standardised Factor Loading | Standard Error | Standardised Factor Loading | Standard Error | Standardised Factor Loading | Standard Error | Standardised Factor Loading | Standard Error | Standardised Factor Loading | Standard Error |
| 10. Constantly fidgeting or squirming | **1.091** | .105 | .011 | .045 | -.054 | .053 | -.211 | .226 | -.008 | .052 |
| 2. Restless, overactive, cannot stay still for long | **1.033** | .116 | .021 | .047 | -.047 | .057 | -.105 | .225 | .040 | .051 |
| 15. Easily distracted, concentration wanders | **.814** | .104 | -.013 | .049 | .167 | .099 | .127 | .155 | **-.300** | .081 |
| 25. Good attention span, sees chores or homework through to the end # | **.600** | .100 | -.271 | .086 | .164 | .081 | .068 | .121 | -.286 | .079 |
| 21. Thinks things out before acting # | **.496** | .096 | **-.453** | .079 | -.021 | .048 | .078 | .119 | -.056 | .070 |
| 5. Often loses temper | **.488** | .196 | -.200 | .115 | .026 | .049 | .263 | .213 | **.373** | .088 |
| 7. Generally well behaved, usually does what adults request # | **.422** | .135 | **-.338** | .073 | -.003 | .056 | **.373** | .134 | .055 | .061 |
| 17. Kind to younger children | .011 | .077 | **.951** | .085 | .283 | .082 | -.011 | .072 | .000 | .055 |
| 9. Helpful if someone is hurt, upset or feeling ill | .078 | .106 | **.929** | .059 | .051 | .057 | -.055 | .077 | .018 | .060 |
| 1. Considerate of other people's feelings | -.178 | .110 | **.890** | .078 | **.334** | .064 | -.021 | .074 | .004 | .036 |
| 4. Shares readily with other children, for example toys, treats, pencils | .269 | .106 | **.827** | .097 | -.106 | .084 | -.009 | .068 | .083 | .089 |
| 20. Often offers to help others (parents, teachers, other children) | -.091 | .129 | **.784** | .111 | .057 | .069 | -.028 | .093 | **.411** | .093 |
| 24. Many fears, easily scared | -.112 | .139 | -.141 | .123 | **.887** | .075 | -.007 | .081 | -.192 | .180 |
| 8. Many worries or often seems worried | .057 | .106 | .023 | .080 | **.880** | .073 | -.032 | .097 | .132 | .159 |
| 13. Often unhappy, depressed or tearful | .199 | .136 | .034 | .060 | **.777** | .081 | .030 | .089 | .207 | .170 |
| 16. Nervous or clingy in new situations, easily loses confidence | .085 | .115 | .061 | .095 | **.709** | .081 | .001 | .087 | -.250 | .130 |
| 3. Often complains of headaches, stomach-aches or sickness | .273 | .110 | -.042 | .108 | **.591** | .073 | -.032 | .134 | -.081 | .146 |
| 19. Picked on or bullied by other children | -.077 | .121 | -.223 | .098 | **.543** | .122 | .044 | .101 | .293 | .139 |
| 6. Rather solitary, prefers to play alone | -.021 | .070 | **-.425** | .144 | **.528** | .102 | **-.586** | .137 | .031 | .044 |
| 18. Often lies or cheats | .282 | .158 | -.009 | .047 | .227 | .083 | **.681** | .146 | .060 | .071 |
| 22. Steals from home, school or elsewhere | .046 | .098 | -.257 | .128 | **.360** | .115 | **.620** | .107 | .015 | .061 |
| 12. Often fights with other children or bullies them | **.340** | .244 | -.077 | .104 | .012 | .038 | **.488** | .213 | **.532** | .095 |
| 23. Gets along better with adults than with other children | .038 | .098 | .043 | .095 | .195 | .110 | -.276 | .145 | **.390** | .127 |
| 14. Generally liked by other children # | .081 | .094 | **-.907** | .071 | .090 | .068 | -.212 | .101 | .039 | .084 |
| 11. Has at least one good friend # | .015 | .068 | **-.987** | .148 | .042 | .044 | **-.789** | .166 | .065 | .087 |

*Note*. # = Reversed scored item. Bold typeface = primary loading. All primary loadings were *p* < .02.
