# Supplementary material for: Against the use of the Strengths and Difficulties Questionnaire for Aboriginal and Torres Strait Islander children aged 2–15 years
Source: Aust N Z J Psychiatry. 2023 Mar 28;57(10):1343–58. doi: 10.1177/00048674231161504 (PMC10517593; doi:10.1177/00048674231161504)
Supplement: sj-docx-5-anp-10.1177_00048674231161504 – Supplemental material for Against the use of the Strengths and Difficulties Questionnaire for Aboriginal and Torres Strait Islander children aged 2–15 years [file sj-docx-5-anp-10.1177_00048674231161504.docx]

S5.1

*4 Factor Structure of the Parent-Reported SDQ Difficulties Items for Children Aged 2-4 Years*

| SDQ Items | Factor 1 | | Factor 2 | | Factor 3 | | Factor 4 | |
| --- | --- | --- | --- | --- | --- | --- | --- | --- |
|  | Standardised Factor Loading | Standard Error | Standardised Factor Loading | Standard Error | Standardised Factor Loading | Standard Error | Standardised Factor Loading | Standard Error |
| 2. Restless, overactive, cannot stay still for long | **.692** | .047 | .114 | .096 | .105 | .080 | - .048 | .050 |
| 3. Often complains of headaches, stomach-aches or sickness | - .009 | .058 | .398 | .074 | .002 | .065 | .200 | .083 |
| 5. Often loses temper | .135 | .109 | - .026 | .056 | **.514** | .072 | .041 | .045 |
| 6. Rather solitary, prefers to play alone | - .005 | .064 | **.417** | .060 | .035 | .064 | - .082 | .051 |
| 8. Many worries or often seems worried | .095 | .091 | **.677** | .070 | - .021 | .057 | - .023 | .039 |
| 10. Constantly fidgeting or squirming | **.748** | .049 | .203 | .108 | - .006 | .030 | - .134 | .066 |
| 12. Often fights with other children or bullies them | .099 | .093 | .279 | .057 | **.455** | .066 | .201 | .058 |
| 13. Often unhappy, depressed or tearful | .150 | .072 | **.503** | .076 | .092 | .076 | .030 | .052 |
| 15. Easily distracted, concentration wanders | **.560** | .082 | .060 | .060 | .171 | .080 | .036 | .047 |
| 16. Nervous or clingy in new situations, easily loses confidence | - .025 | .050 | .172 | .073 | .277 | .068 | - .152 | .052 |
| 18. Often argumentative with adults | .062 | .082 | - .011 | .043 | **.659** | .082 | - .188 | .056 |
| 19. Picked on or bullied by other children | - .005 | .054 | **.511** | .058 | - .049 | .067 | .105 | .065 |
| 22. Can be spiteful to others | - .169 | .115 | .023 | .039 | **.852** | .066 | - .045 | .036 |
| 23. Gets along better with adults than with other children | .008 | .062 | **.400** | .055 | .144 | .067 | - .082 | .062 |
| 24. Many fears, easily scared | - .073 | .053 | .354 | .057 | .155 | .062 | .102 | .055 |
| 7. Generally well behaved, usually does what adults request # | .314 | .123 | - .235 | .078 | **.322** | .099 | .265 | .063 |
| 11. Has at least one good friend # | - .037 | .047 | .106 | .085 | - .088 | .086 | **.646** | .048 |
| 14. Generally liked by other children # | - .092 | .084 | .102 | .094 | .172 | .107 | **.692** | .058 |
| 21. Can stop and think things out before acting # | **.401** | .077 | - .099 | .054 | - .027 | .031 | **.531** | .051 |
| 25. Good attention span, sees work through to the end # | **.333** | .079 | - .044 | .044 | - .015 | .042 | **.510** | .048 |

*Note*. # = Reversed scored item. Bold typeface = primary loading. All primary loadings were *p* < .02.

S5.2

*4 Factor Structure of the Parent-Reported SDQ Difficulties Items for Children Aged 4-5 Years*

| SDQ Items | Factor 1 | | Factor 2 | | Factor 3 | | Factor 4 | |
| --- | --- | --- | --- | --- | --- | --- | --- | --- |
|  | Standardised Factor Loading | Standard Error | Standardised Factor Loading | Standard Error | Standardised Factor Loading | Standard Error | Standardised Factor Loading | Standard Error |
| 2. Restless, overactive, cannot stay still for long | .407 | .115 | **.621** | .061 | .027 | .041 | - .025 | .033 |
| 3. Often complains of headaches, stomach-aches or sickness | .378 | .099 | - .126 | .072 | .211 | .084 | - .039 | .077 |
| 5. Often loses temper | .274 | .071 | .226 | .068 | .290 | .065 | .030 | .043 |
| 6. Rather solitary, prefers to play alone | **.426** | .068 | - .038 | .065 | - .010 | .063 | .122 | .080 |
| 8. Many worries or often seems worried | **.686** | .066 | .022 | .059 | - .113 | .093 | .044 | .046 |
| 10. Constantly fidgeting or squirming | .414 | .124 | **.583** | .068 | .051 | .044 | - .097 | .047 |
| 12. Often fights with other children or bullies them | .186 | .086 | .090 | .071 | **.509** | .070 | .125 | .095 |
| 13. Often unhappy, depressed or tearful | **.717** | .083 | - .008 | .044 | .142 | .114 | .093 | .061 |
| 15. Easily distracted, concentration wanders | .143 | .132 | **.652** | .057 | .023 | .063 | - .011 | .036 |
| 16. Nervous or clingy in new situations, easily loses confidence | **.519** | .058 | .116 | .063 | - .083 | .075 | .047 | .051 |
| 18. Often argumentative with adults | - .007 | .061 | .015 | .052 | **.788** | .065 | - .046 | .056 |
| 19. Picked on or bullied by other children | .298 | .083 | .033 | .058 | **.373** | .083 | - .097 | .058 |
| 22. Can be spiteful to others | .006 | .051 | .006 | .057 | **.613** | .075 | .251 | .089 |
| 23. Gets along better with adults than with other children | .320 | .083 | - .075 | .069 | .234 | .096 | - .210 | .056 |
| 24. Many fears, easily scared | **.500** | .052 | - .011 | .059 | - .080 | .077 | .011 | .039 |
| 7. Generally well behaved, usually does what adults request # | - .053 | .034 | **.403** | .075 | **.314** | .086 | **.483** | .074 |
| 11. Has at least one good friend # | .036 | .039 | - .157 | .107 | .001 | .042 | **.878** | .080 |
| 14. Generally liked by other children # | .136 | .100 | - .052 | .048 | .168 | .087 | **.705** | .072 |
| 21. Can stop and think things out before acting # | - .097 | .086 | **.491** | .061 | - .025 | .044 | **.442** | .076 |
| 25. Good attention span, sees work through to the end # | .195 | .082 | .357 | .084 | - .095 | .068 | **.411** | .053 |

*Note*. # = Reversed scored item. Bold typeface = primary loading. All primary loadings were *p* < .02.

S5.3

*4 Factor Structure of the Parent-Reported SDQ Difficulties Items for Children Aged 6-7 Years*

| SDQ Items | Factor 1 | | Factor 2 | | Factor 3 | | Factor 4 | |
| --- | --- | --- | --- | --- | --- | --- | --- | --- |
|  | Standardised Factor Loading | Standard Error | Standardised Factor Loading | Standard Error | Standardised Factor Loading | Standard Error | Standardised Factor Loading | Standard Error |
| 2. Restless, overactive, cannot stay still for long | **.756** | .050 | .015 | .033 | .010 | .040 | .038 | .045 |
| 3. Often complains of headaches, stomach-aches or sickness | .048 | .041 | **.412** | .053 | .027 | .048 | .027 | .041 |
| 5. Often loses temper | .062 | .050 | .149 | .060 | **.418** | .051 | .054 | .043 |
| 6. Rather solitary, prefers to play alone | - .061 | .049 | .354 | .042 | .014 | .047 | .041 | .043 |
| 8. Many worries or often seems worried | - .039 | .046 | **.619** | .058 | - .099 | .060 | .119 | .051 |
| 10. Constantly fidgeting or squirming | **.807** | .059 | .075 | .046 | .053 | .048 | - .054 | .046 |
| 12. Often fights with other children or bullies them | .020 | .033 | .153 | .063 | **.622** | .060 | - .014 | .037 |
| 13. Often unhappy, depressed or tearful | .055 | .048 | **.601** | .041 | .135 | .069 | .061 | .045 |
| 15. Easily distracted, concentration wanders | **.475** | .057 | .021 | .037 | .163 | .057 | .146 | .055 |
| 16. Nervous or clingy in new situations, easily loses confidence | .078 | .055 | **.539** | .049 | .005 | .048 | - .094 | .044 |
| 18. Often argumentative with adults | .063 | .055 | .013 | .041 | **.618** | .060 | - .013 | .040 |
| 19. Picked on or bullied by other children | .019 | .047 | .267 | .059 | .333 | .057 | - .001 | .044 |
| 22. Can be spiteful to others | - .158 | .070 | - .037 | .050 | **.672** | .067 | .010 | .039 |
| 23. Gets along better with adults than with other children | .086 | .073 | .281 | .054 | .153 | .054 | - .142 | .040 |
| 24. Many fears, easily scared | .039 | .046 | **.603** | .045 | .022 | .047 | - .011 | .028 |
| 7. Generally well behaved, usually does what adults request # | .072 | .062 | - .065 | .052 | **.376** | .060 | **.400** | .051 |
| 11. Has at least one good friend # | - .044 | .059 | .079 | .069 | - .019 | .073 | **.525** | .071 |
| 14. Generally liked by other children # | - .172 | .122 | .086 | .065 | .186 | .094 | **.638** | .067 |
| 21. Can stop and think things out before acting # | .277 | .128 | - .239 | .051 | .010 | .032 | **.586** | .056 |
| 25. Good attention span, sees work through to the end # | .271 | .113 | .024 | .027 | - .066 | .051 | **.581** | .052 |

*Note*. # = Reversed scored item. Bold typeface = primary loading. All primary loadings were *p* < .02.

S5.4

*4 Factor Structure of the Parent-Reported SDQ Difficulties Items for Children Aged 8-9 Years*

| SDQ Items | Factor 1 | | Factor 2 | | Factor 3 | | Factor 4 | |
| --- | --- | --- | --- | --- | --- | --- | --- | --- |
|  | Standardised Factor Loading | Standard Error | Standardised Factor Loading | Standard Error | Standardised Factor Loading | Standard Error | Standardised Factor Loading | Standard Error |
| 2. Restless, overactive, cannot stay still for long | **.645** | .054 | .260 | .097 | .135 | .068 | - .048 | .032 |
| 3. Often complains of headaches, stomach-aches or sickness | .050 | .051 | **.510** | .044 | .067 | .066 | - .047 | .046 |
| 5. Often loses temper | .152 | .054 | .149 | .043 | **.446** | .045 | .066 | .044 |
| 6. Rather solitary, prefers to play alone | - .076 | .047 | .298 | .049 | .036 | .058 | .161 | .048 |
| 8. Many worries or often seems worried | .036 | .044 | **.561** | .049 | .012 | .044 | .223 | .050 |
| 10. Constantly fidgeting or squirming | **.659** | .065 | .414 | .093 | - .003 | .031 | - .042 | .027 |
| 12. Often fights with other children or bullies them | .141 | .078 | .026 | .035 | **.678** | .048 | .064 | .054 |
| 13. Often unhappy, depressed or tearful | - .066 | .041 | **.478** | .044 | .312 | .063 | .223 | .058 |
| 15. Easily distracted, concentration wanders | **.465** | .052 | .229 | .074 | .140 | .063 | .107 | .050 |
| 16. Nervous or clingy in new situations, easily loses confidence | .118 | .069 | **.597** | .045 | - .035 | .037 | - .003 | .031 |
| 18. Often argumentative with adults | .143 | .069 | .034 | .044 | **.694** | .051 | - .136 | .057 |
| 19. Picked on or bullied by other children | .011 | .047 | .289 | .043 | .336 | .048 | .025 | .037 |
| 22. Can be spiteful to others | - .028 | .038 | - .025 | .042 | **.682** | .054 | - .183 | .074 |
| 23. Gets along better with adults than with other children | - .137 | .050 | .299 | .044 | .254 | .061 | .005 | .039 |
| 24. Many fears, easily scared | .082 | .058 | **.617** | .043 | - .030 | .044 | - .022 | .034 |
| 7. Generally well behaved, usually does what adults request # | .253 | .078 | - .130 | .047 | **.378** | .058 | **.365** | .055 |
| 11. Has at least one good friend # | .023 | .034 | .124 | .064 | - .033 | .046 | **.730** | .052 |
| 14. Generally liked by other children # | - .086 | .068 | .028 | .024 | .215 | .088 | **.736** | .057 |
| 21. Can stop and think things out before acting # | **.474** | .056 | - .212 | .048 | .028 | .029 | **.396** | .050 |
| 25. Good attention span, sees work through to the end # | **.516** | .053 | .002 | .026 | - .110 | .058 | **.463** | .047 |

*Note*. # = Reversed scored item. Bold typeface = primary loading. All primary loadings were *p* < .02.

S5.5

*4 Factor Structure of the Parent-Reported SDQ Difficulties Items for Children Aged 10-12 Years*

| SDQ Items | Factor 1 | | Factor 2 | | Factor 3 | | Factor 4 | |
| --- | --- | --- | --- | --- | --- | --- | --- | --- |
|  | Standardised Factor Loading | Standard Error | Standardised Factor Loading | Standard Error | Standardised Factor Loading | Standard Error | Standardised Factor Loading | Standard Error |
| 2. Restless, overactive, cannot stay still for long | .325 | .069 | **.627** | .048 | .036 | .028 | - .026 | .028 |
| 3. Often complains of headaches, stomach-aches or sickness | **.433** | .047 | .016 | .044 | .061 | .059 | - .095 | .044 |
| 5. Often loses temper | .393 | .047 | .127 | .039 | **.323** | .056 | .071 | .028 |
| 6. Rather solitary, prefers to play alone | .351 | .042 | - .098 | .046 | .094 | .055 | .095 | .043 |
| 8. Many worries or often seems worried | **.705** | .042 | .033 | .034 | - .151 | .065 | .106 | .048 |
| 10. Constantly fidgeting or squirming | .376 | .082 | **.654** | .046 | .044 | .032 | - .035 | .025 |
| 12. Often fights with other children or bullies them | .177 | .061 | .069 | .044 | **.570** | .060 | .070 | .045 |
| 13. Often unhappy, depressed or tearful | **.586** | .058 | - .002 | .031 | .212 | .070 | .135 | .040 |
| 15. Easily distracted, concentration wanders | .274 | .058 | **.427** | .053 | .200 | .050 | .013 | .030 |
| 16. Nervous or clingy in new situations, easily loses confidence | **.678** | .035 | .088 | .044 | - .060 | .052 | - .079 | .034 |
| 18. Often argumentative with adults | .029 | .036 | .075 | .048 | **.737** | .050 | - .027 | .030 |
| 19. Picked on or bullied by other children | .311 | .049 | - .114 | .038 | **.463** | .054 | .035 | .031 |
| 22. Can be spiteful to others | - .095 | .059 | .037 | .045 | **.746** | .077 | - .023 | .039 |
| 23. Gets along better with adults than with other children | .286 | .043 | - .128 | .050 | .256 | .060 | - .108 | .044 |
| 24. Many fears, easily scared | **.721** | .033 | - .044 | .040 | - .013 | .038 | - .073 | .039 |
| 7. Generally well behaved, usually does what adults request # | - .033 | .036 | .286 | .052 | .156 | .050 | **.478** | .049 |
| 11. Has at least one good friend # | .052 | .039 | - .067 | .042 | .000 | .033 | **.790** | .044 |
| 14. Generally liked by other children # | .051 | .047 | - .069 | .037 | .056 | .063 | **.748** | .041 |
| 21. Can stop and think things out before acting # | - .131 | .040 | **.406** | .046 | .022 | .030 | **.509** | .046 |
| 25. Good attention span, sees work through to the end # | .013 | .016 | **.558** | .053 | - .175 | .047 | **.563** | .045 |

*Note*. # = Reversed scored item. Bold typeface = primary loading. All primary loadings were *p* < .02.

S5.6

*4 Factor Structure of the Parent-Reported SDQ Difficulties Items for Children Aged 13-14 Years*

| SDQ Items | Factor 1 | | Factor 2 | | Factor 3 | | Factor 4 | |
| --- | --- | --- | --- | --- | --- | --- | --- | --- |
|  | Standardised Factor Loading | Standard Error | Standardised Factor Loading | Standard Error | Standardised Factor Loading | Standard Error | Standardised Factor Loading | Standard Error |
| 2. Restless, overactive, cannot stay still for long | .073 | .077 | **.558** | .098 | .215 | .116 | - .039 | .064 |
| 3. Often complains of headaches, stomach-aches or sickness | .452 | .085 | .125 | .113 | - .001 | .092 | .061 | .079 |
| 5. Often loses temper | .228 | .094 | **.438** | .101 | .038 | .090 | .027 | .074 |
| 6. Rather solitary, prefers to play alone | **.484** | .078 | - .016 | .071 | - .007 | .096 | .160 | .091 |
| 8. Many worries or often seems worried | **.765** | .061 | - .017 | .050 | .135 | .120 | - .085 | .063 |
| 10. Constantly fidgeting or squirming | - .010 | .052 | **.767** | .093 | .065 | .109 | - .016 | .047 |
| 12. Often fights with other children or bullies them | .152 | .094 | .331 | .125 | **.417** | .113 | .117 | .080 |
| 13. Often unhappy, depressed or tearful | **.668** | .090 | .163 | .113 | .131 | .103 | - .003 | .066 |
| 15. Easily distracted, concentration wanders | - .048 | .052 | **.641** | .087 | .089 | .092 | .108 | .082 |
| 16. Nervous or clingy in new situations, easily loses confidence | .398 | .100 | **.451** | .095 | - .162 | .096 | - .016 | .060 |
| 18. Often argumentative with adults | - .019 | .061 | - .024 | .048 | **.955** | .089 | - .037 | .054 |
| 19. Picked on or bullied by other children | .448 | .078 | .062 | .088 | .243 | .112 | - .012 | .065 |
| 22. Can be spiteful to others | .008 | .078 | .095 | .115 | **.622** | .139 | .226 | .109 |
| 23. Gets along better with adults than with other children | .267 | .102 | .099 | .105 | .142 | .135 | - .268 | .070 |
| 24. Many fears, easily scared | **.672** | .075 | .284 | .141 | - .249 | .140 | .001 | .045 |
| 7. Generally well behaved, usually does what adults request # | .075 | .080 | .080 | .122 | .218 | .095 | **.553** | .070 |
| 11. Has at least one good friend # | **.519** | .105 | - .109 | .113 | - .006 | .064 | **.441** | .087 |
| 14. Generally liked by other children # | .394 | .130 | - .117 | .108 | .027 | .083 | **.646** | .098 |
| 21. Can stop and think things out before acting # | .130 | .087 | - .192 | .137 | - .004 | .059 | **- .722** | .068 |
| 25. Good attention span, sees work through to the end # | .024 | .039 | - .148 | .146 | .032 | .084 | **- .765** | .066 |

*Note*. # = Reversed scored item. Bold typeface = primary loading. All primary loadings were *p* < .02.

S5.7

*2 Factor Structure of the Teacher-Reported SDQ Difficulties Items for Children Aged 2-4 Years*

| SDQ Items | Factor 1 | | Factor 2 | |
| --- | --- | --- | --- | --- |
|  | Standardised Factor Loading | Standard Error | Standardised Factor Loading | Standard Error |
| 2. Restless, overactive, cannot stay still for long | **.918** | .021 | .028 | .046 |
| 3. Often complains of headaches, stomach-aches or sickness | **- .370** | .147 | **.818** | .094 |
| 5. Often loses temper | **.723** | .052 | .035 | .094 |
| 6. Rather solitary, prefers to play alone | .009 | .025 | **.665** | .054 |
| 8. Many worries or often seems worried | .103 | .124 | **.833** | .049 |
| 10. Constantly fidgeting or squirming | **.943** | .019 | - .001 | .009 |
| 12. Often fights with other children or bullies them | **.895** | .043 | - .275 | .091 |
| 13. Often unhappy, depressed or tearful | .117 | .124 | **.876** | .048 |
| 15. Easily distracted, concentration wanders | **.816** | .033 | .180 | .044 |
| 16. Nervous or clingy in new situations, easily loses confidence | - .132 | .113 | **.755** | .055 |
| 18. Often argumentative with adults | **.864** | .037 | - .126 | .083 |
| 19. Picked on or bullied by other children | **.643** | .121 | **- .297** | .093 |
| 22. Can be spiteful to others | **.845** | .045 | - .254 | .090 |
| 23. Gets along better with adults than with other children | .087 | .100 | **.473** | .066 |
| 24. Many fears, easily scared | - .151 | .110 | **.895** | .050 |
| 7. Generally well behaved, usually does what adults request # | **.709** | .044 | .204 | .081 |
| 11. Has at least one good friend # | .171 | .080 | **.668** | .051 |
| 14. Generally liked by other children # | **.541** | .071 | **.416** | .085 |
| 21. Can stop and think things out before acting # | **.630** | .054 | **.325** | .054 |
| 25. Good attention span, sees work through to the end # | **.551** | .054 | **.340** | .065 |

*Note*. # = Reversed scored item. Bold typeface = primary loading. All primary loadings were *p* < .02.

S5.8

*4 Factor Structure of the Teacher-Reported SDQ Difficulties Items for Children Aged 4-5 Years*

| SDQ Items | Factor 1 | | Factor 2 | | Factor 3 | | Factor 4 | |
| --- | --- | --- | --- | --- | --- | --- | --- | --- |
|  | Standardised Factor Loading | Standard Error | Standardised Factor Loading | Standard Error | Standardised Factor Loading | Standard Error | Standardised Factor Loading | Standard Error |
| 2. Restless, overactive, cannot stay still for long | .268 | .058 | **.788** | .041 | .012 | .026 | - .081 | .052 |
| 3. Often complains of headaches, stomach-aches or sickness | .124 | .122 | - .050 | .070 | **.566** | .081 | - .172 | .142 |
| 5. Often loses temper | **.769** | .066 | .064 | .062 | - .035 | .070 | .010 | .070 |
| 6. Rather solitary, prefers to play alone | .021 | .043 | - .078 | .072 | .325 | .085 | **.540** | .083 |
| 8. Many worries or often seems worried | .068 | .085 | .039 | .055 | **.747** | .056 | - .117 | .094 |
| 10. Constantly fidgeting or squirming | .105 | .081 | **.845** | .057 | .144 | .051 | - .162 | .073 |
| 12. Often fights with other children or bullies them | **.859** | .080 | .065 | .072 | - .138 | .079 | - .023 | .059 |
| 13. Often unhappy, depressed or tearful | .217 | .102 | .004 | .049 | **.709** | .045 | .002 | .048 |
| 15. Easily distracted, concentration wanders | .022 | .046 | **.849** | .040 | .204 | .063 | .050 | .053 |
| 16. Nervous or clingy in new situations, easily loses confidence | - .159 | .088 | .225 | .083 | **.772** | .050 | .094 | .063 |
| 18. Often argumentative with adults | **.862** | .049 | .036 | .045 | .011 | .044 | - .190 | .079 |
| 19. Picked on or bullied by other children | .416 | .167 | - .165 | .143 | .162 | .116 | .118 | .113 |
| 22. Can be spiteful to others | **.768** | .073 | .020 | .050 | .059 | .064 | - .113 | .094 |
| 23. Gets along better with adults than with other children | **.610** | .164 | - .488 | .111 | .111 | .115 | .242 | .129 |
| 24. Many fears, easily scared | .008 | .056 | - .028 | .057 | **.827** | .045 | .005 | .050 |
| 7. Generally well behaved, usually does what adults request # | .574 | .078 | .403 | .060 | - .096 | .042 | .201 | .063 |
| 11. Has at least one good friend # | .305 | .128 | .040 | .059 | .015 | .048 | **.642** | .065 |
| 14. Generally liked by other children # | **.584** | .110 | .028 | .045 | .086 | .073 | **.387** | .076 |
| 21. Can stop and think things out before acting # | .251 | .070 | **.544** | .054 | - .053 | .037 | .251 | .059 |
| 25. Good attention span, sees work through to the end # | - .017 | .033 | **.773** | .049 | .260 | .067 | .204 | .067 |

*Note*. # = Reversed scored item. Bold typeface = primary loading. All primary loadings were *p* < .02.

S5.9

*4 Factor Structure of the Teacher-Reported SDQ Difficulties Items for Children Aged 6-7 Years*

| SDQ Items | Factor 1 | | Factor 2 | | Factor 3 | | Factor 4 | |
| --- | --- | --- | --- | --- | --- | --- | --- | --- |
|  | Standardised Factor Loading | Standard Error | Standardised Factor Loading | Standard Error | Standardised Factor Loading | Standard Error | Standardised Factor Loading | Standard Error |
| 2. Restless, overactive, cannot stay still for long | .122 | .080 | **.869** | .054 | - .035 | .025 | - .083 | .043 |
| 3. Often complains of headaches, stomach-aches or sickness | .120 | .111 | .082 | .089 | **.458** | .055 | .069 | .099 |
| 5. Often loses temper | **.780** | .067 | .132 | .071 | .095 | .055 | - .076 | .064 |
| 6. Rather solitary, prefers to play alone | - .017 | .019 | .075 | .061 | .293 | .072 | **.638** | .085 |
| 8. Many worries or often seems worried | .135 | .093 | - .102 | .055 | **.816** | .039 | .093 | .065 |
| 10. Constantly fidgeting or squirming | .164 | .074 | **.818** | .052 | .054 | .027 | - .039 | .036 |
| 12. Often fights with other children or bullies them | **.945** | .049 | .011 | .032 | - .062 | .037 | - .218 | .073 |
| 13. Often unhappy, depressed or tearful | .409 | .090 | - .058 | .046 | **.679** | .045 | .028 | .034 |
| 15. Easily distracted, concentration wanders | - .029 | .038 | **.889** | .041 | .021 | .028 | .130 | .057 |
| 16. Nervous or clingy in new situations, easily loses confidence | - .126 | .081 | .287 | .068 | **.766** | .033 | - .056 | .055 |
| 18. Often argumentative with adults | **.819** | .054 | .065 | .053 | .012 | .037 | - .073 | .065 |
| 19. Picked on or bullied by other children | .586 | .073 | - .004 | .048 | .299 | .057 | - .005 | .058 |
| 22. Can be spiteful to others | **.765** | .074 | .060 | .075 | .101 | .086 | .085 | .062 |
| 23. Gets along better with adults than with other children | .372 | .101 | - .160 | .082 | .199 | .071 | .207 | .095 |
| 24. Many fears, easily scared | - .022 | .023 | .065 | .061 | **.948** | .027 | - .021 | .043 |
| 7. Generally well behaved, usually does what adults request # | .602 | .060 | .366 | .056 | - .078 | .032 | .030 | .039 |
| 11. Has at least one good friend # | .448 | .117 | - .020 | .027 | - .039 | .030 | **.616** | .080 |
| 14. Generally liked by other children # | **.713** | .062 | .050 | .049 | .023 | .032 | .254 | .058 |
| 21. Can stop and think things out before acting # | .343 | .053 | **.516** | .051 | - .134 | .042 | .137 | .054 |
| 25. Good attention span, sees work through to the end # | .033 | .039 | **.737** | .041 | .066 | .027 | .224 | .060 |

*Note*. # = Reversed scored item. Bold typeface = primary loading. All primary loadings were *p* < .02.

S5.10

*4 Factor Structure of the Teacher-Reported SDQ Difficulties Items for Children Aged 8-9 Years*

| SDQ Items | Factor 1 | | Factor 2 | | Factor 3 | | Factor 4 | |
| --- | --- | --- | --- | --- | --- | --- | --- | --- |
|  | Standardised Factor Loading | Standard Error | Standardised Factor Loading | Standard Error | Standardised Factor Loading | Standard Error | Standardised Factor Loading | Standard Error |
| 2. Restless, overactive, cannot stay still for long | .336 | .084 | .017 | .020 | **.782** | .050 | - .069 | .032 |
| 3. Often complains of headaches, stomach-aches or sickness | .112 | .060 | **.443** | .047 | .080 | .050 | .116 | .068 |
| 5. Often loses temper | **.775** | .058 | .079 | .032 | .171 | .063 | - .016 | .046 |
| 6. Rather solitary, prefers to play alone | - .193 | .093 | .391 | .069 | - .026 | .034 | **.547** | .053 |
| 8. Many worries or often seems worried | .055 | .048 | **.839** | .034 | .028 | .030 | - .038 | .032 |
| 10. Constantly fidgeting or squirming | .244 | .080 | .029 | .022 | **.809** | .045 | - .014 | .024 |
| 12. Often fights with other children or bullies them | **.903** | .026 | .000 | .028 | .030 | .037 | - .010 | .033 |
| 13. Often unhappy, depressed or tearful | .408 | .063 | **.660** | .033 | - .009 | .022 | - .004 | .046 |
| 15. Easily distracted, concentration wanders | - .045 | .029 | .031 | .025 | **.800** | .038 | **.351** | .057 |
| 16. Nervous or clingy in new situations, easily loses confidence | - .004 | .030 | **.745** | .029 | .158 | .048 | .018 | .038 |
| 18. Often argumentative with adults | **.845** | .031 | .031 | .031 | .047 | .041 | .050 | .044 |
| 19. Picked on or bullied by other children | .452 | .070 | .365 | .049 | - .178 | .051 | .093 | .049 |
| 22. Can be spiteful to others | **.852** | .048 | .154 | .069 | - .115 | .060 | - .021 | .054 |
| 23. Gets along better with adults than with other children | .053 | .041 | .320 | .060 | - .212 | .049 | .263 | .060 |
| 24. Many fears, easily scared | - .092 | .050 | **.894** | .028 | .062 | .045 | - .003 | .031 |
| 7. Generally well behaved, usually does what adults request # | **.538** | .046 | - .127 | .034 | **.404** | .053 | .258 | .044 |
| 11. Has at least one good friend # | .085 | .068 | .171 | .057 | - .020 | .032 | **.743** | .054 |
| 14. Generally liked by other children # | .424 | .063 | - .020 | .029 | .050 | .034 | **.571** | .052 |
| 21. Can stop and think things out before acting # | .416 | .040 | - .053 | .031 | .396 | .049 | .285 | .047 |
| 25. Good attention span, sees work through to the end # | .027 | .023 | .049 | .021 | **.663** | .041 | **.474** | .050 |

*Note*. # = Reversed scored item. Bold typeface = primary loading. All primary loadings were *p* < .02.

| SDQ Items | Factor 1 | | Factor 2 | | Factor 3 | | Factor 4 | |
| --- | --- | --- | --- | --- | --- | --- | --- | --- |
|  | Standardised Factor Loading | Standard Error | Standardised Factor Loading | Standard Error | Standardised Factor Loading | Standard Error | Standardised Factor Loading | Standard Error |
| 2. Restless, overactive, cannot stay still for long | **.843** | .068 | .170 | .070 | .024 | .015 | - .352 | .044 |
| 3. Often complains of headaches, stomach-aches or sickness | .101 | .065 | .191 | .111 | **.464** | .064 | - .026 | .044 |
| 5. Often loses temper | .333 | .094 | **.584** | .093 | .137 | .050 | - .067 | .038 |
| 6. Rather solitary, prefers to play alone | .002 | .045 | .048 | .071 | .383 | .081 | **.424** | .058 |
| 8. Many worries or often seems worried | - .023 | .036 | .165 | .174 | **.874** | .075 | - .086 | .049 |
| 10. Constantly fidgeting or squirming | **.871** | .042 | .060 | .054 | .110 | .026 | - .311 | .043 |
| 12. Often fights with other children or bullies them | .141 | .122 | **.834** | .090 | - .030 | .049 | - .038 | .034 |
| 13. Often unhappy, depressed or tearful | - .022 | .030 | .370 | .157 | **.697** | .076 | .005 | .037 |
| 15. Easily distracted, concentration wanders | **.971** | .039 | - .073 | .051 | - .018 | .030 | - .003 | .028 |
| 16. Nervous or clingy in new situations, easily loses confidence | .321 | .083 | - .228 | .133 | **.720** | .055 | .049 | .037 |
| 18. Often argumentative with adults | .144 | .099 | **.796** | .077 | - .015 | .038 | - .007 | .032 |
| 19. Picked on or bullied by other children | - .049 | .058 | .456 | .109 | .343 | .072 | .149 | .048 |
| 22. Can be spiteful to others | .002 | .049 | **.774** | .051 | .019 | .054 | .051 | .058 |
| 23. Gets along better with adults than with other children | - .065 | .057 | .115 | .098 | .286 | .081 | .336 | .062 |
| 24. Many fears, easily scared | .191 | .090 | - .091 | .132 | **.812** | .060 | .028 | .034 |
| 7. Generally well behaved, usually does what adults request # | .480 | .077 | **.515** | .069 | - .092 | .046 | .081 | .037 |
| 11. Has at least one good friend # | .035 | .033 | .290 | .107 | .218 | .096 | **.551** | .061 |
| 14. Generally liked by other children # | .182 | .080 | .487 | .113 | .078 | .074 | **.414** | .046 |
| 21. Can stop and think things out before acting # | **.554** | .057 | .317 | .062 | - .118 | .040 | .100 | .037 |
| 25. Good attention span, sees work through to the end # | **.872** | .033 | - .014 | .039 | - .016 | .027 | .139 | .046 |

S5.11

*4 Factor Structure of the Teacher-Reported SDQ Difficulties Items for Children Aged 10-12 Years*

*Note*. # = Reversed scored item. Bold typeface = primary loading. All primary loadings were *p* < .02.

S5.12

*4 Factor Structure of the Teacher-Reported SDQ Difficulties Items for Children Aged 13-15 Years*

| SDQ Items | Factor 1 | | Factor 2 | | Factor 3 | | Factor 4 | |
| --- | --- | --- | --- | --- | --- | --- | --- | --- |
|  | Standardised Factor Loading | Standard Error | Standardised Factor Loading | Standard Error | Standardised Factor Loading | Standard Error | Standardised Factor Loading | Standard Error |
| 2. Restless, overactive, cannot stay still for long | **.976** | .068 | - .026 | .037 | .032 | .047 | - .095 | .185 |
| 3. Often complains of headaches, stomach-aches or sickness | .251 | .120 | .585 | .082 | - .007 | .093 | .112 | .150 |
| 5. Often loses temper | .399 | .128 | .002 | .044 | **.643** | .077 | - .013 | .059 |
| 6. Rather solitary, prefers to play alone | .020 | .065 | **.763** | .098 | - .011 | .066 | **- .388** | .086 |
| 8. Many worries or often seems worried | .020 | .079 | **.897** | .042 | .042 | .081 | .002 | .094 |
| 10. Constantly fidgeting or squirming | **1.036** | .057 | - .013 | .023 | - .036 | .047 | - .144 | .204 |
| 12. Often fights with other children or bullies them | .252 | .161 | - .068 | .053 | **.752** | .107 | .019 | .058 |
| 13. Often unhappy, depressed or tearful | .141 | .109 | **.792** | .048 | .130 | .112 | - .003 | .053 |
| 15. Easily distracted, concentration wanders | **.781** | .093 | .076 | .081 | .001 | .050 | .330 | .181 |
| 16. Nervous or clingy in new situations, easily loses confidence | .084 | .123 | **.627** | .077 | - .148 | .113 | .211 | .138 |
| 18. Often argumentative with adults | .248 | .154 | - .009 | .037 | .530 | .103 | **.452** | .089 |
| 19. Picked on or bullied by other children | - .141 | .094 | .587 | .085 | .419 | .094 | - .031 | .065 |
| 22. Can be spiteful to others | - .020 | .037 | .202 | .106 | **.654** | .106 | **.554** | .123 |
| 23. Gets along better with adults than with other children | - .007 | .152 | .324 | .091 | - .009 | .101 | - .346 | .114 |
| 24. Many fears, easily scared | - .073 | .104 | **.847** | .052 | - .024 | .058 | .210 | .137 |
| 7. Generally well behaved, usually does what adults request # | .393 | .140 | - .072 | .055 | **.601** | .091 | .261 | .087 |
| 11. Has at least one good friend # | - .024 | .044 | .406 | .160 | .503 | .111 | **- .557** | .113 |
| 14. Generally liked by other children # | .064 | .120 | .288 | .117 | **.733** | .101 | - .137 | .112 |
| 21. Can stop and think things out before acting # | **.482** | .098 | .025 | .058 | .423 | .064 | .149 | .097 |
| 25. Good attention span, sees work through to the end # | **.589** | .108 | .124 | .070 | .170 | .086 | .293 | .154 |

*Note*. # = Reversed scored item. Bold typeface = primary loading. All primary loadings were *p* < .02.
