# Supplementary material for: Against the use of the Strengths and Difficulties Questionnaire for Aboriginal and Torres Strait Islander children aged 2–15 years
Source: Aust N Z J Psychiatry. 2023 Mar 28;57(10):1343–58. doi: 10.1177/00048674231161504 (PMC10517593; doi:10.1177/00048674231161504)
Supplement: sj-docx-6-anp-10.1177_00048674231161504 – Supplemental material for Against the use of the Strengths and Difficulties Questionnaire for Aboriginal and Torres Strait Islander children aged 2–15 years [file sj-docx-6-anp-10.1177_00048674231161504.docx]

S6.1

*Bifactor ESEM General Factor Loadings for the Parent-Reported SDQ Difficulties Items – Age 2-4 Years*

| Item | Standardised factor loading | Standard error |
| --- | --- | --- |
| 2. Restless, overactive, cannot stay still for long | .628 | .039 |
| 3. Often complains of headaches, stomach-aches or sickness | .210 | .072 |
| 5. Often loses temper | .552 | .042 |
| 6. Rather solitary, prefers to play alone | .235 | .051 |
| 8. Many worries or often seems worried | .359 | .064 |
| 10. Constantly fidgeting or squirming | .591 | .045 |
| 12. Often fights with other children or bullies them | .638 | .035 |
| 13. Often unhappy, depressed or tearful | .426 | .056 |
| 15. Easily distracted, concentration wanders | .571 | .041 |
| 16. Nervous or clingy in new situations, easily loses confidence | .268 | .051 |
| 18. Often argumentative with adults | .602 | .054 |
| 19. Picked on or bullied by other children | .190 | .052 |
| 22. Can be spiteful to others | .650 | .059 |
| 23. Gets along better with adults than with other children | .321 | .046 |
| 24. Many fears, easily scared | .238 | .046 |
| 7. Generally well behaved, usually does what adults request # | .475 | .053 |
| 11. Has at least one good friend # | .087 | .097 |
| 14. Generally liked by other children # | .292 | .099 |
| 21. Can stop and think things out before acting # | .307 | .073 |
| 25. Good attention span, sees work through to the end # | .283 | .074 |

*Note*. # = Reversed scored item.

S6.2

*Bifactor ESEM General Factor Loadings for the Parent-Reported SDQ Difficulties Items – Age 4-5 Years*

| Item | Standardised factor loading | Standard error |
| --- | --- | --- |
| 2. Restless, overactive, cannot stay still for long | .741 | .046 |
| 3. Often complains of headaches, stomach-aches or sickness | .331 | .078 |
| 5. Often loses temper | .538 | .073 |
| 6. Rather solitary, prefers to play alone | .360 | .049 |
| 8. Many worries or often seems worried | .531 | .053 |
| 10. Constantly fidgeting or squirming | .692 | .041 |
| 12. Often fights with other children or bullies them | .504 | .061 |
| 13. Often unhappy, depressed or tearful | .684 | .057 |
| 15. Easily distracted, concentration wanders | .571 | .083 |
| 16. Nervous or clingy in new situations, easily loses confidence | .487 | .062 |
| 18. Often lies or cheats | .388 | .109 |
| 19. Picked on or bullied by other children | .429 | .097 |
| 22. Steals from home, school or elsewhere | .383 | .077 |
| 23. Gets along better with adults than with other children | .276 | .139 |
| 24. Many fears, easily scared | .414 | .113 |
| 7. Generally well behaved, usually does what adults request # | .504 | .170 |
| 11. Has at least one good friend # | .187 | .351 |
| 14. Generally liked by other children # **NON-SIG | .363 | .259 |
| 21. Thinks things out before acting # | .354 | .155 |
| 25. Good attention span, sees work through to the end # | .466 | .133 |

*Note*. # = Reversed scored item.

S6.3

*Bifactor ESEM General Factor Loadings for the Parent-Reported SDQ Difficulties Items – Age 6-7 Years*

| Item | Standardised factor loading | Standard error |
| --- | --- | --- |
| 2. Restless, overactive, cannot stay still for long | .663 | .208 |
| 3. Often complains of headaches, stomach-aches or sickness | .326 | .037 |
| 5. Often loses temper | .448 | .044 |
| 6. Rather solitary, prefers to play alone | .225 | .090 |
| 8. Many worries or often seems worried | .277 | .048 |
| 10. Constantly fidgeting or squirming | .690 | .251 |
| 12. Often fights with other children or bullies them | .572 | .041 |
| 13. Often unhappy, depressed or tearful | .525 | .036 |
| 15. Easily distracted, concentration wanders | .588 | .108 |
| 16. Nervous or clingy in new situations, easily loses confidence | .314 | .063 |
| 18. Often lies or cheats | .452 | .091 |
| 19. Picked on or bullied by other children | .459 | .066 |
| 22. Steals from home, school or elsewhere | .312 | .060 |
| 23. Gets along better with adults than with other children | .367 | .072 |
| 24. Many fears, easily scared | .376 | .052 |
| 7. Generally well behaved, usually does what adults request # | .513 | .074 |
| 11. Has at least one good friend # | .389 | .255 |
| 14. Generally liked by other children # | .479 | .280 |
| 21. Thinks things out before acting # | .406 | .045 |
| 25. Good attention span, sees work through to the end # | .519 | .067 |

*Note*. # = Reversed scored item.

S6.4

*Bifactor ESEM General Factor Loadings for the Parent-Reported SDQ Difficulties Items – Age 8-9 Years*

| Item | Standardised factor loading | Standard error |
| --- | --- | --- |
| 2. Restless, overactive, cannot stay still for long | .672 | .031 |
| 3. Often complains of headaches, stomach-aches or sickness | .336 | .044 |
| 5. Often loses temper | .566 | .032 |
| 6. Rather solitary, prefers to play alone | .257 | .033 |
| 8. Many worries or often seems worried | .445 | .037 |
| 10. Constantly fidgeting or squirming | .651 | .035 |
| 12. Often fights with other children or bullies them | .680 | .034 |
| 13. Often unhappy, depressed or tearful | .587 | .035 |
| 15. Easily distracted, concentration wanders | .607 | .029 |
| 16. Nervous or clingy in new situations, easily loses confidence | .364 | .036 |
| 18. Often lies or cheats | .581 | .049 |
| 19. Picked on or bullied by other children | .449 | .029 |
| 22. Steals from home, school or elsewhere | .432 | .060 |
| 23. Gets along better with adults than with other children | .375 | .043 |
| 24. Many fears, easily scared | .337 | .035 |
| 7. Generally well behaved, usually does what adults request # | .604 | .031 |
| 11. Has at least one good friend # | .491 | .059 |
| 14. Generally liked by other children # | .590 | .056 |
| 21. Thinks things out before acting # | .427 | .036 |
| 25. Good attention span, sees work through to the end # | .493 | .036 |

*Note*. # = Reversed scored item.

S6.5

*Bifactor ESEM General Factor Loadings for the Parent-Reported SDQ Difficulties Items – Age 10-12 Years*

| Item | Standardised factor loading | Standard error |
| --- | --- | --- |
| 2. Restless, overactive, cannot stay still for long | .686 | .065 |
| 3. Often complains of headaches, stomach-aches or sickness | .099 | .084 |
| 5. Often loses temper | .075 | .068 |
| 6. Rather solitary, prefers to play alone | .015 | .055 |
| 8. Many worries or often seems worried | - .011 | .068 |
| 10. Constantly fidgeting or squirming | .522 | .071 |
| 12. Often fights with other children or bullies them | .013 | .075 |
| 13. Often unhappy, depressed or tearful | .014 | .076 |
| 15. Easily distracted, concentration wanders | .269 | .064 |
| 16. Nervous or clingy in new situations, easily loses confidence | - .019 | .096 |
| 18. Often lies or cheats | - .083 | .093 |
| 19. Picked on or bullied by other children | - .043 | .078 |
| 22. Steals from home, school or elsewhere | - .140 | .094 |
| 23. Gets along better with adults than with other children | .065 | .089 |
| 24. Many fears, easily scared | - .161 | .107 |
| 7. Generally well behaved, usually does what adults request # | .035 | .071 |
| 11. Has at least one good friend # | - .101 | .117 |
| 14. Generally liked by other children # | - .058 | .115 |
| 21. Thinks things out before acting # | .008 | .098 |
| 25. Good attention span, sees work through to the end # | .157 | .099 |

*Note*. # = Reversed scored item.

S6.6

*Bifactor ESEM General Factor Loadings for the Parent-Reported SDQ Difficulties Items – Age 13-14 Years*

| Item | Standardised factor loading | Standard error |
| --- | --- | --- |
| 2. Restless, overactive, cannot stay still for long | .563 | .065 |
| 3. Often complains of headaches, stomach-aches or sickness | .492 | .053 |
| 5. Often loses temper | .526 | .059 |
| 6. Rather solitary, prefers to play alone | .455 | .061 |
| 8. Many worries or often seems worried | .694 | .047 |
| 10. Constantly fidgeting or squirming | .561 | .060 |
| 12. Often fights with other children or bullies them | .675 | .071 |
| 13. Often unhappy, depressed or tearful | .767 | .047 |
| 15. Easily distracted, concentration wanders | .519 | .061 |
| 16. Nervous or clingy in new situations, easily loses confidence | .609 | .057 |
| 18. Often lies or cheats | .573 | .075 |
| 19. Picked on or bullied by other children | .563 | .056 |
| 22. Steals from home, school or elsewhere | .583 | .094 |
| 23. Gets along better with adults than with other children | .259 | .076 |
| 24. Many fears, easily scared | .599 | .055 |
| 7. Generally well behaved, usually does what adults request # | .439 | .083 |
| 11. Has at least one good friend # | .491 | .097 |
| 14. Generally liked by other children # | .456 | .119 |
| 21. Thinks things out before acting # | - .342 | .086 |
| 25. Good attention span, sees work through to the end # | - .380 | .071 |

*Note*. # = Reversed scored item.

S6.7

*Bifactor ESEM General Factor Loadings for the Teacher-Reported SDQ Difficulties Items – Age 4-5 Years*

| Item | Standardised factor loading | Standard error |
| --- | --- | --- |
| 2. Restless, overactive, cannot stay still for long | .920 | .021 |
| 3. Often complains of headaches, stomach-aches or sickness | .228 | .068 |
| 5. Often loses temper | .755 | .029 |
| 6. Rather solitary, prefers to play alone | .148 | .088 |
| 8. Many worries or often seems worried | .323 | .053 |
| 10. Constantly fidgeting or squirming | .847 | .024 |
| 12. Often fights with other children or bullies them | .807 | .036 |
| 13. Often unhappy, depressed or tearful | .438 | .061 |
| 15. Easily distracted, concentration wanders | .846 | .024 |
| 16. Nervous or clingy in new situations, easily loses confidence | .324 | .065 |
| 18. Often lies or cheats | .797 | .050 |
| 19. Picked on or bullied by other children | .312 | .056 |
| 22. Steals from home, school or elsewhere | .705 | .057 |
| 23. Gets along better with adults than with other children | .206 | .070 |
| 24. Many fears, easily scared | .257 | .067 |
| 7. Generally well behaved, usually does what adults request # | .883 | .021 |
| 11. Has at least one good friend # | .427 | .077 |
| 14. Generally liked by other children # | .656 | .048 |
| 21. Thinks things out before acting # | .737 | .031 |
| 25. Good attention span, sees work through to the end # | .792 | .041 |

*Note*. # = Reversed scored item.

S6.8

*Bifactor ESEM General Factor Loadings for the Teacher-Reported SDQ Difficulties Items – Age 6-7 Years*

| Item | Standardised factor loading | Standard error |
| --- | --- | --- |
| 2. Restless, overactive, cannot stay still for long | .715 | .038 |
| 3. Often complains of headaches, stomach-aches or sickness | .286 | .064 |
| 5. Often loses temper | .874 | .025 |
| 6. Rather solitary, prefers to play alone | .294 | .063 |
| 8. Many worries or often seems worried | .241 | .054 |
| 10. Constantly fidgeting or squirming | .751 | .037 |
| 12. Often fights with other children or bullies them | .865 | .029 |
| 13. Often unhappy, depressed or tearful | .501 | .058 |
| 15. Easily distracted, concentration wanders | .650 | .034 |
| 16. Nervous or clingy in new situations, easily loses confidence | .203 | .053 |
| 18. Often lies or cheats | .831 | .024 |
| 19. Picked on or bullied by other children | .634 | .050 |
| 22. Steals from home, school or elsewhere | .818 | .041 |
| 23. Gets along better with adults than with other children | .372 | .060 |
| 24. Many fears, easily scared | .183 | .067 |
| 7. Generally well behaved, usually does what adults request # | .854 | .020 |
| 11. Has at least one good friend # | .617 | .048 |
| 14. Generally liked by other children # | .833 | .027 |
| 21. Thinks things out before acting # | .727 | .027 |
| 25. Good attention span, sees work through to the end # | .624 | .031 |

*Note*. # = Reversed scored item.

S6.9

*Bifactor ESEM General Factor Loadings for the Teacher-Reported SDQ Difficulties Items – Age 8-9 Years*

| Item | Standardised factor loading | Standard error |
| --- | --- | --- |
| 2. Restless, overactive, cannot stay still for long | .794 | .048 |
| 3. Often complains of headaches, stomach-aches or sickness | .372 | .051 |
| 5. Often loses temper | .857 | .017 |
| 6. Rather solitary, prefers to play alone | .221 | .047 |
| 8. Many worries or often seems worried | .336 | .039 |
| 10. Constantly fidgeting or squirming | .760 | .051 |
| 12. Often fights with other children or bullies them | .865 | .025 |
| 13. Often unhappy, depressed or tearful | .596 | .034 |
| 15. Easily distracted, concentration wanders | .705 | .043 |
| 16. Nervous or clingy in new situations, easily loses confidence | .376 | .051 |
| 18. Often lies or cheats | .870 | .023 |
| 19. Picked on or bullied by other children | .478 | .054 |
| 22. Steals from home, school or elsewhere | .769 | .045 |
| 23. Gets along better with adults than with other children | .145 | .051 |
| 24. Many fears, easily scared | .271 | .050 |
| 7. Generally well behaved, usually does what adults request # | .874 | .017 |
| 11. Has at least one good friend # | .512 | .055 |
| 14. Generally liked by other children # | .733 | .040 |
| 21. Thinks things out before acting # | .800 | .021 |
| 25. Good attention span, sees work through to the end # | .767 | .029 |

*Note*. # = Reversed scored item.

S6.10

*Bifactor ESEM General Factor Loadings for the Teacher-Reported SDQ Difficulties Items – Age 10-12 Years*

| Item | Standardised factor loading | Standard error |
| --- | --- | --- |
| 2. Restless, overactive, cannot stay still for long | .783 | .031 |
| 3. Often complains of headaches, stomach-aches or sickness | .420 | .043 |
| 5. Often loses temper | .841 | .019 |
| 6. Rather solitary, prefers to play alone | .274 | .043 |
| 8. Many worries or often seems worried | .448 | .045 |
| 10. Constantly fidgeting or squirming | .741 | .033 |
| 12. Often fights with other children or bullies them | .849 | .025 |
| 13. Often unhappy, depressed or tearful | .586 | .044 |
| 15. Easily distracted, concentration wanders | .799 | .033 |
| 16. Nervous or clingy in new situations, easily loses confidence | .380 | .047 |
| 18. Often lies or cheats | .847 | .023 |
| 19. Picked on or bullied by other children | .530 | .039 |
| 22. Steals from home, school or elsewhere | .729 | .037 |
| 23. Gets along better with adults than with other children | .206 | .046 |
| 24. Many fears, easily scared | .414 | .051 |
| 7. Generally well behaved, usually does what adults request # | .887 | .013 |
| 11. Has at least one good friend # | .530 | .038 |
| 14. Generally liked by other children # | .759 | .021 |
| 21. Thinks things out before acting # | .781 | .023 |
| 25. Good attention span, sees work through to the end # | .828 | .026 |

*Note*. # = Reversed scored item.

S6.11

*Bifactor ESEM General Factor Loadings for the Teacher-Reported SDQ Difficulties Items – Age 13-15 Years*

| Item | Standardised factor loading | Standard error |
| --- | --- | --- |
| 2. Restless, overactive, cannot stay still for long | .718 | .098 |
| 3. Often complains of headaches, stomach-aches or sickness | .452 | .086 |
| 5. Often loses temper | .848 | .035 |
| 6. Rather solitary, prefers to play alone | .062 | .093 |
| 8. Many worries or often seems worried | .367 | .085 |
| 10. Constantly fidgeting or squirming | .684 | .100 |
| 12. Often fights with other children or bullies them | .834 | .048 |
| 13. Often unhappy, depressed or tearful | .491 | .085 |
| 15. Easily distracted, concentration wanders | .799 | .069 |
| 16. Nervous or clingy in new situations, easily loses confidence | .268 | .086 |
| 18. Often lies or cheats | .897 | .027 |
| 19. Picked on or bullied by other children | .445 | .099 |
| 22. Steals from home, school or elsewhere | .915 | .056 |
| 23. Gets along better with adults than with other children | - .088 | .103 |
| 24. Many fears, easily scared | .333 | .103 |
| 7. Generally well behaved, usually does what adults request # | .940 | .023 |
| 11. Has at least one good friend # | .229 | .105 |
| 14. Generally liked by other children # | .704 | .066 |
| 21. Thinks things out before acting # | .822 | .037 |
| 25. Good attention span, sees work through to the end # | .795 | .056 |

*Note*. # = Reversed scored item.
